# Novel 2‑(2′-Benzothiazolyl)-benzimidazole-Based Iridium(III) Photocatalysts Exhibit Antiproliferative Effects in 2D and 3D Cancer Cells to Bypass Hypoxia-Induced Resistance

**DOI:** 10.1021/acs.jmedchem.5c03280

**Published:** 2026-03-16

**Authors:** Antonio Linero-Artiaga, Marie Svitelova, Vojtěch Novohradský, Venancio Rodríguez, Lenka Markova, Jana Kasparkova, Christoph Janiak, José Ruiz, Viktor Brabec

**Affiliations:** † Departamento de Química Inorgánica, Universidad de Murcia, and Murcia BioHealth Research Institute (IMIB-Arrixaca), E-30100 Murcia, Spain; ‡ Czech Academy of Sciences, Institute of Biophysics, Kralovopolska 135, CZ-61200 Brno, Czech Republic; § Department of Molecular Pharmacy, Faculty of Pharmacy, Masaryk University, Palackeho trida 1946/1, CZ-61200 Brno, Czech Republic; ∥ Department of Biophysics, Faculty of Science, Palacky University, Slechtitelu 27, CZ-78371 Olomouc, Czech Republic; ⊥ Institut für Anorganische Chemie und Strukturchemie, Heinrich-Heine-Universität Düsseldorf, D-40204 Düsseldorf, Germany

## Abstract

This study explores the therapeutic potential of seven bis-cyclometalated Ir­(III) complexes (**1–7**), derived from the 2,2′-(benzothiazolyl)­benzimidazole scaffold, as highly promising next-generation photoactivatable agents for type I and type II-guided photodynamic therapy (PDT) in lung and colorectal cancers. Their high phototoxicity in 2D and 3D cancer cell models, achieving IC_50_ values in the nanomolar region, was closely linked to the generation of singlet oxygen and type I reactive oxygen species (ROS) and the photooxidation of NADH, with complex **4** identified as the strongest ROS inducer and the most photocytotoxic complex. Notably, the iridium complexes proved to maintain their phototoxicity in hypoxic conditions. Using 3D spheroids, complex **4** demonstrated deep tissue penetration sought to overcome PDT limitations in solid tumors. Overall, the synthesized complexes showcase high efficacy and favorable pharmacological profiles, positioning them as promising candidates for the ROS-guided photodynamic treatment of cancers, including those located within hypoxic environments.

## Graphical abstract

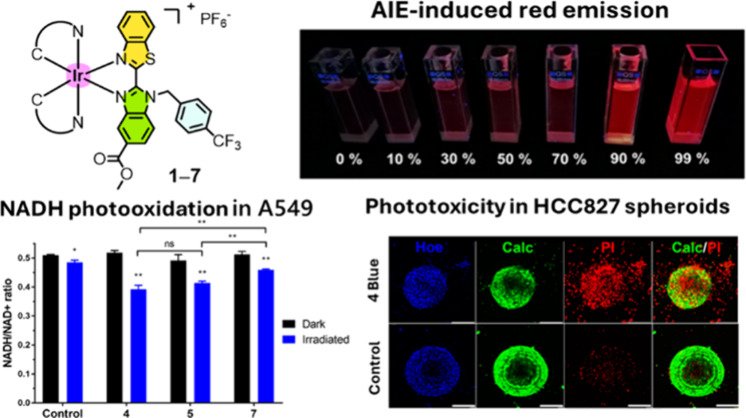

## Introduction

Photodynamic therapy (PDT) is a clinically approved, minimally invasive, selective light-based and oxygen-mediated medical technique employed to eradicate cancer and tumor vasculature by activating light-responsive drugs, known as photosensitizers (PSs).^1–4^

Transition metal complexes are emerging as potent PSs for PDT due to their superior photophysical and photochemical properties (i.e., excellent photostability, low systemic toxicity or tunable structures for optimized biological activity).^5–8^ A notable example is the Ru­(II) complex TLD1433, which has advanced to Phase II clinical trials for treating nonmuscle invasive bladder cancer using green light.^9^

In this sense, iridium­(III) complexes have particularly garnered much attention due to their long triplet-excited lifetimes and diverse photosensitization mechanisms.^10–18^ The heavy metal effect displayed by iridium centers enhances spin–orbit coupling (SOC) phenomena, promoting an efficient intersystem crossing (ISC) to their triplet excited states, where the PSs undergo two major photochemical reactions: type I pathway, based on the interaction with surrounding biomolecules in the presence of oxygen via electron/proton transfer processes, leading to the formation of highly cytotoxic ROS species such as superoxide radical anions (O_2_^·–^), hydroxyl radicals (·OH) or hydrogen peroxide (H_2_O_2_)^19^; and type II pathway, where the triplet–triplet nature of the energy transfer between the excited photosensitizer and ground-state oxygen molecules (^3^O_2_) ensues the generation of highly cytotoxic singlet oxygen molecules.^20^

Despite the numerous advantages associated with PDT, the insufficient oxygenation in certain tumor microenvironments limits the therapeutic potential of the PS activation. To circumvent this drawback, photocatalytic-mediated type I PDT processes hold great interest due to their reduced dependence on oxygen levels in comparison to type II PDT pathways, as the initial steps of several type I-related photocatalytic mechanisms can occur without molecular oxygen, and cell enzymes can also catalyze the disproportionation of certain originated ROS, consequently reverting these species into de novo available ground-state oxygen molecules.^19,21–23^

On a structural basis, benzazoles represent privileged scaffolds for the construction of enhanced iridium­(III) PSs.^15,24–28^ The introduction of benzazoles fosters favored triplet metal-to-ligand charge transfer (^3^MLCT) transitions for a boosted photodynamic activity. Heterocyclic azole rings within the ancillary ligand exert significant electron-withdrawing effects, thus exhibiting propensity to accept electron density and leading to a substantial stabilization of LUMO-contributing orbitals.^29^ Additionally, many benzazoles exhibit notable biological activity, rendering benzazole-based iridium­(III) complexes as promising inhibitors of essential metabolites for cancer survival and modulators of genes involved in evading cell death.^30,31^

In line with these trends, the strategic and collective incorporation of electron-donating 2-thienyl moieties, fluorinated substituents, and/or ester groups was found very recently to render two cyclometalated iridium­(III) complexes (**IrA**-**IrB**, Chart 1) with a strong photodynamic action. The resulting nanoparticles exhibited an elevated photobiological activity (PI = 34–89) in colon cancer cells (CT-26) under blue light irradiation (450 nm, 1.2 J/cm^2^), generating singlet oxygen and ROS species through combined Type I/II mechanisms.^32–34^

**Chart 1:**
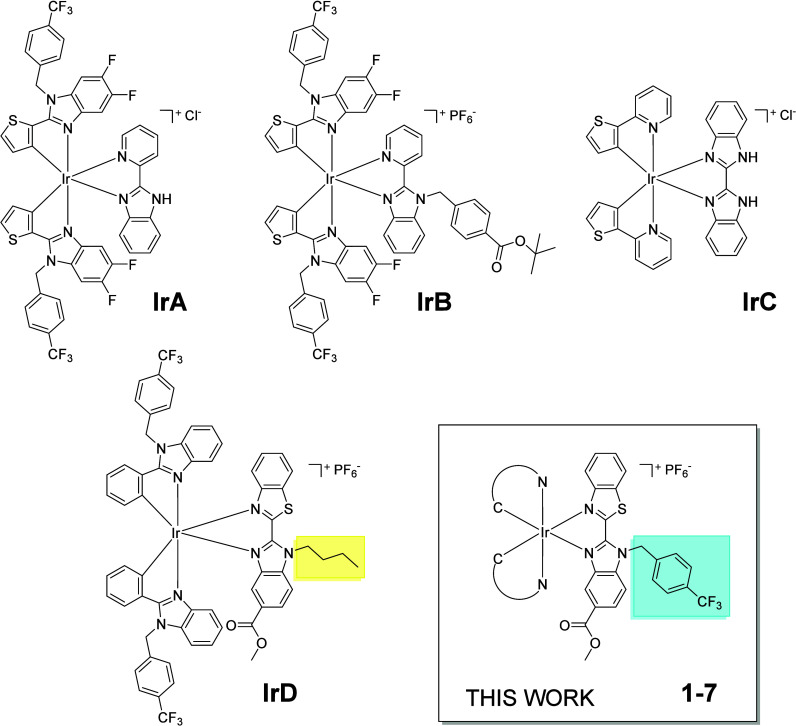
Some Representative Benzimidazole-Based Iridium­(III) Photosensitizers of General Formula [Ir­(C^N)_2_(N^N)]^+^, Along with the General Structure of Synthesized Complexes **1–7**

Notably, the fusion of two benzazole cores was proved crucial in the construction of Ir­(III) complexes with an improved photocatalytic activity, as their more electron-acceptor nature facilitated enhanced ^3^MLCT transitions, ultimately leading to a highly reactive photodynamic response.^29^ Specifically, complex **IrC** (Chart 1), featuring bisbenzimidazole as the N^N ligand, is an efficient PS in 3D melanoma spheroids, with repeated short-time irradiation causing cumulative killing.^35^

The research group of J. Ruiz designed an iridium­(III) complex with an ester-functionalized 2-(2′-benzothiazolyl)­benzimidazole (bzt-bu-CO_2_Me-bzim) ligand (**IrD**, Chart 1). The appended lipophilic butyl group in the ancillary ligand rendered an exceptional cytotoxic activity against ovarian and breast cancer cells already in the dark.^36^

In order to overcome the cytotoxic activity in the dark and achieve a strong phototherapeutic effect, this work has focused on changing the lipophilic butyl group by a (trifluoromethyl)­benzyl moiety. These scaffolds have been found to boost the intracellular photogeneration of type I ROS species, thereby potentiating the photoactivity of iridium­(III) complexes and enabling the exploitation of type I-guided mechanisms with a potential application in hypoxic contexts.^27,37^

We herein report on the chemical, photophysical, and photocatalytic characterization, including stability and aggregation, of a new class of novel Ir­(III)-bzt-tfb-CO_2_Me-bzim photosensitizers (complexes **1–7**, Chart 1). Additionally, we also conducted their biological evaluation in 2D and 3D cancer cell models, including antiproliferative effects, intracellular accumulation and localization, in-cell production of ROS and oxidation of NADH. Thus, this study presents some examples of biscyclometalated iridium­(III) photosensitizers as promising candidates in the treatment of hypoxia-led resistant cancers.

## Results and Discussion

### Synthesis and Characterization

An overview of the chemical structures of the proligands, the ancillary ligand and the iridium­(III) complexes, alongside their synthetic route, can be found in Scheme 1. The proligands were purchased from commercial sources, except for **HC^N6** and **HC^N7**, which were obtained after a substitution reaction between 4-(trifluoromethyl)­benzyl bromide and the correspondent arylbenzimidazole (Figures S1 and S2 for their ^1^H NMR spectra).^37^ The ancillary ligand (**L**) was prepared in a three-step synthetic pathway that involves the amination reaction between 4-chloro-3-nitrobenzoate and 4-(trifluoromethyl)­benzylamine to form intermediate **A**, the Fe-catalyzed reduction of the nitro derivative **A** to form the intermediate amine **B** and the latter sodium bisulfite-mediated condensation of **B** with benzo­[d]­thiazole-2-carbaldehyde. These synthetic steps were performed by following protocols previously reported in the literature.^38–40^ The ancillary ligand **L** (bzt-tfb-CO_2_Me-bzim) was fully characterized by ^1^H-, ^13^C-, and ^19^F­{^1^H}-NMR spectroscopy (Figures S3–S5) and its chemical identity and purity were assessed by HR-ESI-MS spectrometry (Figure S6).

**Scheme 1:**
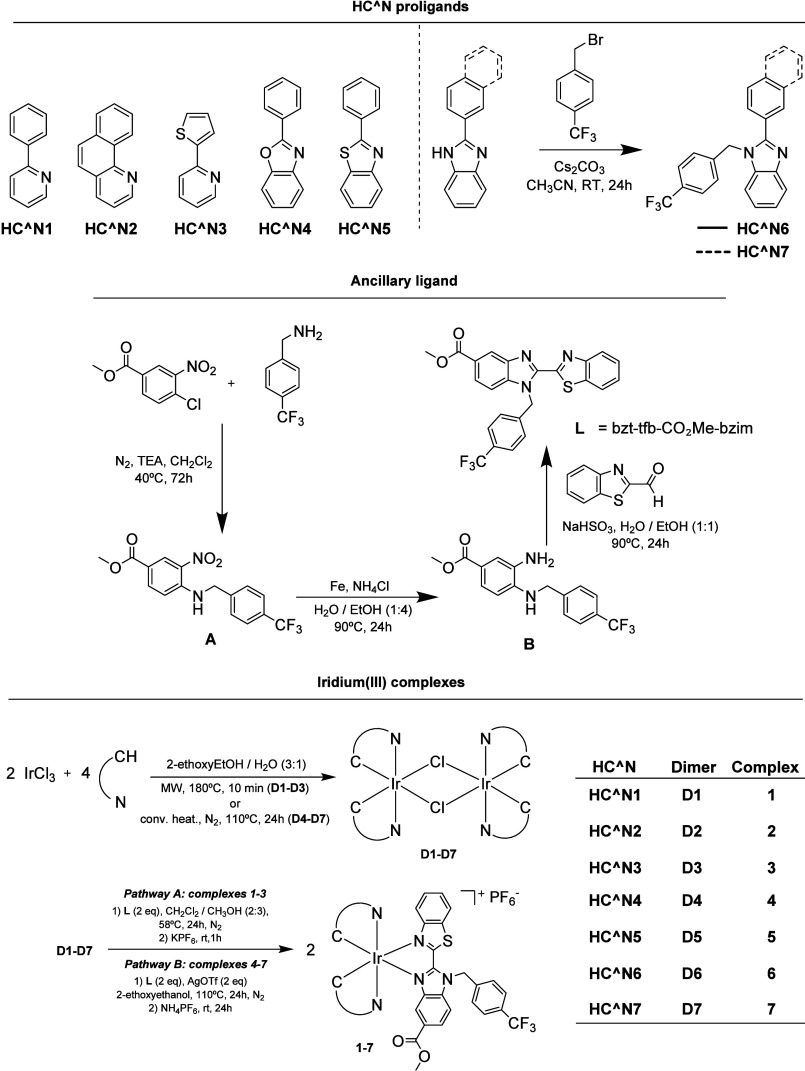
Overview of the Synthesis and Chemical Structures of the HC^N Proligands (**HC^N** **1–** **HC^N7**), N^N Ancillary Ligand (bzt-tfb-CO_2_Me-bzim), [Ir­(C^N)_2_(μ-Cl)]_2_ Dimers (**D1–D7**) and the Monomeric Ir­(III) Complexes (**1**–**7**)

The iridium­(III) complexes were obtained according to previous synthetic reports.^15,36,41–43^ Briefly, iridium trichloride (IrCl_3_) and the corresponding HC^N proligand were mixed in a 2-ethoxyethanol/water (3:1) mixture and heated at 180 °C in a microwave reactor for 10 min (**D1**–**D3**) or at reflux for 24 h via conventional heating (**D4**–**D7**). For the preparation of complexes **1–3**, the iridium dimers (**D1**–**D3**) and N^N ancillary ligand **L** were mixed in a dichloromethane/methanol (2:3) mixture and heated at reflux for 24 h. The preparation of complexes **4**–**7** required harsher conditions, as dimers **D4**–**D7**, the ancillary ligand and silver triflate were mixed in 2-ethoxyethanol and stirred at 110 °C in the dark for 24 h. In both synthetic pathways, the last step involved the substitution of the counteranion to PF_6_^–^, and final complexes **1**–**7** were obtained as orange to dark red (view Figure S7) hexafluorophosphate salts after purification by column chromatography.

All complexes were fully characterized by ^1^H-, ^13^C-, ^19^F­{^1^H}-, and ^31^P-NMR spectroscopy (Figures S8–S35). Their ^1^H-NMR spectra (Figure 1) display a characteristic aliphatic signal around 3.0 ppm with a singlet multiplicity, attributable to the methyl group in the ester functionality from the ancillary ligand.^44^ Interestingly, this singlet is notably shifted to higher field in complexes **6**–**7**, suggesting the orientation of the ester group toward the shielding aromatic rings of the benzimidazole-derived cyclometalated ligands.^36,45^ Further signals in the range of 5.0–9.0 ppm comprise aromatic protons and aliphatic −CH_2_– methylene groups. The ^19^F­{^1^H}-NMR spectra depict the characteristic singlet peaks arising from the −CF_3_ groups in both the ancillary and cyclometalated ligands, and doublet peaks corresponding to the PF_6_^–^ counteranion molecules. The ^31^P-NMR spectra also confirmed the presence of the PF_6_^–^ counteranion, with a septuplet multiplicity.

**Figure 1:**
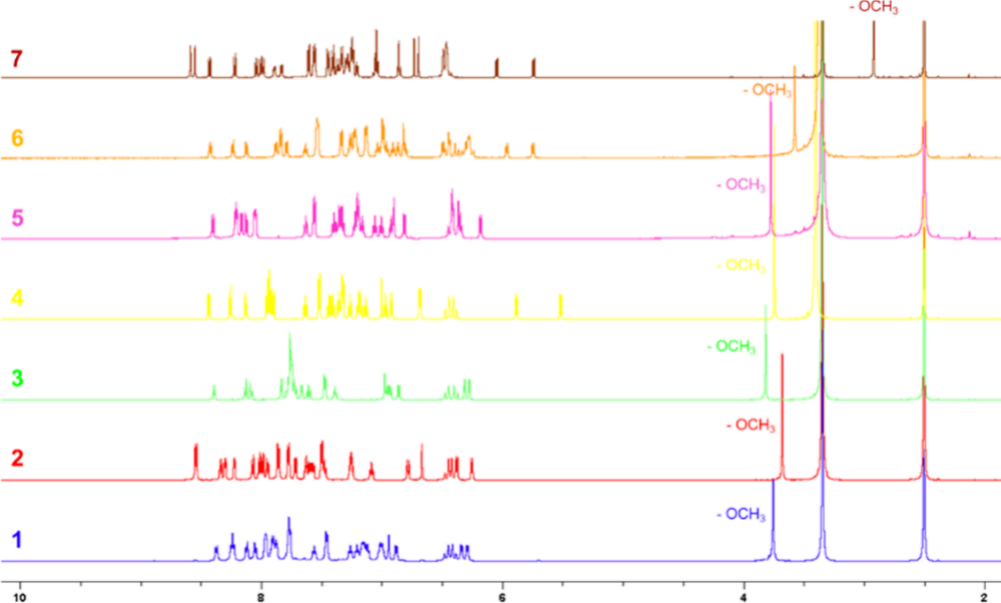
Comparison of the ^1^H-NMR spectra of complexes **1–7** (DMSO-*d*_6_, 600 MHz).

The chemical identity of **1–7** was further confirmed by HR-ESI-MS spectrometry (Figure S36), and the purity, which was found to be higher than 95% in all cases, was assessed by elemental analysis and high-performance liquid chromatography (HPLC) (Figure S37).

### X-ray Diffraction Studies

Single crystals of complexes **2** and **5** suitable for X-ray diffraction analysis were obtained by slow evaporation of dichloromethane/hexane solutions of the complexes at rt for 3 days (Figure 2). Crystallographic data is collected in Tables S1–S9. The iridium­(III) complexes adopted a slightly distorted octahedral geometry around the iridium center, with the nitrogen atoms from the C^N ligands (N4, N5) being disposed in *trans* orientation to each other, while the nitrogen atoms from the ancillary ligand (N1, N2) are facing the carbon atoms from the cyclometalated ligands in *trans* position. The *trans* influence exerted by σ-donor carbon atoms from the C^N ligand was evidenced in the longer Ir–N1 and Ir–N2 distances.^12,15^ Further analysis revealed that the crystal packing of complexes **2** and **5** encompassed π···π, C–H···π and C–H···F interactions (Figures S38–S40). While stacking π···π interactions were established between freely rotating (trifluoromethyl)­benzyl groups of the N^N ligand and the C^N benzo­[h]­quinoline ligands, forming supramolecular triads in complex **2**, complex **5** was found to organize in dimeric species by the stacking interactions between the benzothiazole moieties. Conversely, C–H···F interactions were detected between PF_6_^–^ counteranion molecules and complex **2**.

**Figure 2:**
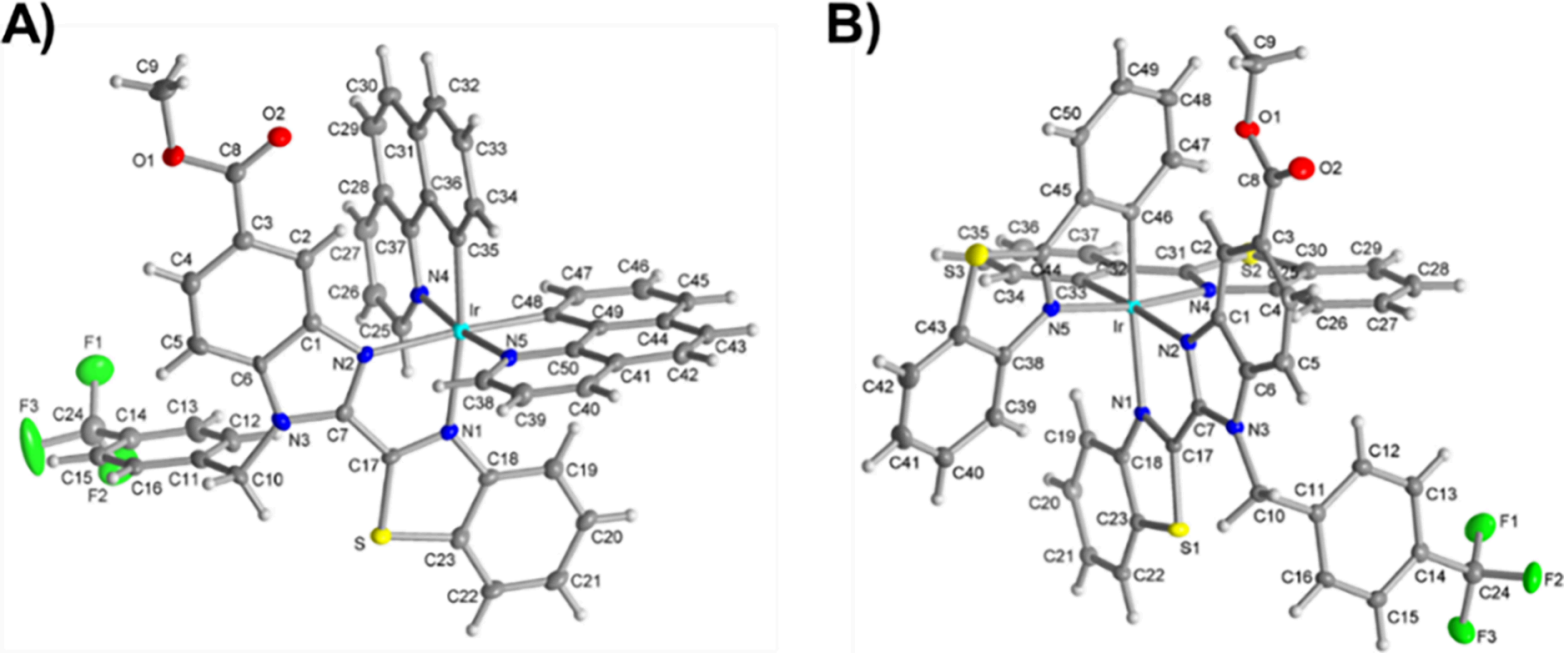
Refined crystal structures of complexes **2** (A) and **5** (B) (CCDCs: 2498248 and 2498249). Ellipsoids are drawn at a 50% level of probability. Hexafluorophosphate counteranion and cocrystallizing dichloromethane molecules have been omitted for clarity.

### Stability and Aggregation in Culture Medium

Prior to conducting any biological studies, the stability of complexes **1–7** was first investigated in DMSO by UV/vis absorption spectroscopy. Solutions of the complexes (10 μM) were prepared and their absorption profiles were monitored at t = 0 and after 48 h of incubation at room temperature in the dark. As exemplified for complex **7** (Figure 3A, view Figure S41 for the rest of complexes), no noticeable changes were observed after 48 h of incubation, suggesting the stability in DMSO of the iridium complexes.

**Figure 3:**
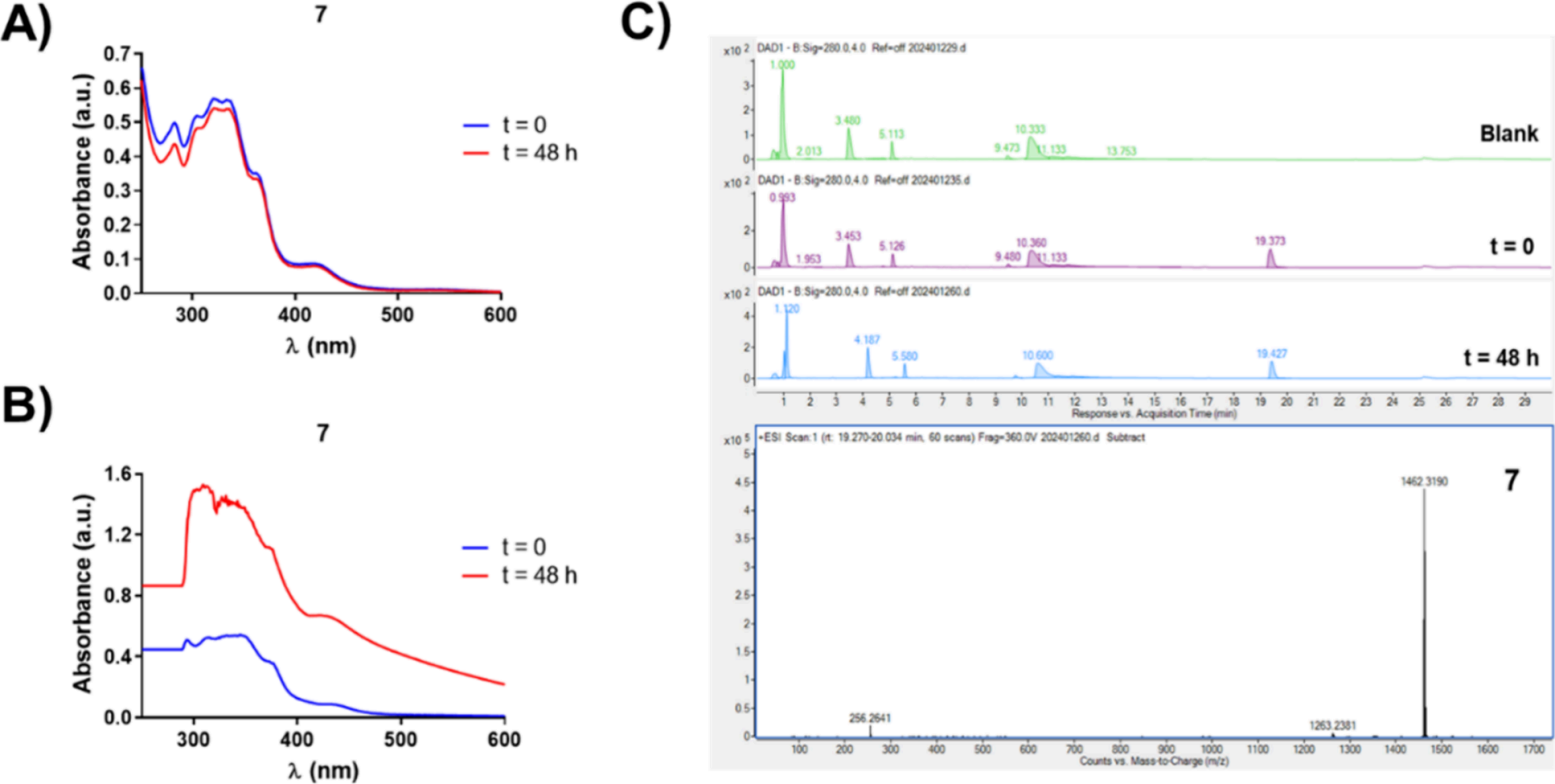
Chemical stability of complex **7** (10 μM) in DMSO assessed by UV/vis absorption spectroscopy (A). Monitoring of the UV/vis absorption spectrum of complex **7** (10 μM) in a DMEM (+10% FBS)/DMSO (95:5) mixture before (*t* = 0) and after (*t* = 48 h) incubation at 37 °C (B). Stability of complex **7** (10 μM) in a DMEM (+10% FBS)/DMSO (95:5) mixture recorded by HPLC/HR-ESI-MS (C).

Followingly, the stability of **1–7** was evaluated under physiological conditions to mimic the cellular environment. The UV/vis absorption profiles of these complexes in a DMEM (+10% FBS)/DMSO (95:5) mixture were monitored at *t* = 0 and after 48 h of incubation at 37 °C. Serum proteins have been found to play a pivotal role in the stabilization and self-assembly of metal complexes.^46,47^ As observed in Figure S42, no evident changes were detected for complexes **1**–**5**. Strikingly, benzimidazole-derived complexes **6**–**7** underwent a significant hyperchromic effect with time (Figures 3B and S42), possibly attributed to an aggregation process.^46,48^ Interestingly, turbidity phenomena could be observed in the vials, but very distant from the characteristic precipitation of nonsoluble complexes (Figure S43). As a complementary approach, HPLC/HR-ESI-MS measurements were conducted to confirm the stability of the Ir­(III) complexes in culture medium and corroborate that the changes observed in UV/vis absorption spectroscopy were not associated with any undesired side reaction (Figures 3C and S44–S49).

Aggregation has extensively been proven to play a pivotal role in defining the biological properties of a drug, regulating its mechanisms of cellular accumulation.^46,48,49^ Ir­(III) complexes tend to aggregate into stacks in water due to their relatively high lipophilicity.^18,50^ The formed aggregates were characterized by Tyndall effect (Figure S50) and nanoparticle tracking analysis (NTA) (Table S10). NTA is an alternative methodology to DLS. It enables the visualization and analysis of individual nanoparticles in real time, providing valuable information regarding the size distribution and concentration of particles in solution, thus giving substantial insights into the self-assembly process.^51^ As observed, all complexes, except for **5**, were able to form nanoparticles with average diameters around 200 nm right after solution, proving to have an optimal size for their cellular accumulation.

### Photophysical Characterization

The photophysical properties of **1–7** were studied by UV/vis absorption and emission spectroscopy. The UV/vis absorption spectra of the iridium­(III) complexes were registered in acetonitrile (Figure S51) and in water (1% DMSO, Figure 4A). As observed, the absorption profiles revealed bands with high absorbance intensities in the range of 260–340 nm, attributable to ligand-centered (LC) transitions. Next to these bands, less intense absorption bands could be found in the range between 350 and 380 nm, possibly comprising a mixture of ligand-to-ligand (LLCT) and spin-allowed metal-to-ligand (^1^MLCT) charge transfer processes. The broad and low intensity bands around 410–440 nm could predominantly arise from spin-forbidden ^3^MLCT processes originated from triplet excited state population via ISC.^52,53^

**Figure 4:**
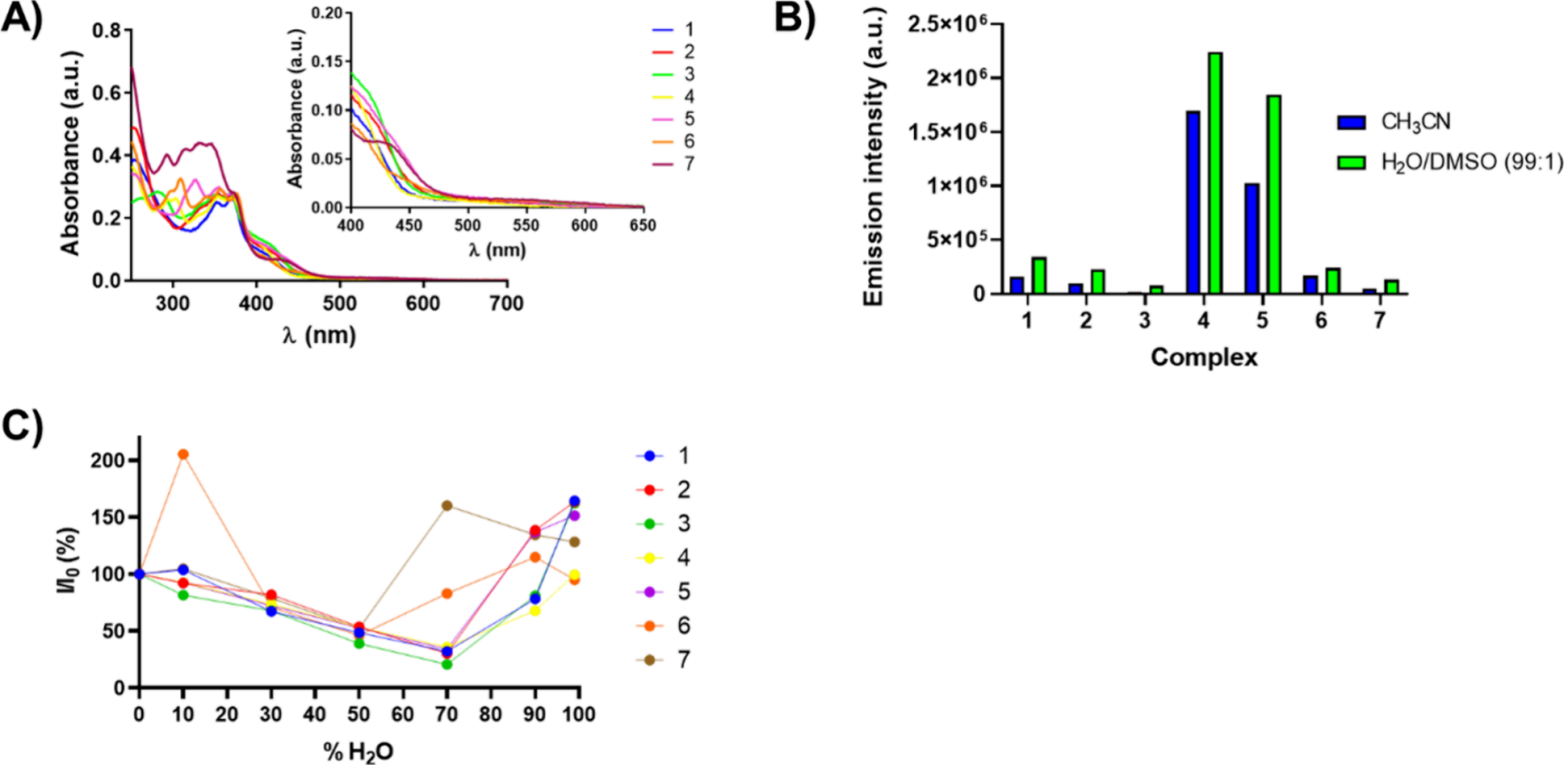
UV/vis absorption spectra of complexes **1–7** (10 μM) in a H_2_O/DMSO (99:1) mixture (A). Emission intensity (λ_exc_ = 420 nm) of complexes **1–7** (10 μM) at their maxima recorded in acetonitrile and a H_2_O/DMSO (99:1) mixture (B). Changes in the fluorescent intensity (λ_exc_ = 420 nm) of complexes **1–7** (10 μM) in different H_2_O/DMSO mixtures while increasing the volumetric water fraction (C).

The emission spectra of **1**–**7** were also recorded in aerated acetonitrile (Figure S52) and water (1% DMSO, Figure S53) solutions. All complexes displayed a wide emissive character, exhibiting emission bands in the red region of the visible spectrum (610–680 nm). Among the iridium­(III) complexes, benzoxazole (**4**) and benzothiazole (**5**) derivatives exhibited the strongest luminescence intensities (Figure 4B). Additionally, the emission bands of these complexes were found to be blue-shifted in comparison to the rest of complexes. This finding suggests the MLCT-nature of the emission band, as the greatest electron-withdrawing character of the benzoxazole and benzothiazole cores in the cyclometalated ligand could enlarge the HOMO–LUMO gap, comprised by MLCT (d_M_ → π*_N^N_) transitions.^54^

Most notably, an enhancement in the emission intensity of complexes **1**, **2**, and **6** in water compared to acetonitrile was observed. It has been extensively reported that the increasing presence of water can significantly modify the emissive behavior of transition metal complexes through aggregation- and polarity-related processes.^15,50,55^ Considering this tendency, it was decided to monitor their emission spectra upon continuous increment of the volumetric water fraction in different H_2_O/DMSO mixtures (Figures 4C and S54). As observed, two major trends were noticed: a progressive decrease in the luminescence intensity up to 50% v/v (complexes **6–7**) or 70% v/v (complexes **1–5**) water fraction, followed by a notable increase up to 99% v/v water fraction. This dual behavior can likely be attributed to a competition between aggregation-caused quenching (ACQ) and aggregation-induced emission (AIE) processes.^56^ ACQ is often related to a twisted intramolecular charge transfer (TICT) phenomenon. Upon water presence, electronically excited complexes may adopt twisted geometries that favor ligand-to-ligand intramolecular deactivation in detriment of MLCT transitions, thereby hindering their MLCT-emissive character. Interestingly, a correlation between the more pronounced AIE effect of benzimidazole-based complexes **6–7** and the strong turbidity and Tyndall effect appreciated in their aqueous solutions could be established. These evidence highlight the contribution of aryl rings within the benzimidazole moieties in the robust self-assembly of complexes **6–7**.

Emission quantum yields were measured for complexes **1–7**. 2-Phenylbenzoxazole- (**4**) and 2-phenylbenzothiazole-based (**5**) complexes showed the highest photoluminescence, with emission quantum yield values of 13.5 and 9.6%, respectively. The emission for all the iridium­(III) complexes exhibited a biexponential decay, comprising short-lived components (30–60 ns) with a minor contribution, and predominant long-lived components (80–300 ns) that governed the emission profile of these complexes. Strikingly, complexes **4** and **5** were again found to have the longest triplet lifetimes. The relative stability of the triplet excited states suggests the potential photodynamic activity of these complexes. All the photophysical data can be found in Table S11.

### Photochemical Stability

Prior to evaluating the photochemical behavior of complexes **1**–**7**, their stability upon light irradiation was assessed by UV/vis absorption spectroscopy. The absorption profiles of solutions of the iridium­(III) complexes (10 μM) in DMSO did not show significant changes after 1 h irradiation with a blue lamp, thereby suggesting their photochemical stability under these conditions (Figure S55).

### Photocatalytic Singlet Oxygen Production

The photocatalytic ability of complexes **1**–**7** to confer the activation energy to ground-state oxygen and subsequently produce singlet oxygen upon light irradiation was assessed in cell-free media by an indirect spectroscopic method.^15^ The absorbance of 1,3-diphenylisobenzofuran (DPBF, 50 μM, λ_abs_ = 411 nm) in acetonitrile was monitored upon incubation with complexes **1**–**7** (30–50 μM) and exposure to short (15 s) irradiation intervals with a blue lamp (λ_irrad_ = 465 nm, 0.4 mW/cm^2^). To quantify the singlet oxygen production, singlet oxygen quantum yields were calculated using [Ru­(2,2′-bipyridine)_3_]^2+^ (5 μM) as a reference (Φ_Δ_ = 0.56 in acetonitrile) and following eq 1. As observed in Figures 5A and S56, all complexes caused a decrease in the absorbance of DPBF and exhibited moderate to very high quantum yields.

**Figure 5:**
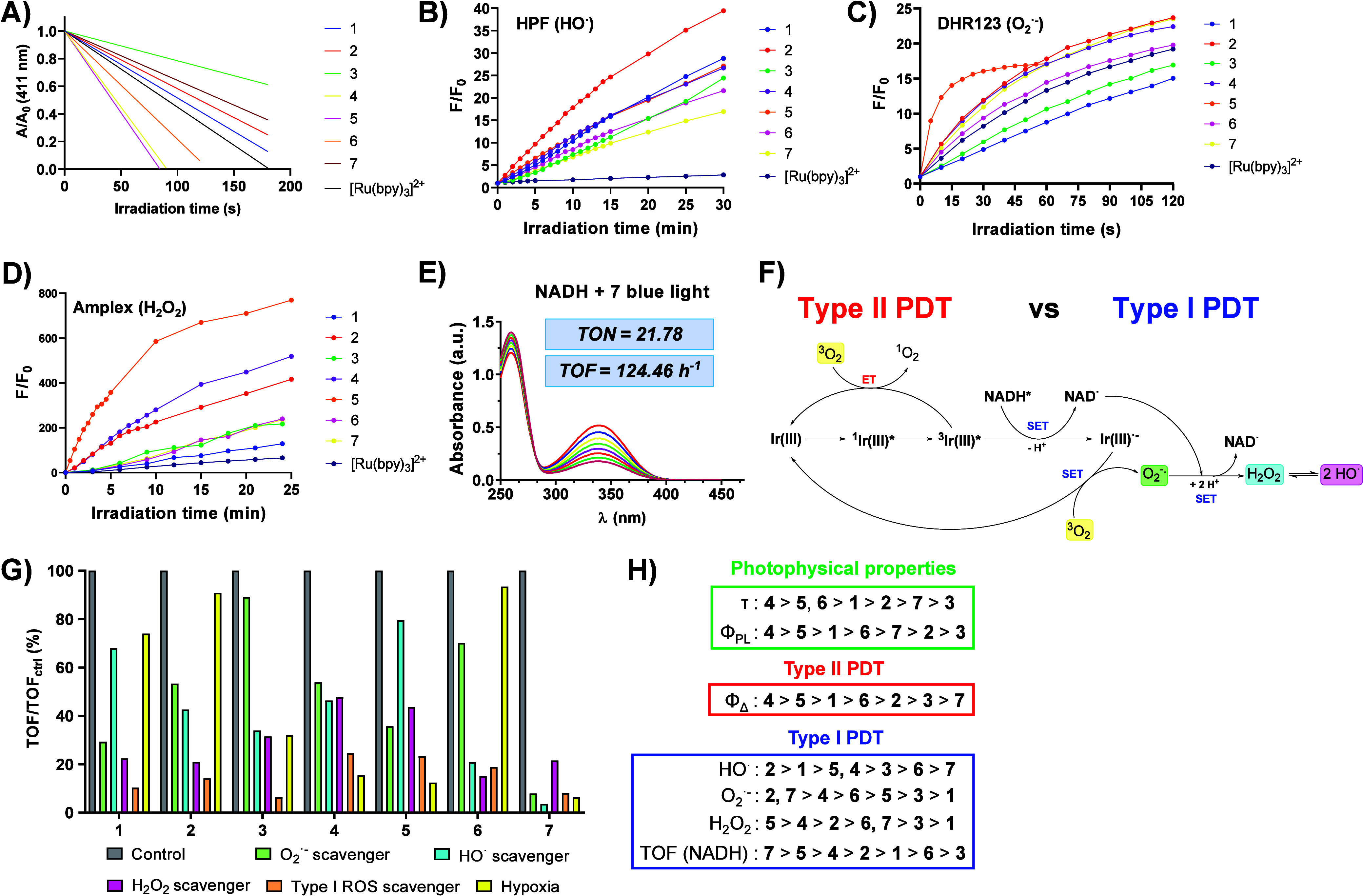
A/A_0_ (DPBF, 411 nm, acetonitrile, 50 μM) versus irradiation time (s) plot obtained after incubation with complexes **1–7** (30–50 μM) or reference [Ru­(2,2′-bipyridine)_3_]^2+^ (5 μM) and exposed to blue (λ_irrad_ = 465 nm, 0.4 mW/cm^2^) light irradiation (A). Increase in the fluorescent signal (F/F_0_) of HPF (10 μM) (B), DHR123 (10 μM) (C) and Amplex Red (10 μM) (D) probes upon incubation with complexes **1–7** (10 μM for the HPF and DHR123 assays, 5 μM for the Amplex Red assay) in H_2_O (5% DMF) and exposure to blue (λ_irrad_ = 465 nm, 5 mW/cm^2^) light irradiation at short irradiation intervals. Monitoring of the absorption profile of NADH (100 μM) upon incubation with complex **7** (2.5 μM) in a H_2_O/DMF (95:5) mixture and exposure to blue (λ_irrad_ = 465 nm, 5 mW/cm^2^) light irradiation at short irradiation intervals (E). Overview of the catalytic mechanism for the photoinduced oxidation of NADH (F). Turnover frequency values for the photooxidation of NADH induced by complexes **1–7** (5 μM) upon coincubation with tiron (O_2_^.–^ scavenger), mannitol (HO^·^ scavenger), sodium pyruvate (H_2_O_2_ scavenger) or trolox (general type I ROS scavenger), or at inert atmosphere (hypoxic conditions), and blue (λ_irrad_ = 465 nm, 5 mW/cm^2^) light irradiation (G). Comparison of the experimental trends retrieved during the photophysical and photochemical assessment of complexes **1–7** (H).

Most strikingly, complexes **4** and **5** showed the highest quantum yield values among iridium­(III) complexes (0.74 and 0.72, respectively). In this regard, it is noteworthy to mention that very similar trends can be retrieved from the singlet oxygen (**4** > **5** > **1** > **6** > **2** > **3** > **7**) and emission (**4** > **5** > **1** > **6** > **7** > **2** > **3**) quantum yield values and emission lifetimes (**4** > **5**,**6** > **1** > **2** > **7** > **3**) from the triplet excited states, supporting the fact that longer-lived triplet states lead to a phosphorescent-like emission and effective energy transfer processes to ground-state oxygen.

### Type I ROS Determination and Photocatalytic Oxidation of NADH

The photochemical evaluation of complexes **1–7** as type I photosensitizers began with the quantitative detection of HO^·^, O_2_^·‑^, and H_2_O_2_ species, using the corresponding fluorogenic probes (Figures 5B-D and Figures S57–S59). As observed, the incubation of hydroxyphenylfluorescein (HPF, HO^·^ probe), dyhidrorhodamine 123 (DHR123, O_2_^·‑^ probe) and Amplex Red (H_2_O_2_ probe) with complexes **1–7** and exposure to short irradiation intervals led to a progressive increase of the fluorescence signal arising from the probes, thereby evidencing the photogeneration of these ROS species.

Thereafter, the qualitative determination of the photoinduced damage caused in essential biomolecules for cancer cell survival and the subsequent formation of hydrogen peroxide was studied.^19,57–59^ Solutions of NADH, 9-ethylguanine, glutathione, ascorbic acid, cysteine and histidine (1 mM) in a H_2_O/DMF (95:5) mixture were incubated with complexes **1–7** (10 μM) and irradiated for 1 h. Hydrogen peroxide strips were dipped into the solutions and the photo-oxidant activity of the iridium complexes was assessed by the strip color scale. As observed in Figure S60, all complexes were found to oxidize NADH upon light irradiation as the strips got heavily colored. Additionally, complexes **2** and **4** showed a significant photooxidant activity against Cys, while complex **5** further proved to induce the photooxidation of GSH and HAsc.

Nicotinamide adenine dinucleotide hydrogen (NADH) is a key coenzyme involved in maintaining cellular redox balance and energy metabolism through the electron transport chain (ETC), the tricarboxylic acid (TCA) cycle, and glycolysis. Disruptions in the NADH levels lead to an impairment of the ETC, therefore depriving cancer cells from the energy required for their proliferation.^57,60,61^ To gain deeper knowledge about NADH photooxidation, its absorption profile (100 μM) was monitored upon incubation with **1–7** (5 μM) in a H_2_O/DMF (95:5) mixture and subjected to short (5 min) irradiation intervals with a blue lamp. As observed in Figures 5E and S61, there was a progressive decrease in NADH absorbance as the solutions were continuously being irradiated. Notably, no evident changes were observed when NADH was incubated with the iridium­(III) complexes in the dark (Figure S62). The calculation of the turnover frequency (TOF) using eqs 2 **-** [Disp-formula eq3] revealed the highest value (TOF = 124.46 h^–1^) for complex **7**. Singlet oxygen quantum yields and TOF values are collected in Table S12.

Mechanistically, upon light absorption, the Ir­(III) photosensitizer transitions to an excited state and accepts an electron from NADH, generating the NAD·^+^ radical and a reduced Ir­(II) species (Ir^3+^* + NADH → Ir^2+^ + NAD^·+^). The NAD^·+^ radical rapidly converts to NAD^+^, while Ir^2+^ is reoxidized by a sacrificial electron acceptor, such as molecular oxygen, completing the catalytic cycle (Ir^2+^ + O_2_ → Ir^3+^ + O_2_^.–^) (view Figure 5F). This enables a catalytic amount of the Ir complex to oxidize a substantial quantity of NADH.^61^ To corroborate the catalytic nature of NADH photooxidation and ascertain the leveraged ROS species upon light irradiation, the NADH assay was repeated in the presence of tiron (O_2_^.–^ scavenger), mannitol (HO^·^ scavenger), sodium pyruvate (H_2_O_2_ scavenger) or trolox (general type I ROS scavenger), or under inert atmosphere, mimicking hypoxic conditions (Figures S63–S67). TOF values were calculated and normalized to the control value (no scavengers and normoxic conditions). As depicted in Figure 5G, NADH photooxidation was overall inhibited in the presence of the scavengers. This effect was emphasized for complex **7**, the complex with the highest TOF value among the iridium complexes. Interestingly, the catalytic ability of complexes **1**, **2** and **6** to photooxidize NADH was barely hindered in the absence of oxygen, outscoring their suitability in photodynamic therapy under hypoxic conditions.

Figure 5H collects all the experimental trends retrieved in the photophysical and photochemical evaluation of complexes **1–7**. Complex **4** stands out among the iridium compounds for its superior ability to photogenerate singlet oxygen, accompanied by the relatively high rates of type I ROS production and NADH oxidation, and the longest-lived triplet states. These evidence postulate complex **4** as the most promising candidate for the phototherapeutic treatment of cancers via type I and type II mechanisms.

### Antiproliferative Activity

This study was primarily initiated with the intention to identify effective and selective therapeutic agents for lung carcinoma, a leading cause of cancer-related mortality worldwide that is often associated with poor clinical outcomes. Therapy resistance, disease relapse, and late-stage diagnosis frequently render tumors inoperable, shifting treatment strategies from curative to palliative care. These challenges underscore the pressing need for innovative therapeutic approaches. We investigated a series of iridium­(III)-benzothiazolylbenzimidazole complexes, initially screening their antiproliferative activity and phototoxic potential to identify the most promising candidates. Cellular models were selected based on epidermal growth factor receptor (EGFR) status to capture clinically relevant contexts. A549 cells, which have wild-type EGFR, provided a baseline for assessing photodynamic efficacy independent of EGFR mutations.^62^ In contrast, HCC827 cells harbor an exon 19 deletion in EGFR, a mutation common in nonsmall cell lung cancer (NSCLC) that confers sensitivity to tyrosine kinase inhibitors but may also promote resistance. Including HCC827 thus allowed assessment of activity in a therapeutically challenging subtype.^63^ To broaden applicability, HCT-116 colorectal carcinoma cells were also incorporated, representing a distinct tissue type amenable to light irradiation.^64^ Furthermore, the nonmalignant human lung fibroblast line MRC-5 was included as a control to evaluate the selectivity of the complexes and calculate the therapeutic index (TI) under dark conditions.

Table 1 summarizes the photoactivation profiles of seven Ir­(III) complexes across these models. Complex **4** exhibited exceptional potency, achieving nanomolar activity after irradiation (IC_50_ = 12 nM in A549; PI = 310) and strong effects in HCC827 (PI = 35.3) and HCT-116 (PI = 41). While most complexes showed TI values ranging from 0.4 to 1.5, suggesting comparable dark toxicity between malignant and nonmalignant lines, their therapeutic potential was revealed upon irradiation. Complex **7** demonstrated broad-spectrum photoactivity with high phototoxic indices (PI = 71 in HCT-116; PI = 38.6 in HCC827) and minimal dark toxicity (IC_50_ ≥ 200 μM). Complex **5** also showed strong photoactivation (PI = 84 in A549; PI = 75 in HCT-116). In contrast, complex **6** exhibited negligible photoactivity, suggesting limited therapeutic potential. Overall, most complexes exhibited reduced IC_50_ values upon irradiation, confirming their potential as photoactivatable anticancer agents.

**Table 1:** Antiproliferative Activity (IC_50_ values^a^) of the Investigated Complexes Determined in Lung Carcinoma and Colorectal Carcinoma Cells^b^ ^,^ ^c^

	**A549**			**HCC827-Gas-Luc2**			**HCT-116**			**MRC-5**	
	**Dark**	**Irr.**	**PI** ^d^	**Dark**	**Irr.**	**PI** ^d^	**Dark**	**Irr.**	**PI** ^d^	**Dark**	**TI** ^e^
**1**	10 ± 1	0.23 ± 0.02	43	0.99 ± 0.06	0.035 ± 0.001	28.1	4.5 ± 0.2	0.19 ± 0.03	24	8 ± 2	1.5
**2**	22 ± 3	0.54 ± 0.08	41	2.2 ± 0.2	0.10 ± 0.03	22.1	6.1 ± 0.7	0.48 ± 0.02	13	15 ± 2	1.5
**3**	16 ± 2	0.47 ± 0.03	34	1.34 ± 0.02	0.098 ± 0.003	13.6	4.9 ± 0.3	0.44 ± 0.05	11	6 ± 2	0.8
**4**	3.1 ± 0.2	0.012 ± 0.002	310	1.29 ± 0.05	0.036 ± 0.003	35.3	3.7 ± 0.4	0.09 ± 0.01	41	4.1 ± 0.4	1.5
**5**	8.4 ± 0.7	0.14 ± 0.02	84	1.67 ± 0.02	0.06 ± 0.02	28.4	8.3 ± 0.6	0.11 ± 0.02	75	4.8 ± 1.0	0.8
**6**	53 ± 4	41 ± 3	1.3	12.9 ± 0.9	4.2 ± 0.8	3.0	32 ± 2	2.7 ± 0.3	12	13 ± 2	0.4
**7**	≥200	5.2 ± 0.4	≥38	≥200	5.18 ± 0.04	≥38.6	≥200	2.8 ± 0.2	71	≥200	nd

a Concentration that causes 50% inhibition of cell proliferation expressed in μM.

b Cells were treated for 60 min with increasing concentrations of the investigated complexes, followed by 60 min of irradiation with blue light (420 nm; 58 W/m^2^) and 70 h of drug-free incubation.

c IC_50_ values were determined by MTT assay.

d PI—phototoxicity index was calculated as the ratio of IC_50_ determined under the dark conditions/IC_50_ determined for 420 nm irradiated samples.

e TI—Therapeutic Index; the ratio of IC_50_ (nonmalignant MRC-5) to mean IC_50_ (malignant).

As aforementioned, the clinical challenge of treating solid tumors is often exacerbated by the hypoxic microenvironment, which typically renders traditional oxygen-dependent (^1^O_2_-mediated) therapies ineffective. Consequently, we investigated the activity of these complexes under hypoxic conditions (1% O_2_) using the A549 model (Table S13). While a decrease in photopotency was observed across the series compared to normoxic conditions—indicative of a partially oxygen-dependent mechanism—several complexes maintained cytotoxic photoactivity. Notably, complex **4** remained highly effective under hypoxia with a submicromolar IC_50_ of 0.37 μM and a PI of 23.5. This resilience in low-oxygen environments suggests that these Ir­(III) complexes may operate via a Type I photochemical pathway (radical formation) or through direct, photoinduced molecular interactions that do not strictly require molecular oxygen.

### Cellular Accumulation

Cellular accumulation is a key determinant of a compound’s pharmacological activity, therapeutic efficacy, and potential toxicity.^64,65^ The intracellular levels of iridium in A549 and HCC827 cells were quantified by ICP-MS after 24 h exposure to the tested complexes (2 μM) (Table 2). Uptake varied between the two cell lines. In A549 cells, accumulation ranged from 237 to 961 ng Ir per million cells, with complex **7** showing the highest uptake. In HCC827 cells, uptake ranged from 415 to 924 ng Ir per million cells, with complexes **1**, **3**, and **4** displaying the highest accumulation. Notably, complex **1** accumulated more than 2.5-fold higher in HCC827 than in A549 cells, suggesting preferential uptake or more efficient internalization in this cell line. Complex **3** showed a similar trend, with more than double the accumulation in HCC827.

**Table 2:** Cellular Uptake of Complexes **1**–**7** in A549 and HCC827 Cells (Expressed as ng Ir/10^6^ Cells)^a^

Complex	**A549**	**HCC827-Gas-Luc2**
**1**	237 ± 2	613 ± 42
**2**	343 ± 41	475 ± 13
**3**	305 ± 2	657 ± 34
**4**	421 ± 74	618 ± 77
**5**	250 ± 22	598 ± 43
**6**	321 ± 35	415 ± 75
**7**	961 ± 45	924 ± 77

a Cells were treated for 24 h with 2 μM of the tested complexes. Data are presented as means ± SDs from two independent experiments, as determined by ICP-MS.

These differences may reflect the distinct EGFR statuses of A549 (wild-type) and HCC827 (exon 19 deletion).^66^ If iridium complexes are internalized via receptor-mediated endocytosis,^15^ EGFR mutations could influence receptor availability or endocytic pathways, thereby modulating uptake.^66–69^ Further investigation is required to define the molecular links between EGFR signaling and iridium uptake. Higher accumulation in HCC827 cells, particularly with complexes **1**, **3**, and **5**, correlated with enhanced cytotoxicity compared to A549 cells, especially under light activation. In dark conditions, increased uptake also generally aligned with greater cytotoxicity in HCC827 cells (except for complex **7**), whereas this correlation was less consistent in A549 cells.

Cellular accumulation can be attributed to the lipophilicity of the complex and the size of the resulting nanoparticles in aqueous solution. The experimental trends for cellular accumulation, lipophilicity (comparison of the HPLC chromatograms in Figure S37), and nanoparticle size (NTA analysis in Table S10) show that accumulation in A549 cells closely follows lipophilicity across complexes **1**–**7**. In particular, the most lipophilic complex (**7**) exhibits the highest accumulation. The only deviation from this correlation is observed for complex **5**; however, NTA analysis indicates that this complex forms nanoparticles with a significantly smaller average size, outside the optimal range for efficient cellular accumulation.

Moreover, cellular accumulation studies indicate that the accumulation of Ir­(III) complexes is generally markedly higher in HCC827 cells compared to A549 cells. This increased accumulation correlates with higher cytotoxic activity observed in the HCC827 cell line, suggesting a direct relationship between the level of intracellular complex accumulation and the resultant pharmacological effect. The observed difference in cellular accumulation can be attributed to the mutations in the EGFR gene in HCC827 cells. The activated form of EGFR in these cells is known to enhance various signaling pathways, promoting not only tumor growth but also altering cellular characteristics that could facilitate increased uptake of therapeutic agents.

### Generation of Reactive Oxygen Species after Irradiation with Blue Light

Reactive oxygen species (ROS) are highly reactive byproducts of oxygen metabolism that serve dual roles in biology. At physiological levels, they function as critical signaling mediators, whereas excessive accumulation induces oxidative stress and cellular damage.^70^ This dichotomy is therapeutically exploitable, particularly in oncology.^71^ Chemotherapeutic agents can act by elevating ROS levels,^72^ while photodynamic therapy (PDT) relies on light-activated photosensitizers to produce cytotoxic ROS that selectively eradicate cancer cells.^73^

To evaluate this mechanism, ROS production by complexes **1–7** was assessed in A549 cells following irradiation with blue light (420 nm, 58 W/m^2^). ROS levels were expressed relative to an irradiated control (100%) (Figure 6). Most complexes induced an apparent, dose-dependent increase in ROS beyond the control, confirming their photosensitizing properties. Complex **4** was the most potent, generating more than 350% normalized ROS at 10 μM. Complexes **1**, **2**, **3**, and **5** also produced strong ROS responses, typically exceeding 200% at higher concentrations. In contrast, complex **6** showed weaker activity, peaking slightly above 150%. A strong correlation was observed between ROS generation and antiproliferative efficacy. Complexes with robust ROS production exhibited markedly low irradiated IC_50_ values (e.g., complex **4**: 0.012 μM, complex **5**: 0.14 μM, complex 1:0.23 μM). Conversely, complex **6**, the weakest ROS generator, showed poor activity (IC_50_ = 40.7 μM). These findings highlight ROS generation as a primary driver of the photodynamic efficacy of the investigated iridium­(III) complexes.^74^

**Figure 6:**
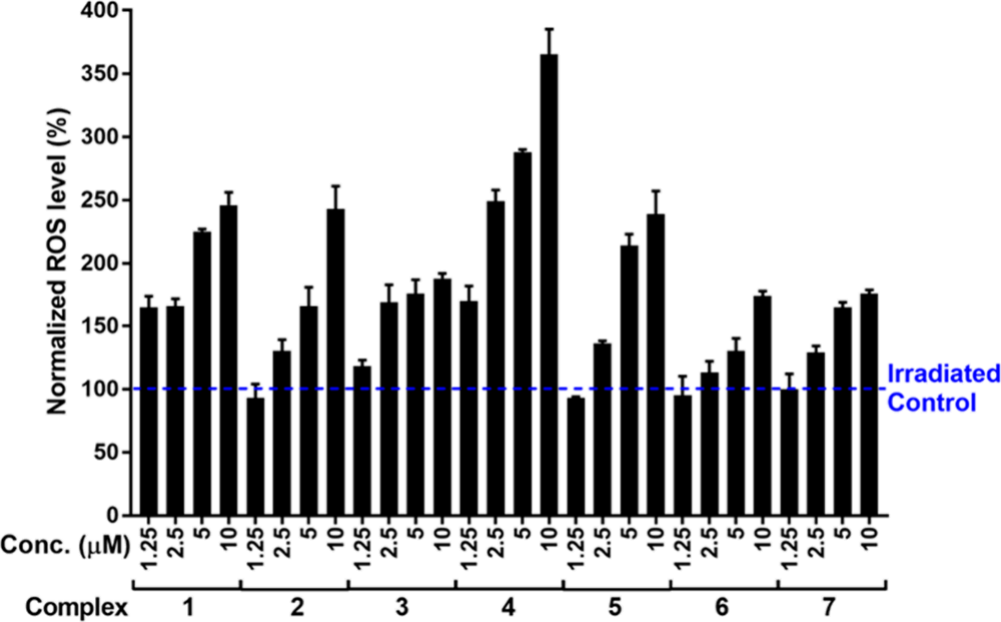
Quantification of reactive oxygen species (ROS) induced by blue light irradiation. A549 cells were pretreated for 1 h with the indicated concentrations of Ir­(III) complexes. Cells were then irradiated for 1 h with blue light. Intracellular ROS were quantified using the CellRox Green probe and analyzed via flow cytometry. ROS levels are normalized to irradiated, untreated control samples. Data represent the mean ± standard deviation from three independent experiments, with duplicate samples in each run.

Complex **4** demonstrated a significant elevation in the levels of ROS in A549 cells upon exposure to blue light irradiation. This finding supports previously established correlations between light-activated iridium complexes and enhanced oxidative stress in cancerous cells, suggesting a potential mechanism for cytotoxicity in tumor therapy. Elevated ROS levels are known to induce oxidative damage within cellular components, leading to apoptosis. The observed increase in ROS following blue light activation of complex **4** aligns with the documented behaviors of similar iridium-based compounds that utilize photodynamic mechanisms to elicit potent anticancer effects. Such mechanisms have been explored in previous studies.

### Intracellular Localization and Colocalization Studies

The efficacy and selectivity of phototoxic compounds are strongly influenced by their intracellular localization, as proximity to specific organelles governs both the nature and efficiency of the phototoxic response.^75^ To investigate this relationship, we first assessed the general subcellular distribution of the investigated complexes in A549 cells. Marked differences in localization patterns (Figure S68) prompted further colocalization studies with organelle-specific markers. Complex **7** was excluded from these experiments because it predominantly associated with the cytoplasmic membrane, forming large aggregates on the cell surface (Figure S68). Given its strong membrane localization and minimal intracellular presence, we did not expect meaningful colocalization with the organelle-specific stains. Therefore, complex **7** was omitted from this analysis. In contrast, complexes **4** and **5** displayed distinct but more nuanced distribution profiles. Considering their pronounced differences in phototoxicity, partly attributable to variations in cellular uptake, particularly for complex **4**, we hypothesized that differential subcellular localization represents an additional determinant of their light-induced cytotoxicity.

In PDT, mitochondrial accumulation is often advantageous, as mitochondrial dysfunction is a potent inducer of apoptosis.^60,76^ Nonetheless, localization to other organelles, including the endoplasmic reticulum (ER) and lysosomes, can also trigger distinct cell death pathways and shape therapeutic outcomes.^74,77^ Guided by these considerations, we examined the colocalization of the most potent complexes **4** and **5** with mitochondria, ER, and lysosomes (Figure 7). Pearson’s colocalization coefficients (PCCs; Table 3) revealed apparent differences: complex **4** exhibited stronger mitochondrial localization (PCC = 0.62) compared to complex **5** (PCC = 0.43), while both complexes associated with the ER to a similar extent (PCC ≈ 0.5). Neither compound showed appreciable lysosomal accumulation (PCC = 0.2 for 4; 0.1 for 5).

**Figure 7:**
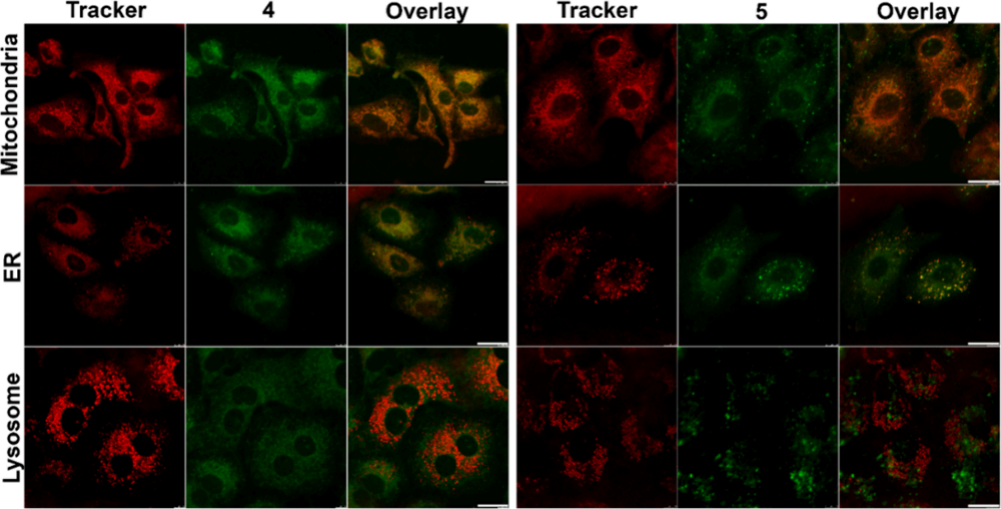
Intracellular colocalization studies of iridium complexes **4** and **5** in A549 cells. A549 cells were incubated with 5 μM iridium complexes for 2 h, followed by costaining with specific cell trackers for mitochondria, endoplasmic reticulum, and lysosomes. Confocal microscopy was used for imaging. Scale bars represent 20 μm.

**Table 3:** Pearson Colocalization Coefficients^a^

Complex	**PCC (Mitochondria)**	**PCC (Endoplasmic Reticulum)**	**PCC (Lysosomes)**
**4**	0.62 ± 0.02	0.5 ± 0.1	0.22 ± 0.08
**5**	0.43 ± 0.06	0.52 ± 0.08	0.10 ± 0.02

a Pearson colocalization coefficients were calculated for candidate iridium complexes and their respective organelle trackers. Values represent the means from three independent experiments ± standard deviations (SDs).

The enhanced ROS generation of complex **4** is likely driven by its preferential mitochondrial localization, where ROS production effectively induces cellular damage and apoptosis.^76^ This mechanistic link explains the superior phototoxicity of **4** and is consistent with the literature,^74,76,78^ which highlights mitochondria as critical targets in PDT. By contrast, the stronger ER association of complex **5** suggests engagement of alternative cell death pathways. Thus, the divergent localization profiles of complexes **4** and **5** - mitochondrial versus ER preference - provide a compelling explanation for their distinct light-induced cytotoxic responses, warranting further mechanistic investigation.

### Analysis of the Mechanism of Cell Death

The cytotoxicity of reactive oxygen species (ROS), particularly singlet oxygen, is strongly influenced by their subcellular localization. Experimental data (see linked in-solution singlet oxygen generation) demonstrate that mitochondrial singlet oxygen, even at low levels, is significantly more damaging than much higher concentrations generated in the nucleus. This heightened vulnerability is attributed to the mitochondria’s high oxygen content and their inherent susceptibility to oxidative stress. Moreover, the mitochondria’s central roles in both energy production and apoptotic regulation provide a compelling rationale for investigating mitochondrial pathways in cancer cell apoptosis.^60,71,79^

Due to their pronounced responsiveness to photoactivation and treatment with the Ir-based complexes under investigation, A549 lung adenocarcinoma cells were selected for apoptosis/necrosis profiling using the standard Annexin V/propidium iodide (PI) assay (Figure S69). Cells were treated with complexes **4, 5**, and **7** at their respective IC_50_ concentrations, incubated in the dark for 1 h, photoirradiated for 1 h, and then cultured in drug-free medium for 18 h. Control experiments confirmed minimal cytotoxicity in the absence of light, underscoring the specificity and safety of PDT.

Upon irradiation with 420 nm blue light, distinct cell populations emerged: Annexin V-positive (early apoptotic) and Annexin V/PI–double-positive (late apoptotic/secondary necrotic), indicating effective PDT-induced apoptosis. Notably, cells did not remain in the early apoptotic state but progressed rapidly to the double-positive phase, suggesting swift plasma membrane permeabilization and late-stage apoptosis.

Complexes **4** and **5** produced comparable proportions of Annexin V-positive cells (∼5%), yet the concentration of complex **5** required was approximately ten times higher than that of complex **4**, highlighting complex **4**’s greater phototoxic efficiency. The double-positive populations were slightly higher for complex **4** (∼18%) than **5** (∼14%). In contrast, complex **7** induced only moderate cell death (∼3.5% double-positive), and most **7**-treated cells remained Annexin V-positive, yielding an Annexin V-to-PI ratio of 0.5. In comparison, complexes **4** and **5** showed a ∼3:1 ratio of double-positive to Annexin V–only cells.

A predominance of double-positive cells indicates rapid apoptotic progression, accompanied by compromised membrane integrity. Conversely, an accumulation of Annexin V–only cells suggests delayed progression or arrest in early apoptosis.^80,81^ Therefore, the higher double-to-single-positive ratios observed with complexes **4** and **5** reflect not only their ability to induce apoptosis efficiently but also to accelerate its execution, consistent with their higher phototoxic potency. The lower ratio observed for complex **7** suggests a slower or less efficient apoptotic process.

Notably, the absence of PI-only cells across all treatments suggests that apoptosis, rather than primary necrosis, is the predominant mode of cell death under these photoactivating conditions.

To further characterize the mode of apoptotic cell death induced by photodynamic treatment, we examined key biochemical markers of the apoptotic cascade. A significant loss of mitochondrial membrane potential (Δψ_m_) was observed (Figure S70), suggesting that mitochondrial dysfunction is a primary determinant of treatment efficacy. This depolarization was accompanied by a robust activation of executioner caspases 3 and 7 (Figure S71). Collectively, these results confirm that the observed cytotoxicity is predominantly mediated through a mitochondria-dependent apoptotic pathway.^82^

### Intracellular NADH Depletion

Mitochondria-targeted photosensitization frequently exploits the essential role of NADH. A significant advancement in anticancer therapy involves the photooxidation of NADH, which disrupts cellular homeostasis—a condition critical for cancer cell survival. By converting NADH to NAD^+^, this approach perturbs intracellular redox equilibrium, ultimately triggering cell death.

To quantify this effect, NADH and NAD^+^ levels were independently measured in A549 cells, and the NADH/NAD^+^ ratio was calculated (Figure 8). Cells were treated with complex concentrations corresponding to their IC_50_ values under irradiation conditions (Table 1). NADH depletion assays were conducted by exposing samples to blue light (420 nm, 1 h, 58 W/m^2^) or maintaining them in darkness before analysis. In the absence of light, no significant differences were observed between treated and control samples. However, upon irradiation, treatment with Ir complexes significantly reduced the NADH/NAD^+^ ratio, indicating effective photoinduced oxidation of NADH in cancer cells.

**Figure 8:**
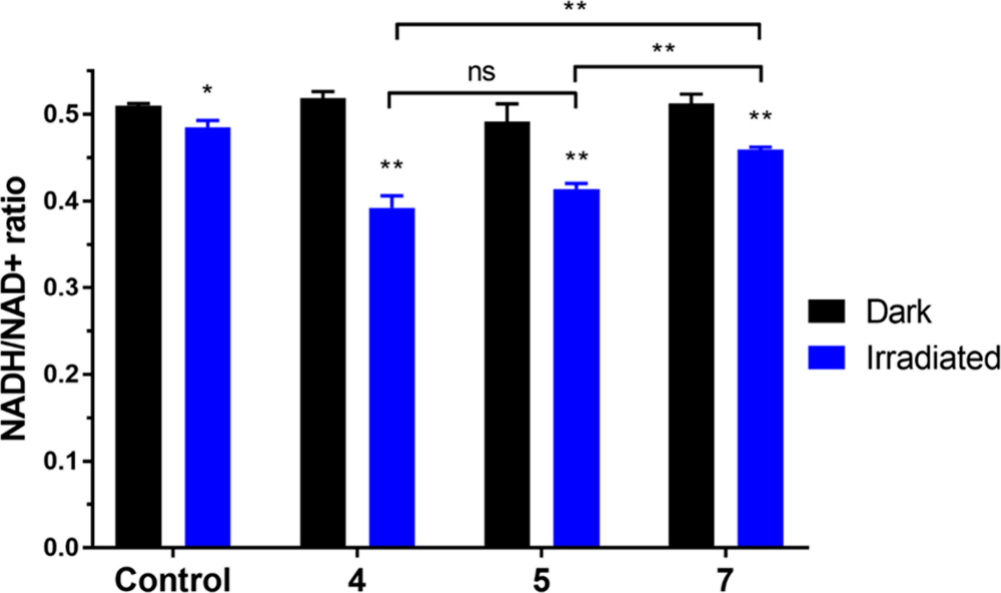
Intracellular NADH depletion in A549 cells. A549 cells were treated with the respective complexes at their IC_50_ concentrations for 2 h (1 h incubation in the dark followed by 1 h irradiation with blue light, 420 nm, 58 W/m^2^). Experiments were performed in duplicate with triplicate samples per condition. Data are shown as mean ± SD. Statistical significance between dark-incubated and irradiated samples was assessed using a two-tailed *t* test. Significance levels are indicated as follows: *P ≤ 0.05, **P ≤ 0.01; “ns” denotes a nonsignificant difference.

The most pronounced effect was observed with complex **4**, which reduced the NADH/NAD^+^ ratio to 0.39, followed by complex **5** (0.41) and **7** (0.45). Control samples also showed a slight reduction (0.51 vs 0.48), suggesting a minor stress response to light exposure alone. These results clearly demonstrate that complexes **4** and **5** are potent photoinducers of NADH oxidation, resulting in a substantial redox imbalance in A549 cells under blue light irradiation.

By disrupting mitochondrial redox homeostasis and energy metabolism, these complexes promote cancer cell death. Among the tested complexes, **4** exhibited the most vigorous activity. Overall, these findings underscore the potential of mitochondria-targeted photocatalysis as a novel therapeutic strategy, offering a selective approach to impair cancer cell viability through metabolic destabilization.

### Phototoxicity toward Multicellular 3D Spheroids

The use of multicellular 3D spheroids is essential for modeling tumors in a physiologically relevant manner, offering greater fidelity to in vivo conditions than conventional 2D cell cultures.^83^ Since A549 cells fail to form spheroids under standard ultralow attachment conditions,^84^ HCC827 cells were selected for 3D culture experiments as a representative model of EGFR mutant lung cancer.^85^

Cell viability and cytotoxicity within HCC827 spheroids were assessed using a combination of Hoechst, Calcein AM, and propidium iodide (PI) staining (Figure 9A). This dye-based approach effectively distinguishes between live and dead cells, allowing for the quantitative evaluation of treatment outcomes. Notably, blue light irradiation significantly enhanced the cytotoxic effect of complex **4**, as indicated by a substantial reduction in the Calcein AM/PI ratio. This decrease reflects a pronounced induction of cell death within the spheroid, supporting the therapeutic potential of photochemical activation in EGFR mutant models.

**Figure 9:**
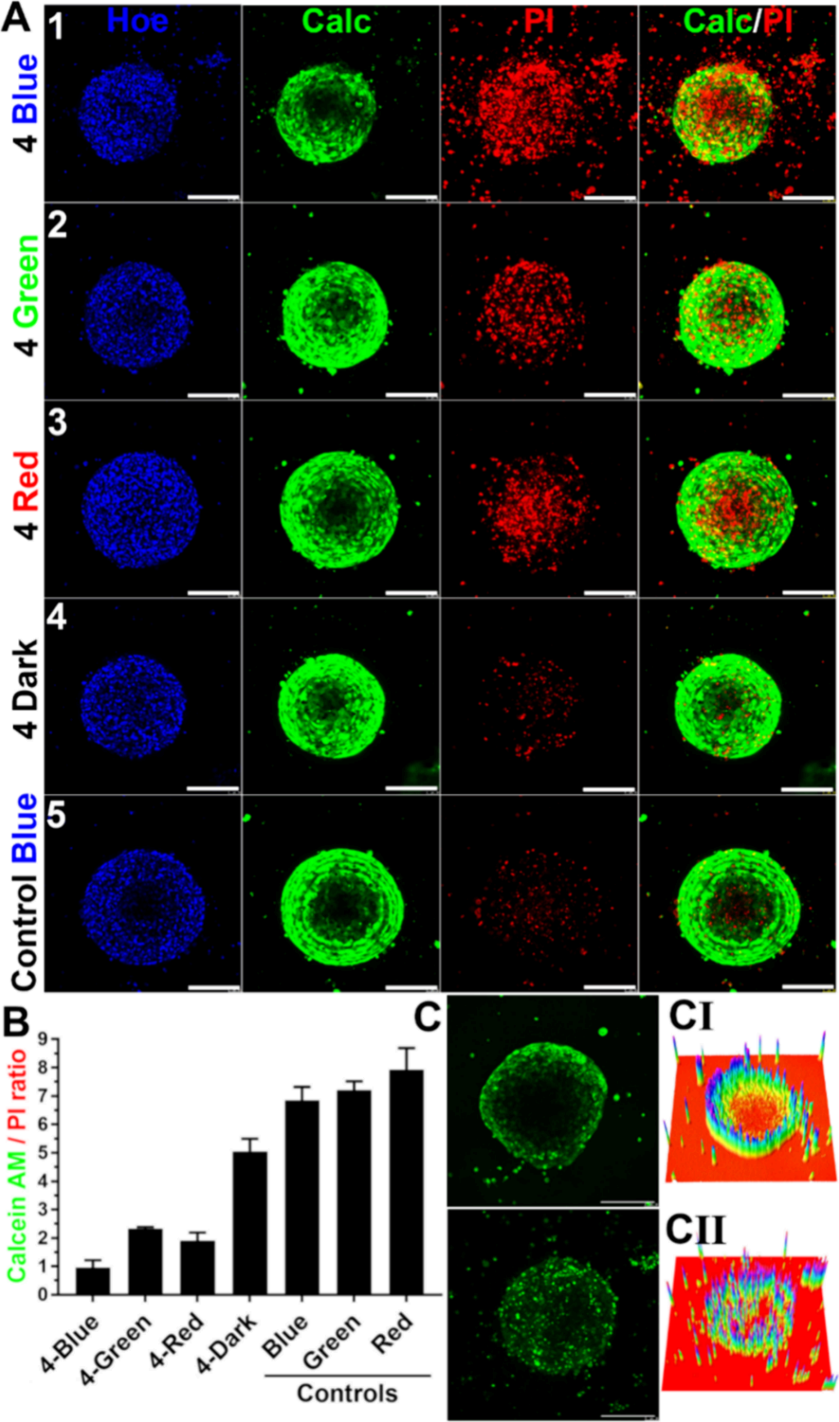
Activity of complex **4** in HCC827 Spheroids. (A) Confocal microphotographs of HCC827 spheroids, stained with Hoechst 33258 (nuclei), Calcein AM (live cells), and Propidium Iodide (dead cells). Complex **4** was added at a concentration of 1 μM for 2 h. Samples were then irradiated for 1 min with 1 mW of: (1) blue (405 nm), (2) green (550 nm), or (3) red (630 nm) laser light; (4) Complex **4** in the dark; or (5) a control (no complex **4**, blue light). Scale bars: 200 μm. (B) Bar chart showing quantification of mean fluorescence ratios (Calcein AM/Propidium Iodide). Panels in section (C) show a short-term (CI) and long-term (CII) distribution of complex **4** in HCC827 spheroids. Panel I shows the accumulation of complex **4** (1 μM) within spheroids 2 h after the incubation with **4** and immediately before blue light irradiation. Panel II illustrates the distribution of complex **4** in spheroids 70 h after the irradiation. Left panels in section C display maximal intensity projections of 10 Z-stacks from a confocal microscope, while right panels present 3D surface plots derived from the maximal fluorescence intensity projection of the Z-stacks. Scale bars: 200 μm.

Complex **4** demonstrated wavelength-dependent photocytotoxicity: after irradiation, Calcein AM/PI ratios dropped to ∼1.0 with blue light (405 nm), and to ∼2.0 and ∼2.5 with red (630 nm) and green (550 nm) light, respectively, compared to their corresponding nontreated controls (Figure 9B). These findings confirm a phototoxic effect of **4** in 3D multicellular spheroids. In the absence of light, **4**-treated spheroids showed a modest but reproducible decrease in viability (Calcein/PI ratio ∼ 5), relative to dark controls (ratio ≥ 7), indicating some baseline cytotoxicity even without photoactivation. In contrast, irradiated control groups lacking complex **4** maintained high viability, confirming that light exposure alone does not compromise spheroid integrity.

Photocytotoxic efficacy in large spheroids is influenced by two primary factors: (i) the compound’s ability to penetrate dense multicellular structures, which simulate the architecture, but not the full complexity, of solid tumors, and (ii) the characteristics of the activating light. Shorter wavelengths (e.g., blue light) generally exhibit lower tissue penetration compared to longer wavelengths.^21^

To evaluate complex **4** distribution within the spheroids, confocal z-stack imaging was performed across the spheroid’s half-volume (Figure 9C). Imaging was conducted both immediately after a 2-h treatment period (Panel I, Figure S72) and again 70 h postirradiation in drug-free conditions (Panel II). Initially, **4** was primarily restricted to the outer 180 μm of the spheroid, highlighting a common limitation in initial drug penetration, which often necessitates repeated treatment-irradiation cycles to achieve complete tumor eradication.^86^

However, by 70 h postirradiation, **4** was observed to have deeply penetrated the spheroid core and persisted within the tissue, suggesting that delayed diffusion and retention can significantly enhance therapeutic reach. This unexpected result indicates that an optimized treatment schedule, combining a single dosing period with a double-irradiation protocol, could achieve complete tumor ablation without requiring repeated administrations.^87^

Overall, these findings reveal that, despite initial limitations in penetration, **4** can achieve durable intratumoral retention and deep tissue infiltration when appropriately scheduled. This opens promising avenues for refining photoactivated treatment regimens aimed at maximizing therapeutic efficacy while reducing the need for repeated interventions, a standard limitation of traditional cancer therapies.

## Discussion and Conclusions

This study demonstrates the strong potential of biscyclometalated Ir­(III) complexes [Ir­(C^N)_2_(bzt-tfb-CO_2_Me-bzim)]^+^ as selective photoactivatable agents for photodynamic therapy against lung and colorectal cancers. To evaluate the clinical safety and selectivity of this series, we incorporated the nonmalignant human lung fibroblast line MRC5. The calculated therapeutic indices under dark conditions (Table 1) establish a baseline safety profile, demonstrating that while the complexes show comparable dark toxicity across cell lines, the true therapeutic window is unlocked upon irradiation. Among the tested complexes, **4** (benzoxazole derivative, pbo) exhibited the most potent activity, achieving nanomolar IC_50_ values in A549 cells under blue light irradiation and maintaining high phototoxic indices in both HCC827 (EGFR mutant) and HCT-116 (colorectal) models. Complex **7** (naphthylbenzimidazole derivative, tfbnapbzim) also showed broad-spectrum photoactivity with consistently high phototoxic indices and minimal dark toxicity—an essential criterion for clinical safety. This safety profile was further validated in MRC-5 cells, where complex **7** remained nontoxic under both light activation and hypoxic stress (IC_50_ ≥ 100 μM). Notably, the efficacy of these complexes in both EGFR wild-type and mutant lung cancer cells suggests a mechanism of action that is independent of specific oncogenic pathways.

Our findings indicate that cellular uptake is a key determinant of this efficacy. The significantly higher accumulation of iridium in HCC827 cells compared to A549 cells (Table 2) directly correlates with the enhanced cytotoxicity in the mutant line, suggesting that EGFR-driven alterations in cellular characteristics may facilitate more efficient internalization of these therapeutic agents. However, uptake is not the sole factor; while complex **7** showed high accumulation, its moderate phototoxicity compared to complex **4** suggests that once internalized, the efficiency of mitochondrial targeting and photochemical activation becomes the decisive factor for cell death.

Mechanistically, photodynamic efficacy correlated strongly with the complexes’ ability to generate reactive oxygen species (ROS) upon light activation. To further define this mechanism, we investigated activity under hypoxic conditions (1% O_2_). Traditional PDT often fails in oxygen-deprived environments due to a reliance on Type II mechanisms. Notably, complex **4** remained highly effective under hypoxia with a submicromolar IC_50_ (0.37 μm) and a PI of 23.5 (Table S13). This resilience strongly suggests that these Ir­(III) complexes operate via a Type I photochemical pathway (radical formation) or direct photoinduced molecular interactions that do not strictly require molecular oxygen, offering a major advantage for treating poorly vascularized solid tumors.

Subcellular localization, particularly to mitochondria, plays a critical role in modulating outcomes. Complex **4** exhibited strong mitochondrial colocalization (PCC = 0.62), which correlates with its superior phototoxic profile. To provide a direct mechanistic link between this localization and cell death, we examined key biochemical markers of the apoptotic cascade. A significant loss of mitochondrial membrane potential (Δψ_m_) was observed (Figure S70), suggesting that mitochondrial dysfunction is a primary event in the treatment’s efficacy. This depolarization was accompanied by a robust activation of executioner caspases 3 and 7 (Figure S71). Collectively, these results confirm that the observed cytotoxicity is predominantly mediated through a mitochondria-dependent apoptotic pathway triggered by mitochondrial targeting, which enhances the impact of even moderate ROS levels.

Annexin V/PI staining confirmed apoptosis as the predominant mode of cell death for complexes **4**, **5**, and **7**.

The use of 3D HCC827 spheroids provided a more physiologically relevant tumor model, revealing critical insights into compound penetration. Complex **4** showed significant drug retention and deep tissue penetration after 70 h, supporting a “single-treatment, double-irradiation” strategy to overcome the penetration limits of solid tumor PDT.

In summary, benzothiazolyl-benzimidazole-based iridium­(III) complexes, particularly complex **4**, offer a promising platform for next-generation PDT agents. Their potency, selective activation, ability to bypass hypoxia-induced resistance, and favorable selectivity profile against nonmalignant MRC5 cells position them as strong candidates for clinical translation. Future studies will focus on in vivo validation and optimization of irradiation protocols to maximize therapeutic efficacy and minimize systemic toxicity.

## Experimental Section

### Materials and Instrumentation

2-Phenylpyridine, benzo­[h]­quinoline, 2-(thiophen-2-yl)­pyridine, 2-phenylbenzo­[d]­oxazole, 2-phenylbenzo­[d]­thiazole, 2-phenyl-1H-benzo­[d]­imidazole, 2-(naphthalen-2-yl)-1H-benzo­[d]­imidazole, 4-(trifluoromethyl)­benzyl bromide, cesium carbonate, silver triflate, potassium and ammonium hexafluorophosphate, methyl 4-chloro-3-nitrobenzoate, (4-(trifluoromethyl)­phenyl)­methanamine, iron powder, ammonium chloride, benzo­[d]­thiazole-2-carbaldehyde and sodium bisulfite were supplied from Merck (Spain). Anhydrous iridium trichloride (IrCl_3_) was purchased from Johnson Matthey. The solvents and liquid reagents (triethylamine, acetonitrile, dichloromethane, chloroform, ethanol, 2-ethoxyethanol and methanol) were supplied from Labkem. HPLC-quality solvents (acetonitrile, dimethyl sulfoxide) were purchased from Scharlab to perform the photophysical characterization of the complexes. Deuterated solvents (CDCl_3_, DMSO-*d*_6_) were acquired from Eurisotop. The ^1^H-, ^13^C-, ^19^F­{^1^H}- and ^31^P NMR spectra were recorded in Bruker AC 300E, Bruker AV 400 and Bruker AV 600 spectrometers. The spectrochemical shifts, given in ppm, were referenced to tetramethylsilane (^1^H and ^13^C NMR), CFCl_3_ (^19^F­{^1^H}-NMR), and phosphoric acid (^31^P NMR). The HR-ESI-MS spectra of the compounds were recorded in an MS/QTOF Agilent 6546A spectrometer. The mobile phase for the injection was acetonitrile (+0.1% formic acid). The data treatment was carried out using the MASS HUNTER software. Elemental analysis (C, H, N, and S) of the ancillary ligand and the iridium­(III) complexes was conducted in a TruSpec Leco microanalyzer equipped with nondispersive IR detectors for C, H, and S, and a thermal conductivity detector for N. A549 and HCC827 lung adenocarcinoma cell lines were from ATCC and colon carcinoma HCT-116 cells were from ECACC.

### Synthetic Protocols

#### Synthesis of Proligands **HC^N6** and **HC^N7**

**HC^N6** and **HC^N7** proligands were prepared as previously reported.^37^

To a suspension of 2-phenyl-1H-benzo­[d]­imidazole or 2-(naphthalen-2-yl)-1H-benzo­[*d*]­imidazole (1.24 mmol) and 4-(trifluoromethyl)­benzyl bromide (339 mg, 1.43 mmol) in acetonitrile (30 mL), Cs_2_CO_3_ (730 mg, 2.24 mmol) was added. The resulting mixture was stirred at room temperature in the dark for 24 h. After the reaction was complete, the mixture was concentrated in vacuo, and the residue was dissolved in CH_2_Cl_2_ and extracted twice with a saturated aqueous NaHCO_3_ solution. The organic layers were dried over anhydrous MgSO_4_ and filtered using a Celite plug. Filtrates were evaporated in the rotary evaporator, and the residue was treated with hexane, obtaining in both cases a white solid, which was filtered and air-dried.

##### 2-Phenyl-1-(4-(trifluoromethyl)­benzyl)-1H-benzo­[*d*]­imidazole (**HC^N6**)

White solid. Yield: 78%. ^1^H NMR (300 MHz, DMSO-*d*_6_) δ = 7.77–7.65 (m, 5H), 7.55–7.45 (m, 4H), 7.30–7.19 (m, 4H), 5.70 (s, 2H). Previously reported in the literature.^45^

##### 2-(Naphthalen-2-yl)-1-(4-(trifluoromethyl)­benzyl)-1H-benzo­[*d*]­imidazole (**HC^N7)**

White solid. Yield: 55%. ^1^H NMR (400 MHz, DMSO-*d*_6_) δ = 8.29 (s, 1H), 8.05 (d, *J* = 8.5 Hz, 1H), 8.00–7.93 (m, 2H), 7.86 (dd, *J* = 1.5, 8.5 Hz, 1H), 7.80–7.78 (m, 1H), 7.66 (d, *J* = 8.1 Hz, 2H), 7.61–7.58 (m, 2H), 7.54–7.52 (m, 1H), 7.30–7.27 (m, 2H), 7.24 (d, *J* = 8.1 Hz, 2H), 5.81 (s, 2H). Previously reported in the literature.^37^

#### Synthesis of Ancillary Ligand **L**

The preparation of the ancillary ligand **L** was carried out in a three-step synthetic route, adapting previous protocols reported in the literature.^38–40^

Under an inert atmosphere, methyl 4-chloro-3-nitrobenzoate (4 g, 18.55 mmol) was dissolved in degassed dichloromethane (40 mL). To the resulting solution 4-(trifluoromethyl)­benzylamine (13 g, 74.21 mmol) and triethylamine (7.5 g, 74.21 mmol) were added. The resulting mixture was refluxed for 72 h. Afterward, the mixture was extracted twice with water (2 × 20 mL). The organic layer was then dried over MgSO_4_ and filtered with a Celite plug. Filtrate was dried in vacuo, and the residue was treated with the minimum volume of ethyl ether, obtaining a yellow solid (intermediate **A**), which was filtered and air-dried. Yield: 5.57 g (84%). Intermediate methyl 3-nitro-4-((4-(trifluoromethyl)­benzyl)­amino)­benzoate (**A**, 1.57 g, 4.61 mmol) was suspended in a water/ethanol mixture (1:4, 30 mL). Next, iron powder (1.28 g, 22.9 mmol) and NH_4_Cl (1.25 g, 22.9 mmol) were added to the suspension, and the resulting mixture was stirred at 90 °C for 24h. After the reaction was completed, the flask content was filtered while hot through a Celite plug, washing twice with ethanol (2 × 5 mL). After that, the filtrate was evaporated, and the residue was dissolved in ethyl acetate (30 mL). The solution was then extracted twice with brine (2 × 12 mL). The organic layer was dried over MgSO_4_ and filtered through a Celite plug. After removing the solvent, the crude was washed with hexane, and a pale salmon-colored solid (intermediate **B**) was filtered and air-dried. Yield: 1.14 g (79%). 1,3-benzothiazole-2-carboxaldehyde (39.4 mg, 0.24 mmol) was added to an aqueous solution of sodium bisulfite (278 mg, 2.65 mmol, 4 mL), and the resulting suspension was refluxed for 30 min. Then, a solution of intermediate methyl 3-amino-4-((4-(trifluoromethyl)­benzyl)­amino)­benzoate (**B**, 75 mg, 0.24 mmol) in ethanol (4 mL) was added dropwise. The resulting solution was stirred at reflux for 24h, obtaining a crude product, which was ultimately purified by column chromatography (silica, eluent: dichloromethane/acetonitrile, 80:20).

##### Methyl 2-(Benzo­[d]­thiazol-2-yl)-1-(4-(trifluoromethyl)­benzyl)-1H-benzo­[*d*]­imidazole-5-carboxylate (**L**)

Yellow solid. Yield: 49%. ^1^H NMR (400 MHz, CDCl_3_) δ = 8.67 (dd, *J* = 1.5, 0.6 Hz, 1H), 8.07–8.03 (m, 2H), 8.00–7.97 (m, 1H), 7.55 (d, *J* = 8.1 Hz, 2H), 7.52–7.46 (m, 2H), 7.39–7.36 (m, 3H), 6.36 (s, 2H), 3.96 (s, 3H) ppm. ^13^C NMR (101 MHz, CDCl_3_) δ = 167.3, 159.0, 153.9, 146.7, 142.7, 140.5, 140.4, 139.7, 135.6, 130.3 (q, *J* = 32.5 Hz), 127.4, 126.8, 126.8, 126.3, 126.1, 126.0 (q, *J* = 3.8 Hz), 124.2, 124.0 (q, *J* = 272.5 Hz), 123.4, 121.9, 110.4, 52.4, 48.6 ppm. ^19^F­{^1^H}-NMR (377 MHz, CDCl_3_) δ = −62.68 (s) ppm. ESI-MS (positive ion mode, CHCl_3_): calc.: [M + H]^+^ = 468.0994 *m*/*z*; exp.: 468.1005 *m*/*z*.

#### Synthesis of the Bis-Cyclometalated Iridium­(III) Complexes (**1–7**)

The synthesis of the iridium­(III) complexes was achieved by adapting a two-step protocol reported in the literature.^15,41–43^

IrCl_3_ (80 mg, 0.27 mmol) and the corresponding HC^N proligand (0.59 mmol) were subjected to three vacuum-nitrogen cycles and dissolved in a degassed 2-ethoxyethanol/water (3:1) mixture (6–8 mL) under an inert atmosphere. Due to their simplicity, the pyridyl-type proligands **HC^N1**-**HC^N3** and iridium trichloride were heated at 180 °C for 10 min in a Monowave Anton Paar Microwave Reactor. Conversely, benzazole-derived dimers **D4–D7** required conventional heating for 24 h at reflux temperature (110 °C). After the reaction was completed, the resultant mixture was left to cool at room temperature. Water was then added, and the yellow to red solids were filtered, washed dropwise with cold ethanol, and air-dried. Yields: 57% (**D1**); 59% (**D2**); 50% (**D3**); 70% (**D4**); 71% (**D5**); 69% (**D6**); 69% (**D7**).

Dimers **D1**–**D7** were used in the next synthetic step without further purification. Pyridyl-type dimers (**D1**–**D3**) and ancillary ligand **L** were mixed under an inert atmosphere at a 1:2 ratio in a degassed dichloromethane/methanol (2:3) mixture (15 mL) and heated at reflux (58 °C) for 24 h. Afterward, the mixture was left to cool at room temperature, and 10 equiv of potassium hexafluorophosphate were added to exchange the chloride counteranion for hexafluorophosphate. The resultant mixture was stirred at room temperature for 1 h, and the solvents were partially removed using a rotary evaporator. Orange to red solids precipitated and were filtered, washed with water, diethyl ether, and air-dried, yielding impure complexes **1–3**. Conversely, the preparation of complexes **4–7** required harsher conditions, as a silver salt was needed to remove the chloride ligands from the dimeric species and force the ancillary ligand to coordinate to the iridium center. Dimers **D4**–**D7**, ancillary ligand **L** and silver triflate were mixed at a 1:2:2 ratio in degassed 2-ethoxyethanol (10 mL). The resultant mixture was heated at 110 °C for 24 h. Thereafter, the solution was filtered while hot through a Celite plug to remove silver chloride, and the filtrate was left to cool to room temperature. Then, 10 equiv of ammonium hexafluorophosphate were added, and the resulting mixture was stirred at room temperature for an additional 24 h to form the hexafluorophosphate salts. Solvent was then removed in a rotary evaporator, yielding orange to dark red impure complexes **4–7**. The purification of complexes **1–7** was performed by column chromatography using alumina, with a dichloromethane/acetonitrile (80:20) mixture as the eluent.

##### [Ir­(2-Phenylpyridine)_2_(methyl 2-(benzo­[*d*]­thiazol-2-yl)-1-(4-(trifluoromethyl)­benzyl)-1H-benzo­[*d*]­imidazole-5-carboxylate)]­(PF_6_) (**1**)

Reddish orange solid. Yield: 28%. ^1^H NMR (600 MHz, DMSO-*d*_6_) δ = 8.37 (d, *J* = 8.3 Hz, 1H), 8.24 (d+d, *J* = 8.6 Hz, 2H), 8.12 (d, *J* = 8.8 Hz, 1H), 8.05 (d, *J* = 8.8 Hz, 1H), 7.96 (d+d, *J* = 5.5 Hz, 2H), 7.92–7.87 (m, 3H), 7.77–7.75 (m, 3H), 7.56 (t, *J* = 7.8 Hz, 1H), 7.46 (d, *J* = 8.0 Hz, 2H), 7.26 (t, *J* = 7.9 Hz, 1H), 7.20 (t, *J* = 7.6 Hz, 1H), 7.17–7.10 (m, 3H), 7.02–6.98 (m, 2H), 6.94 (s, 1H), 6.88 (d, *J* = 8.4 Hz, 1H), 6.43 (qAB, 2H), 6.34 (d, *J* = 7.4 Hz, 1H), 6.29 (d, *J* = 7.4 Hz, 1H), 3.75 (s, 3H) ppm. ^13^C NMR (151 MHz, DMSO-*d*_6_) δ = 166.9, 166.5, 165.2, 157.1, 151.7, 150.4, 149.9, 149.1, 148.5, 144.7, 144.4, 144.0, 139.1, 139.0, 138.8, 138.7, 135.0, 131.5, 131.3, 130.0, 129.9, 128.8, 128.6, 128.4, 127.2, 127.1, 127.0, 126.1, 125.1, 124.9, 124.3, 124.1, 124.0, 123.9, 122.8, 122.5, 121.4, 120.0, 119.8, 119.7, 113.1, 52.0, 48.9 ppm. ^19^F­{^1^H}-NMR (377 MHz, DMSO-*d*_6_) δ = −61.04 (s, 3F), −70.14 (d, *J* = 711.0 Hz, 6F) ppm. ^31^P NMR (121 MHz, DMSO-*d*_6_) δ = −144.15 (sept, *J* = 711.0 Hz) ppm. ESI-MS (positive ion mode, CH_3_CN): calc.: [M-PF_6_]^+^ = 968.1853 *m*/*z*; exp.: 968.1876 *m*/*z*; calc.: [M-PF_6_]^+^·HCl = 1004.1619 *m*/*z*; exp.: 1004.1620 *m*/*z*. Elemental analysis: Calculated for C_46_H_32_F_9_IrN_5_O_2_PS: %C, 49.64; %H, 2.90; %N, 6.29; %S, 2.88. Experimental: %C, 49.46; %H, 2.92; %N, 6.01; %S, 2.70.

##### [Ir­(Benzo­[h]­quinoline)_2_(methyl 2-(benzo­[*d*]­thiazol-2-yl)-1-(4-(trifluoromethyl)­benzyl)-1H-benzo­[*d*]­imidazole-5-carboxylate)]­(PF_6_) (**2**)

Orange solid. Yield: 55%. ^1^H NMR (600 MHz, DMSO-*d*_6_) δ = 8.54 (d, *J* = 8.0 Hz, 2H), 8.33 (d, *J* = 8.3 Hz, 1H), 8.30 (d, *J* = 5.2 Hz, 1H), 8.22 (d, *J* = 5.2 Hz, 1H), 8.07 (d, *J* = 8.8 Hz, 1H), 8.02 (d, *J* = 8.8 Hz, 1H), 7.99 (d, *J* = 8.8 Hz, 1H), 7.95 (dd, *J* = 8.8, 1.5 Hz, 1H), 7.86 (d+d, *J* = 8.9 Hz, 2H), 7.77 (d, *J* = 8.1 Hz, 2H), 7.71 (d, *J* = 7.9 Hz, 1H,), 7.63 (d, *J* = 7.9 Hz, 1H), 7.61–7.55 (m, 2H), 7.50–7.46 (m, 3H), 7.27–7.24 (m, 2H), 7.08 (t, *J* = 7.9 Hz, 1H), 6.78 (d, *J* = 8.6 Hz, 1H), 6.66 (s, 1H), 6.44 (qAB, 2H), 6.38 (d, *J* = 7.3 Hz, 1H), 6.25 (d, *J* = 7.3 Hz, 1H), 3.68 (s, 3H) ppm. ^13^C NMR (151 MHz, DMSO-*d*_6_) δ = 164.9, 157.5, 156.6, 156.2, 152.0, 150.1, 149.7, 149.4, 145.5, 140.9, 140.7, 140.6, 139.2, 139.1, 139.0, 137.7, 137.5, 134.9, 133.8, 133.7, 129.4, 129.0, 128.9, 128.6, 128.5, 128.3, 127.1, 127.0, 126.5, 126.1, 124.3, 124.2, 124.0, 123.0, 122.9, 121.2, 120.6, 120.4, 119.8, 113.1, 51.9, 48.8 ppm. ^19^F­{^1^H}-NMR (377 MHz, DMSO-*d*_6_) δ = −61.04 (s, 3F), −70.14 (d, *J* = 711.0 Hz, 6F) ppm. ^31^P NMR (162 MHz, DMSO-*d*_6_) δ = −146.54 (sept, *J* = 711.0 Hz) ppm. ESI-MS (positive ion mode, CH_3_CN): calc.: [M-PF_6_]^+^ = 1016.1853 *m*/*z*; exp.: 1016.1879 *m*/*z*. Elemental analysis: Calculated for C_50_H_32_F_9_IrN_5_O_2_PS: %C, 51.72; %H, 2.78; %N, 6.03; %S, 2.76. Experimental: %C, 51.40; %H, 2.75; %N, 5.99; %S, 2.59.

##### [Ir­(2-(Thiophen-2-yl)­pyridine)_2_(methyl 2-(benzo­[*d*]­thiazol-2-yl)-1-(4-(trifluoromethyl)­benzyl)-1H-benzo­[d]­imidazole-5-carboxylate)]­(PF_6_) (**3**)

Reddish orange solid. Yield: 20%. ^1^H NMR (600 MHz, DMSO-*d*_6_) δ = 8.39 (d, *J* = 8.3 Hz, 1H), 8.13 (d, *J* = 8.8 Hz, 1H), 8.08 (dd, *J* = 8.8, 0.8 Hz, 1H), 7.83 (d, *J* = 4.7 Hz, 1H), 7.79–7.71 (m, 8H), 7.66 (d, *J* = 5.8 Hz, 1H), 7.60 (dd, *J* = 8.3, 7.7 Hz, 1H), 7.47 (d, *J* = 8.1 Hz, 2H), 7.38 (dd, *J* = 8.6, 7.7 Hz, 1H), 6.98–6.92 (m, 3H), 6.86 (d, *J* = 8.6 Hz, 1H), 6.42 (qAB, 2H), 6.31 (d, *J* = 4.7 Hz, 1H), 6.27 (d, *J* = 4.7 Hz, 1H), 3.81 (s, 3H) ppm. ^13^C NMR (151 MHz, DMSO-*d*_6_) δ = 165.3, 163.1, 162.8, 157.2, 151.8, 151.1, 150.5, 149.7, 149.0, 144.8, 139.4, 139.3, 139.1, 139.0, 138.9, 136.7, 136.3, 134.9, 131.0, 130.7, 130.6, 130.5, 128.9, 128.8, 128.5, 127.4, 127.3, 127.1, 126.1, 124.4, 124.0, 121.2, 121.0, 119.9, 118.1, 118.0, 113.2, 52.2, 48.8 ppm. ^19^F­{^1^H}-NMR (377 MHz, DMSO-*d*_6_) δ = −61.04 (s, 3F), −70.14 (d, *J* = 711.0 Hz, 6F) ppm. ^31^P NMR (121 MHz, DMSO-*d*_6_) δ = −144.15 (sept, *J* = 711.0 Hz) ppm. ESI-MS (positive ion mode, CH_3_CN): calc.: [M-PF_6_]^+^ = 980.0981 *m*/*z*; exp.: 980.1014 *m*/*z*. Elemental analysis: Calculated for C_42_H_28_F_9_IrN_5_O_2_PS_3_: %C, 44.84; %H, 2.51; %N, 6.22; %S, 8.55. Experimental: %C, 44.74; %H, 2.65; %N, 6.08; %S, 8.72.

##### [Ir­(2-Phenylbenzo­[d]­oxazole)_2_(methyl 2-(benzo­[d]­thiazol-2-yl)-1-(4-(trifluoromethyl)­benzyl)-1H-benzo­[*d*]­imidazole-5-carboxylate)]­(PF_6_) (**4**)

Light orange solid. Yield: 19%. ^1^H NMR (600 MHz, DMSO-*d*_6_) δ = 8.43 (d, *J* = 8.3 Hz, 1H), 8.25 (d, *J* = 8.6 Hz, 1H), 8.12 (dd, *J* = 8.6, 0.7 Hz, 1H), 7.96–7.89 (m, 4H), 7.63 (td, *J* = 7.9, 0.6 Hz, 1H), 7.52 (d, *J* = 8.2 Hz, 2H), 7.45–7.39 (m, 2H), 7.37–7.31 (m, 4H), 7.26 (td, *J* = 7.6, 0.6 Hz, 1H), 7.20–7.17 (m, 2H), 7.13 (td, *J* = 7.6, 1.3 Hz, 1H), 7.00 (d, *J* = 0.7 Hz, 1H), 6.97 (td, *J* = 7.6, 0.8 Hz, 1H), 6.92 (d, *J* = 8.6 Hz, 1H), 6.69–6.67 (m, 2H), 6.42 (qAB, 2H), 5.88 (d, *J* = 8.1 Hz, 1H), 5.51 (d, *J* = 8.1 Hz, 1H), 3.74 (s, 3H) ppm. ^13^C NMR (151 MHz, DMSO-*d*_6_) δ = 177.0, 176.5, 165.1, 157.8, 152.0, 149.9, 149.7, 149.6, 147.6, 143.1, 139.9, 139.1, 139.0, 136.8, 136.5, 134.4, 133.2, 133.1, 133.0, 132.8, 129.2, 129.1, 129.0, 128.8, 128.7, 127.5, 127.1, 126.8, 126.7, 126.6, 126.5, 126.3, 126.2, 126.0, 124.7, 124.9, 123.7, 123.4, 121.6, 120.0, 114.3. 113.8, 113.5, 113.0, 112.9, 52.1, 48.8 ppm. ^19^F­{^1^H}-NMR (377 MHz, DMSO-*d*_6_) δ = −61.03 (s, 3F), −70.13 (d, *J* = 711.0 Hz, 6F) ppm. ^31^P NMR (162 MHz, DMSO-*d*_6_) δ = −146.54 (sept, *J* = 711.0 Hz) ppm. ESI-MS (positive ion mode, CH_3_CN): calc.: [M-PF_6_]^+^ = 1048.1751 *m*/*z*; exp.: 1048.1790 *m*/*z*. Elemental analysis: Calculated for C_50_H_32_F_9_IrN_5_O_4_PS: %C, 50.34; %H, 2.70; %N, 5.87; %S, 2.69. Experimental: %C, 50.56; %H, 2.66; %N, 5.86; %S, 2.67.

##### [Ir­(2-Phenylbenzo­[d]­thiazole)_2_(methyl 2-(benzo­[d]­thiazol-2-yl)-1-(4-(trifluoromethyl)­benzyl)-1H-benzo­[*d*]­imidazole-5-carboxylate)]­(PF_6_) (**5**)

Red solid. Yield: 35%. ^1^H NMR (600 MHz, DMSO-*d*_6_) δ = 8.40 (d, *J* = 8.4 Hz, 1H), 8.22–8.20 (m, 2H), 8.16 (d, *J* = 8.2 Hz, 1H), 8.12 (d, *J* = 8.6 Hz, 1H), 8.06–8.04 (m, 2H), 7.63 (dd, *J* = 8.4, 7.6 Hz, 1H), 7.56 (d, *J* = 8.2 Hz, 2H), 7.39 (dd, *J* = 8.2, 7.7 Hz, 1H), 7.37–7.31 (m, 3H), 7.23–7.17 (m, 3H), 7.16 (dd, *J* = 8.6, 7.7 Hz, 1H), 7.06 (t, *J* = 7.5 Hz, 1H), 7.00 (t, *J* = 7.5 Hz, 1H), 6.93–6.90 (m, 2H), 6.81 (d, *J* = 8.6 Hz, 1H), 6.45–6.33 (m, 5H), 6.18 (d, *J* = 8.6 Hz, 1H), 3.77 (s, 3H) ppm. ^13^C NMR (151 MHz, DMSO-*d*_6_) δ = 181.4, 180.8, 165.0, 157.1, 151.4, 149.2, 148.6, 148.1, 148.0, 143.4, 140.8, 140.6, 138.9–138.8 (m), 134.4, 132.8, 132.6, 132.0, 131.9, 131.1, 131.0, 129.1, 128.7, 128.7, 128.2, 127.8, 127.6, 126.9, 126.6, 125.9, 124.8, 124.6, 124.5, 123.8, 123.7, 123.3, 121.0, 119.7, 117.0, 116.9, 113.4, 52.0, 48.7 ppm. ^19^F­{^1^H}-NMR (377 MHz, DMSO-*d*_6_) δ = = −61.03 (s, 3F), −70.13 (d, *J* = 711.0 Hz, 6F) ppm. ^31^P NMR (121 MHz, DMSO-*d*_6_) δ = −144.15 (sept, *J* = 711.0 Hz) ppm. ESI-MS (positive ion mode, CH_3_CN): calc.: [M-PF_6_]^+^ = 1080.1294 *m*/*z*; exp.: 1080.1322 *m*/*z*. Elemental analysis: Calculated for C_50_H_32_F_9_IrN_5_O_2_PS_3_: %C, 49.02; %H, 2.63; %N, 5.72; %S, 7.85. Experimental: %C, 49.28; %H, 2.93; %N, 5.45; %S, 7.73.

##### [Ir­(2-Phenyl-1-(4-(trifluoromethyl)­benzyl)-1H-benzo­[*d*]­imidazole)_2_(methyl 2-(benzo­[d]­thiazol-2-yl)-1-(4-(trifluoromethyl)­benzyl)-1H-benzo­[*d*]­imidazole-5-carboxylate)]­(PF_6_) (**6**)

Dark red solid. Yield: 21%. ^1^H NMR (600 MHz, DMSO-*d*_6_) δ = 8.42 (d, *J* = 8.3 Hz, 1H), 8.23 (d, *J* = 8.8 Hz, 1H), 8.12 (dd, *J* = 8.8, 1.0 Hz, 1H), 7.88 (d, *J* = 8.,1 Hz, 1H), 7.84–7.83 (m, 2H), 7.79 (d, *J* = 8.3 Hz, 1H), 7.63 (dd, *J* = 8.3, 7.7 Hz, 1H), 7.54–7.52 (m, 4H), 7.34 (d, *J* = 8.1 Hz, 2H), 7.28–7.25 (m, 2H), 7.23–7.19 (m, 3H), 7.13–7.11 (m, 3H), 7.03 (dd, *J* = 8.1, 7.6 Hz, 1H), 7.01–6.98 (m, 3H), 6.96 (t, *J* = 7.6 Hz, 1H), 6.91 (t, *J* = 7.6 Hz, 1H), 6.86 (d, *J* = 8.6 Hz, 1H), 6.82–6.79 (m, 2H), 6.49 (d, *J* = 7.6 Hz, 1H), 6.47–6.24 (m, 7H), 5.96 (d, *J* = 8.3 Hz, 1H), 5.74 (d, *J* = 8.3 Hz, 1H), 3.57 (s, 3H) ppm. ^13^C NMR (151 MHz, DMSO-*d*_6_) δ = 164.9, 162.9, 162.4, 156.9, 151.8, 150.3, 149.7, 145.8, 140.8, 139.3, 138.9, 138.3, 138.0, 135.2, 135.1, 134.4, 133.9, 133.8, 133.5, 133.1, 130.4, 130.3, 128.9–128.1 (m), 127.4, 127.3, 126.7, 126.6, 126.3, 126.0, 125.8, 125.4, 124.8, 124.7, 124.5, 124.0, 123.9, 123.9, 123.8, 123.8, 122.9, 122.5, 121.7, 120.2, 113.6, 113.5, 113.4, 112.0, 51.7, 48.8, 46.8, 46.7 ppm. ^19^F­{^1^H}-NMR (377 MHz, DMSO-*d*_6_) δ = −61.06 (s, 3F), −61.19 (s, 3F), −61.21 (s, 3F), −70.14 (d = 711.0 Hz, 6F) ppm. ^31^P NMR (121 MHz, DMSO-*d*_6_) δ = −144.15 (sept, *J* = 711.0 Hz) ppm. ESI-MS (positive ion mode, CH_3_CN): calc.: [M-PF_6_]^+^ = 1362.2757 *m*/*z*; exp.: 1362.2791 *m*/*z*. Elemental analysis: Calculated for C_66_H_44_F_15_IrN_7_O_2_PS: %C, 52.59; %H, 2.94; %N, 6.50; %S, 2.13. Experimental: %C, 52.65; %H, 2.93; %N, 6.29; %S, 2.19.

##### [Ir­(2-(Naphthalen-2-yl)-1-(4-(trifluoromethyl)­benzyl)-1H-benzo­[*d*]­imidazole)_2_(methyl 2-(benzo­[d]­thiazol-2-yl)-1-(4-(trifluoromethyl)­benzyl)-1H-benzo­[*d*]­imidazole-5-carboxylate)]­(PF_6_) (**7**)

Dark red solid. Yield: 32%. ^1^H NMR (600 MHz, DMSO-*d*_6_) δ = 8.58 (s, 1H), 8.55 (s, 1H), 8.43 (d, *J* = 8.3 Hz, 1H), 8.22 (d, *J* = 8.8 Hz, 1H), 8.03 (d, *J* = 8.8 Hz, 1H), 8.01–7.97 (m, 2H), 7.89–7.88 (m, 1H), 7.84–7.82 (m, 1H), 7.60 (d, *J* = 8.2 Hz, 2H), 7.57–7.54 (m, 3H), 7.44 (d, *J* = 8.2 Hz, 2H), 7.40 (d, *J* = 8.2 Hz, 2H), 7.38–7.27 (m, 7H), 7.25–7.23 (m, 4H), 7.21–7.19 (m, 1H), 7.07–7.03 (m, 3H), 6.87–6.84 (m, 2H), 6.73 (s, 1H), 6.69 (s, 1H), 6.49–6.46 (m, 6H), 6.04 (d, *J* = 8.3 Hz, 1H), 5.74 (d, *J* = 8.3 Hz, 1H), 2.92 (s, 3H) ppm. ^13^C NMR (151 MHz, DMSO-*d*_6_) δ = 164.6, 161.6, 161.2, 157.1, 151.9, 149.9, 142.5, 141.2, 141.0, 139.5, 139.3, 138.9, 138.7, 138.1, 138.0, 135.8, 135.7, 134.4, 133.9, 133.8, 133.5, 133.3, 130.0, 129.7, 129.4, 129.3, 128.9–128.1 (m), 127.8, 127.6, 127.4, 127.1, 127.0, 126.8, 126.7, 126.2, 126.1, 126.0, 125.9, 125.8, 125.5, 125.5, 124.9, 124.8, 124.7, 124.5, 124.4, 124.0, 123.9, 123.9, 123.8, 121.6, 120.1, 114.1, 113.7, 113.4, 112.4, 51.4, 48.8, 46.9, 46.8 ppm. ^19^F­{^1^H}-NMR (377 MHz, DMSO-*d*_6_) δ = = −61.16 (s, 3F), −61.19 (s, 3F), −61.27 (s, 3F), −70.14 (d = 711.0 Hz, 6F) ppm. ^31^P NMR (121 MHz, DMSO-*d*_6_) δ = −144.15 (sept, *J* = 711.0 Hz) ppm. ESI-MS (positive ion mode, CH3CN): calc.: [M-PF_6_]^+^ = 1462.3070 *m*/*z*; exp.: 1462.3102 *m*/*z*. Elemental analysis: Calculated for C_74_H_48_F_15_IrN_7_O_2_PS: %C, 55.29; %H, 3.01; %N, 6.10%S, 1.99. Experimental: %C, 55.39; %H, 2.93; %N, 5.90; %S, 1.87.

#### Purity of the Complexes

The purity of complexes **1–7** was assessed by high-performance liquid chromatography (HPLC). The HPLC chromatograms were registered in an Agilent 1290 Infinity chromatograph, equipped with a C18 column (1.8 μm) and a DAD (set to 400 nm). Complexes were dissolved in acetonitrile, and the mobile phase comprised a mixture of A (water +0.1% formic acid) and B (acetonitrile +0.1% formic acid), with a gradient of 90%A/10%B (0–14 min), 10%A/90%B (14–20 min) and 90%A/10%B (20–30 min). The flow was set at 0.6 mL/min. All compounds were found to be ≥95% pure by HPLC analysis.

#### X-ray Diffraction Studies

Single crystals were obtained by slow evaporation of dichloromethane/hexane solutions of the complexes at room temperature for 3 days. Crystals were mounted on glass fibers and transferred to the cold gas stream of the Bruker Smart APEX diffractometer. Data were recorded with Mo Kα radiation (λ = 0.71073 Å) in ω scan mode. CRYSALISPRO was used for cell refinement, data reduction, and absorption correction.^88^ The crystal structures were solved using OLEX2–1.52 ^89^ with SHELXT and refined with SHELXL.^90,91^ All non-hydrogen atoms were refined with anisotropic displacement parameters. All hydrogen atoms on C were positioned geometrically. The intermolecular interactions were calculated with PLATON for Windows^92,93^ and the graphics were prepared with DIAMOND.^94^ The CCDC reference number is 2498248 and 2498249 for complexes **2** and **5**, respectively, and the data can be obtained free of charge from www.ccdc.cam.ac.uk/data_request/cif.

### Chemical Stability

The chemical stability of the cyclometalated iridium­(III) complexes was first assessed in DMSO by UV/vis absorption spectroscopy. Solutions of complexes **1–7** (10 μM) in DMSO were prepared, and their UV/vis absorption spectra were recorded before (t = 0) and after (t = 48 h) incubation at room temperature in the dark. The stability under more complex physiological and biological conditions was assessed by UV/vis absorption spectroscopy and HPLC/HR-ESI-MS spectrometry. For this purpose, solutions of complexes **1–7** (10 μM) in a DMEM (+10% FBS)/DMSO (95:5) mixture were prepared, and the resulting UV/vis absorption and HPLC-MS spectra were recorded right after solution and after 48 h of incubation at 37 °C.

### Aggregation in Solution

The aforementioned DMEM (+10% FBS)/DMSO (95:5) solutions of complexes **1–7** were subjected to nanoparticle tracking analysis (NTA) using a NanoSight NS300 (Malvern, UK) instrument, equipped with a 488 nm blue laser module and syringe pump. For each measurement, 2 min video was recorded with the cell temperature set to 25 °C and syringe speed set to 40 μL/s. Captured videos were analyzed by the supplied NanoSight Software NTA 3.1 with a detection threshold of 5. The aggregation process was further investigated by observation of the Tyndall effect after pointing the cuvettes with a regular laser.

### Photophysical Characterization

Stock solutions of complexes **1–7** were prepared in acetonitrile and DMSO at a concentration of 2 mM. Thereafter, solutions in acetonitrile and water (1% DMSO) were prepared at a final concentration of 10 μM. The UV/vis absorption spectra were recorded in a PerkinElmer Lambda 750 S spectrometer, and the emission spectra (λ_exc_ = 420 nm) were registered in a Horiba Jobin Yvon Fluorolog 3–22 spectrofluorometer, equipped with a 450 W xenon lamp. The spectra were depicted using GraphPad Prism 5.01 software. The photoluminescence quantum yields (Φ_PL_) were determined in deaerated and aerated H_2_O/DMSO (99:1) solutions with a Hamamatsu C11347 absolute PL quantum yield spectrometer. The estimated uncertainty was found to be 10% or better in all measurements. The emissive behavior of complexes **1–7** in aqueous solutions was studied by emission spectroscopy. For this purpose, aqueous solutions of all the iridium complexes (10 μM) were prepared in different volumetric water fractions (from 0 to 99% water v/v), and the resulting emission spectra were recorded (λ_exc_ = 420 nm). Pictures of the emission intensities and colors were taken upon light irradiation with a short-wavelength lamp.

### Photostability

The photostability of the new iridium­(III) complexes was assessed by UV/vis absorption spectroscopy. Solutions of complexes **1–7** (10 μM) in DMSO were prepared, and their UV/vis absorption spectra were recorded before (*t* = 0) and after (*t* = 1 h) blue (λ_irradiation_ = 465 nm, 5 mW/cm^2^) light irradiation.

### Singlet Oxygen Production

The quantitative evaluation of the photocatalytic ability of complexes **1–7** to produce singlet oxygen was carried out using an indirect method.^95^ For this purpose, 1,3-diphenylisobenzofuran (DPBF, 50 μM in acetonitrile), a well-recognized singlet oxygen scavenger, was incubated with complexes **1–7** (30–50 μM) or [Ru­(2,2′-bipyridine)_3_]­(Cl)_2_ (5 μM) as a reference (Φ_Δ_ = 0.56).^95^ The concentrations of the reference and complexes **1–7** were adjusted to achieve similar optical densities at the irradiation wavelength (λ = 465 nm). Thereafter, solutions were irradiated at very short periods of time (15 s) with a blue lamp (Luzchem LED, λ = 465 nm, 0.45 mW/cm^2^), and the absorption profile of DPBF (λ_abs_ = 411 nm) was monitored and plotted against the irradiation time. Singlet oxygen quantum yield (Φ_Δ_) was calculated as follows:

$$\Phi_{\Delta ,S}=\Phi_{\Delta ,\mathrm{ref}}\times \frac{{\mathrm{Slope}}_{s}}{{\mathrm{Slope}}_{\mathrm{ref}}}\times \frac{1-10^{{\mathrm{Abs}}_{\mathrm{ref}}}}{1-10^{{\mathrm{Abs}}_{s}}}$$ 1

where Φ_Δ,ref_ corresponds to the singlet oxygen quantum yield value obtained for reference [Ru­(2,2′-bipyridine)_3_]­(Cl)_2_ (0.56), slope_s_ and slope_ref_ denote the slope values calculated for the complex and the reference retrieved from the absorbance/irradiation time plots, respectively, and Abs_s_ and Abs_ref_ represent the absorbance intensities of the sample and reference, respectively.

### Detection of Type I ROS Species (HO^·^, O_2_^·–^, H_2_O_2_) in Cell-Free Media

The generation of type I ROS species in solution upon light irradiation was studied by emission spectroscopy. Briefly, HPF (HO^·^ probe, 10 μM), DHR123 (O_2_^·–^ probe, 10 μM) or Amplex Red (H_2_O_2_ probe, 10 μM) were incubated with complexes **1–7** (10 μM for the detection of HO^·^ and O_2_^·–^, 5 μM for the detection of H_2_O_2_) in a H_2_O/DMF (95:5) mixture. Thereafter, blue light irradiation (Luzchem LED, λ = 465 nm, 5 mW/cm^2^) was applied to the cuvette at short intervals, and the emission of the resulting fluorescent products was subsequently monitored (for HPF and DHR123: λ_exc_ = 490 nm, recording range: 500–600 nm; for Amplex Red: λ_exc_ = 550 nm, recording range: 560–700 nm).

### Photo-Oxidation of Biomolecules

Solutions of NADH, 9-ethylguanine, glutathione, ascorbic acid and essential amino acids (Cys, His, Pro) (1 mM) were incubated with complexes **1–7** (10 μM) in a PBS/DMF (95:5) mixture and exposed to blue light irradiation (Luzchem LED, λ = 465 nm, 5 mW/cm^2^) for 1 h. Afterward, hydrogen peroxide stripes (Dimercos, Spain), were dipped into the solutions and the formation of H_2_O_2_ was observed by the coloring of the stripe.

### NADH Photo-Oxidation

NADH (100 μM) was incubated in a PBS/DMF (95:5) mixture with complexes **1–6** (5 μM) or complex **7** (2.5 μM) and its absorption profile was monitored upon irradiation with a blue lamp (Luzchem LED, λ = 465 nm, 5 mW/cm^2^) at short intervals. The photocatalytic parameter of turnover number (TON) was calculated as follows:

$$\mathrm{TON}=\frac{\mathrm{converted}\;\mathrm{NADH}\;\mathrm{moles}}{\mathrm{catalyst}\;\mathrm{moles}}=\frac{[\mathrm{NADH}{]}_{\mathrm{convert}.}}{\left[\mathrm{cat}.\right]}=\frac{[\mathrm{NADH}{]}_{0}-[\mathrm{NADH}{]}_{f}}{\left[\mathrm{cat}.\right]}=\frac{\frac{A(339{)}_{0}-A(339{)}_{f}}{b\times \varepsilon }}{\left[\mathrm{cat}.\right]}$$ 2

where A(339)_0_ and A(339)_f_ correspond to the NADH absorbance intensity at 339 nm before and after irradiation, respectively, b denotes the optical cuvette pathway, ε represents the absorptivity molar coefficient at 339 nm (6220 M^–1^ cm^–1^), and [cat.] corresponds to the concentration of complexes **1–7** (in M).

Turnover frequency (TOF, h^–1^) was calculated dividing the TON number by the irradiation time:

$$\mathrm{TOF}\;(h^{-1})=\frac{\mathrm{TON}}{\mathrm{irradiation}\;\mathrm{time}\;(h)}$$ 3

For comparative purposes, the NADH absorption profile was also monitored upon incubation with complexes **1–7** in dark conditions.

### Scavenger Assays for NADH Oxidation

To verify the impact of HO^·^, O_2_^·–^, H_2_O_2_ and oxygen molecules in the photooxidative cycle of NADH, the NADH photooxidation assay was repeated as indicated above but in the additional presence of mannitol (HO^·^ scavenger), disodium 4,5-dihydroxy-1,3-benzenedisulfonate (tiron, O_2_^·–^ scavenger), sodium pyruvate (H_2_O_2_ scavenger), 6-hydroxy-2,5,7,8-tetramethylchroman-2-carboxylic acid (trolox, a general type I ROS scavenger) or under inert conditions (deareated hypoxic conditions). For this purpose, NADH (100 μM) was incubated in a PBS/DMF (95:5) mixture in the presence of complexes **1–7** (5 μM) and mannitol (20 mM)/tiron (10 mM)/sodium pyruvate (10 mM)/trolox (0.1 mM) under normoxic or hypoxic (previous deaeration of the cuvette by bubbling argon for 30 min) conditions. The resulting solutions were subjected to blue light irradiation (Luzchem LED, λ = 465 nm, 5 mW/cm^2^) for 10–15 min, and the TOF values were calculated following eqs 2 and 3.

### Cell Culture and Antiproliferative Activity Assay

The antiproliferative activity of the compounds was assessed using the MTT (3-(4,5-dimethylthiazol-2-yl)-2,5-diphenyltetrazolium bromide) assay. All cell lines from the screening panel were seeded in 96-well plates at a density of 3000 cells/well in 100 μL of culture medium. Following overnight incubation in a humidified CO_2_ incubator, the cell culture medium was aspirated and replaced with 200 μL/well of Earle’s Balanced Salt Solution (EBSS) containing the investigated compounds. Cells were then exposed to the compounds for 1 h in the dark (5% CO_2_, 37 °C). To simulate tumor hypoxia, experiments were performed at 1% O_2_ (balanced with 5% CO_2_ and 94% N_2_). Cells were maintained in an Invivo2 400 physiological oxygen workstation (Baker Ruskinn) for the duration of the study, ensuring that the hypoxic environment was not disrupted during treatment or irradiation. Subsequently, cells were irradiated with blue light (420 nm) for 60 min at an intensity of 58 W/m^2^. After treatment, the samples were washed with PBS and replenished with 200 μL of fresh culture medium. After a 70-h incubation period, 20 μL of MTT solution (1.25 mg/mL) was added to each well. After a further 3-h incubation, the medium was aspirated, and the wells were air-dried. The formazan product was then dissolved by adding 100 μL of dimethyl sulfoxide (DMSO) to each well. Cell viability was determined by measuring the absorbance at 570 nm with a reference wavelength of 620 nm using an Absorbance Reader (SPARK TECAN, SCHOELLER). Absorbance values were converted to the percentage of cell survival relative to untreated control cells. The antiproliferative effects were expressed as IC_50_ values, which were calculated from dose–response curves generated by plotting the percentage of cell survival against the drug concentration (μM). The IC_50_ value represents the concentration of the compound that inhibits cell growth by 50%.

### Compound Concentration Verification

The concentrations of the compounds in the cell culture medium during treatment were verified using either Flame Atomic Absorption Spectrometry (FAAS) or Inductively Coupled Plasma – Mass Spectrometry (ICP-MS).

### Cellular Uptake

We investigated the cellular uptake of the Ir­(III) complexes in A549 and HCC827 cell lines. Cells were seeded at a density of 1.5 × 10^6^ cells per 100 mm Petri dish and allowed to preincubate overnight. Subsequently, cells were treated with 2 μM of the Ir­(III) compounds for 24 h. Following treatment, cells were harvested, counted, and washed twice with ice-cold Phosphate-Buffered Saline (PBS). Cell pellets were collected by centrifugation. To ensure the integrity of the harvested cells and rule out any potential issues with cell membrane permeability, cell viability was assessed using a trypan blue exclusion test. Finally, the isolated cell pellets underwent microwave-assisted acid digestion using 5 M HCl in a CEM Mars system. The intracellular accumulation of iridium was then quantified by Inductively Coupled Plasma – Mass Spectrometry (ICP-MS).

### Quantification of ROS by Flow Cytometry

Intracellular reactive oxygen species (ROS) generation was evaluated in A549 cells. Cells were seeded at a density of 2 × 10^4^ cells/well in U-shaped 96-well plates and incubated overnight to allow for adherence. The cells were then treated with the test complexes at concentrations ranging from 1.25 to 10 μM for 1 h in the dark. This was followed by 1 h of irradiation with blue light (420 nm, 58 W/m^2^). Following treatment, cells were washed with PBS and subsequently stained with CellRox Green according to the manufacturer’s instructions. Samples were analyzed using a CellStream flow cytometer, and the resulting data were processed with CellStream analysis software.

### Cellular Localization of Ir­(III) Compounds and Co-Localization Studies with Cellular Organelles

The subcellular localization of candidate Ir complexes (**4**, **5**, and **7**) was assessed in A549 cells. Cells were seeded onto glass-bottom confocal dishes (35 mm, MatTek) at a density of 1.2 × 10^5^ cells/dish and cultured overnight in phenol red-free DMEM. Cells were then treated with 5 μM of each complex for 2 h. Following incubation, the medium was replaced with fresh DMEM after washing, and samples were imaged using a Leica SP5 confocal microscope (excitation: 405 nm; emission: 570–650 nm). Images were processed and analyzed with ImageJ software.

For colocalization studies, cells were seeded and treated as described above. After compound incubation, cells were washed and stained with organelle-selective probes: ER-Tracker, MitoTracker, and LysoTracker (all from Thermo Fisher Scientific). Co-localization was quantified by calculating Pearson’s correlation coefficients using ImageJ.

### Analysis of Cell Death Mechanism

A549 cells were seeded in 6-well plates at a density of 1 × 10^5^ cells per well and incubated overnight. Cells were then treated for 18 h with iridium complexes at concentrations determined by their respective 72-h IC_50_ values under irradiating conditions. For irradiation experiments, samples were treated for a total of 2 h in Earle’s Balanced Salt Solution (EBSS), consisting of a 1-h dark incubation followed by 1 h of irradiation with 420 nm blue light. Following treatment, cells were incubated for 18 h in drug-free conditions before collection. Harvested cells were transferred to Annexin-V binding buffer and stained with Annexin-V Pacific Blue (Thermo Fisher Scientific) and Propidium Iodide (PI; Merck, 1 μg/mL). After a 15 min incubation at room temperature, samples were analyzed using a BD FACSVerse flow cytometer. Data analysis was performed using FCS Express 7 (DeNovo Software, CA).

### Mitochondrial Membrane Potential (Δψ_m_) Analysis

Mitochondrial membrane potential (Δψ_m_) was evaluated in A549 cells following photodynamic treatment with complexes **4**, **5**, and **7**. Cells were seeded in 6-well plates at a density of 1 × 10^5^ cells/well and incubated overnight under standard culture conditions. Subsequently, cells were treated with the test compounds at their respective IC_50_ concentrations for 2 h (comprising 1 h of dark incubation followed by 1 h of irradiation at 420 nm). Carbonyl cyanide 4-(trifluoromethoxy)­phenylhydrazone (FCCP, 10 μM) was used as a positive control, with a 30 min incubation prior to analysis. Following treatment, cells were stained with tetramethylrhodamine ethyl ester (TMRE, 200 nM). Samples were analyzed using a CellStream flow cytometer (Amnis), and data were processed using CellStream analysis software.

### Caspase 3/7 Activation

Caspase activation was evaluated to confirm the involvement of apoptotic pathways using the CellEvent Caspase-3/7 Green Detection Reagent (Thermo Fisher Scientific). A549 cells were seeded in 6-well plates at a density of 1 × 10^5^ cells per well and incubated overnight under standard culture conditions. Subsequently, cells were treated with the indicated compounds at their IC_50_ concentrations for 2 h (1 h dark incubation followed by 1 h irradiation at 405 nm). Staurosporine (1 μM, 4 h) was used as a positive control. Following treatment, cells were stained with the CellEvent Caspase-3/7 reagent prepared in PBS containing 5% FBS for 30 min in a humidified CO_2_ incubator. After staining, the cells were harvested and immediately analyzed using a CellStream flow cytometer (Amnis), and data were processed using CellStream analysis software.

### NADH Photo-Oxidation in Cells

A549 cells were harvested, and 1 × 10^5^ cells were used per sample. Cells were treated with candidate Ir compounds (**4**, **5**, and **7**) at their respective IC_50_ concentrations for 2 h, consisting of 1 h incubation in the dark followed by 1 h irradiation with blue light (420 nm, 58 W/m^2^). Intracellular NADH and NAD^+^ levels were quantified using the NAD^+^/NADH Assay Kit (Merck), according to the manufacturer’s instructions. The fluorescence of the reduced probe product was recorded at an excitation of 530 nm/emission of 585 nm. The NADH/NAD^+^ ratio was calculated for each condition. All experiments were performed in duplicate with triplicate samples per condition. Data are presented as mean ± SD.

### 3D Spheroid Culture

For three-dimensional (3D) spheroid experiments, 1000 single HCC827 cells per well were seeded into U-shaped 96-well plates with an ultralow attachment surface (Corning, USA). Spheroids were formed over 72 h in a specialized 3D spheroid-forming medium. This medium consisted of DMEM-F12 Ham medium supplemented with essential growth and spheroid-forming factors: 2% B27 (Thermo Fisher Scientific Inc., MA, USA), 20 ng/mL epidermal growth factor (EGF; Sigma-Aldrich, Germany), 10 ng/mL fibroblast growth factor 2 (FGF2; Sigma-Aldrich, Germany), and 0.15% bovine serum albumin (BSA).

### Distribution of Ir­(III) Compounds in Spheroids Analyzed by Confocal Microscopy

HCC827 cells were cultured for 72 h to form 3D spheroids in ULA 96-well U-shaped plates (Corning). Following spheroid formation, these 3D cultures were exposed to the candidate compound **4** at a concentration of 1 μM for 2 h. After treatment, the spheroids were gently rinsed, transferred to fresh 3D forming medium, and immediately visualized using a confocal microscope (Leica CM SP5, Leica, Germany). Images were acquired as defined Z-stacks with a 20 μm step size. Fluorescence intensities from these Z-stacks were captured immediately after the treatments and 72 h of incubation in drug-free medium and subsequently analyzed using ImageJ software.

### Metabolic Activity and Cell Death Detection within the Spheroids

Spheroids derived from HCC827 were treated for 2 h with the investigated compounds at a concentration of 1 μM. Then, the individual samples were irradiated for 60 s by three different wavelengths of laser light 405, 550, or 630 nm (1 mW), and placed in the drug-free, 3D forming medium for another 70 h. Then, treated and irradiated (or dark incubated controls) were stained with Hoechst 33258 (20 μg/mL), Calcein AM (2 μM), and PI (8 μg/mL) for 2 h. Samples were sequentially imaged on a confocal microscope, Leica CM SP5 (Leica, Germany), in 10 z-stack scans (20 μm each). Images were processed using ImageJ software. Fluorescence intensities were assessed for the maximal projections of the z-stacks, and the corresponding Calcein AM/PI ratio was calculated. Hoechst dye was used to accurately define the Region of Interest (ROI) mask and analyze fluorescence intensities. Three independent experiments were carried out to ensure the reliability and reproducibility of the results.

## Supplementary Material





## Notes

Scheme of ancillary Ligand (**L** = tfbbztbzim) and intermediates **A** and **B**, proligands **HC^N6** (Htfbpbzim) and **HC^N7** (Htfbnapbzim), iridium dimers **D1**–**D7**, and iridium complexes **1**–**7**; characterization, analytical and photophysical data, the photochemical evaluation, the photo­(cytotoxic) experiments under both 2D and 3D conditions and microscopy experiments (PDF)

## Abbreviations used

ACQ: aggregation-caused quenching
AIE: aggregation-induced emission
CAM: calcein AM
CCDC: Cambridge crystallographic data center
DHR123: dehydrorhodamine 123
DMEM: Dulbecco’s Modified Eagle Medium
DLS: dynamic light scattering
DMF: dimethylformamide
DMSO: dimethyl sulfoxide
DPBF: 1,3-diphenylisobenzofuran
EBSS: Earle’s balanced salt solution
EGFR: epidermal growth factor receptor
ER: endoplasmic reticulum
ETC: electron transport chain
FBS: fetal bovine serum
Hbzq: 7,8-benzoquinoline
HOMO: highest occupied molecular orbital
Hpbo: 2-phenylbenzoxazole
Hpbt: 2-phenylbenzothiazole
HPF: hydroxyphenyl fluorescein
HPLC: high performance liquid chromatography
Hppy: 2-phenylpyridine
HR-ESI-MS: high-resolution electrospray ionization—mass spectrometry
HSA: human serum albumin
Htfbnapbzim: 2-(naphthalen-2-yl)-1-(4-(trifluoromethyl)­benzyl)­benzimidazole
Htfbpbzim: 2-pheny-1-(4-(trifluoromethyl)­benzyl)­benzimidazole
Hthpy: 2-(thiophen-2-yl)­pyridine
ICP-MS: inductively coupled plasma-mass spectrometry
ISC: intersystem crossing
LC: ligand-centered
LLCT: ligand-to-ligand charge transfer
LUMO: lowest unoccupied molecular orbital
MLCT: metal-to-ligand charge transfer
MTT: 3-(4,5-dimethylthiazol-2-yl)-2,5-diphenyltetrazolium bromide
NADH: nicotinamide adenine dinucleotide hydrogen
NMR: nuclear magnetic resonance
NSCLC: non-small cell lung cancer
NTA: Nanoparticle Tracking analysis
PCC: Pearson’s correlation coefficient
PDT: photodynamic therapy
PI: phototoxicity index
PI: propidium iodide
PS: photosensitizer
ROS: reactive oxygen species
SET: single electron transfer
SOC: spin–orbit coupling
TCA: tricarboxylic acid
TICT: twisted intramolecular charge transfer
TOF: turnover frequency
UV/vis: ultraviolet/visible.
