# Supplementary material for: Novel 2‑(2′-Benzothiazolyl)-benzimidazole-Based Iridium(III) Photocatalysts Exhibit Antiproliferative Effects in 2D and 3D Cancer Cells to Bypass Hypoxia-Induced Resistance
Source: J Med Chem. 2026 Mar 16;69(6):6991–7011. doi: 10.1021/acs.jmedchem.5c03280 (PMC13036781; doi:10.1021/acs.jmedchem.5c03280)
Supplement: Supplementary file 1 [file jm5c03280_si_001.pdf]

Supporting Information

Novel 2-(2'-benzothiazolyl)-benzimidazole-based iridium(III) photocatalysts exhibit antiproliferative effects in 2D and 3D cancer cells to bypass hypoxia-induced resistance

Antonio Linero-Artiaga,[a,#] Marie Svitelova,[b,c,#] Vojtěch Novohradský,[b] Venancio Rodríguez,[a] Lenka Markova,[b] Jana Kasparkova,[c] Christoph Janiak,[d] José Ruiz,[a,*] Viktor Brabec[c,*]

a Departamento de Química Inorgánica, Universidad de Murcia, and Murcia BioHealth Research Institute (IMIB-Arrixaca), E-30071 Murcia, Spain

b Czech Academy of Sciences, Institute of Biophysics, Kralovopolska 135, CZ-61200 Brno, Czech Republic

c Department of Molecular Pharmacy, Faculty of Pharmacy, Masaryk University, Palackeho trida 1946/1, CZ-61200 Brno, Czech Republic

d Institut für Anorganische Chemie und Strukturchemie, Heinrich-Heine-Universität Düsseldorf, D-40204 Düsseldorf, Germany

e Department of Biophysics, Faculty of Science, Palacky University, Slechtitelu 27, CZ-78371 Olomouc, Czech Republic

* Corresponding authors J.R.: jruiz@um.es , V.B.: viktor.brabec@upol.cz

TABLE OF CONTENTS

1. SUPPORTING FIGURES AND TABLES S6

Figure S1. ^{1}H-NMR spectrum of proligand **HC^N6** (DMSO-d_6, 300 MHz). S6

Figure S2. ^{1}H-NMR spectrum of proligand **HC^N7** (DMSO-d_6, 400 MHz). S6

Figure S3. ^{1}H-NMR spectrum of ancillary ligand **L** ($CDCl_3$, 400 MHz). S7

Figure S4. ^{13}C-NMR spectrum of ancillary ligand **L** ($CDCl_3$, 101 MHz). S7

Figure S5. ^{19}F{^{1}H}-NMR spectrum of ancillary ligand **L** ($CDCl_3$, 377 MHz). S8

Figure S6. HR-ESI-MS spectrum of ancillary ligand **L** ($CHCl_3$). S8

Figure S7. Picture of the pure powder obtained for complexes **1-7**. S8

Figure S8. ^{1}H-NMR spectrum of complex **1** (DMSO-d_6, 600 MHz). S9

Figure S9. ^{13}C-NMR spectrum of complex **1** (DMSO-d_6, 151 MHz). S9

Figure S10. ^{19}F{^{1}H}-NMR spectrum of complex **1** (DMSO-d_6, 377 MHz). S10

Figure S11. ^{31}P-NMR spectrum of complex **1** (DMSO-d_6, 121 MHz). S10

Figure S12. ^{1}H-NMR spectrum of complex **2** (DMSO-d_6, 600 MHz). S11

Figure S13. ^{13}C-NMR spectrum of complex **2** (DMSO-d_6, 151 MHz). S11

Figure S14. ^{19}F{^{1}H}-NMR spectrum of complex **2** (DMSO-d_6, 377 MHz). S12

Figure S15. ^{31}P-NMR spectrum of complex **2** (DMSO-d_6, 162 MHz). S12

Figure S16. ^{1}H-NMR spectrum of complex **3** (DMSO-d_6, 600 MHz). S13

Figure S17. ^{13}C-NMR spectrum of complex **3** (DMSO-d_6, 151 MHz). S13

Figure S18. ^{19}F{^{1}H}-NMR spectrum of complex **3** (DMSO-d_6, 377 MHz). S14

Figure S19. ^{31}P-NMR spectrum of complex **3** (DMSO-d_6, 121 MHz). S14

Figure S20. ^{1}H-NMR spectrum of complex **4** (DMSO-d_6, 600 MHz). S15

Figure S21. ^{13}C-NMR spectrum of complex **4** (DMSO-d_6, 151 MHz). S15

Figure S22. ^{19}F{^{1}H}-NMR spectrum of complex **4** (DMSO-d_6, 377 MHz). S16

Figure S23. ^{31}P-NMR spectrum of complex **4** (DMSO-d_6, 162 MHz). S16

Figure S24. ^{1}H-NMR spectrum of complex **5** (DMSO-d_6, 600 MHz). S17

Figure S25. ^{13}C-NMR spectrum of complex **5** (DMSO-d_6, 151 MHz). S17

Figure S26. ^{19}F{^{1}H}-NMR spectrum of complex **5** (DMSO-d_6, 377 MHz). S18

Figure S27. ^{31}P-NMR spectrum of complex **5** (DMSO-d_6, 121 MHz). S18

Figure S28. ^{1}H-NMR spectrum of complex **6** (DMSO-d_6, 600 MHz). S19

Figure S29. ^{13}C-NMR spectrum of complex **6** (DMSO-d_6, 151 MHz). S19

Figure S30. ^{19}F{^{1}H}-NMR spectrum of complex **6** (DMSO-d_6, 377 MHz). S20

Figure S31. ^{31}P-NMR spectrum of complex **6** (DMSO-d_6, 121 MHz). S20

Figure S32. ^{1}H-NMR spectrum of complex **7** (DMSO-d_6, 600 MHz). S21

Figure S33. ^{13}C-NMR spectrum of complex **7** (DMSO-d_6, 151 MHz). S21

Figure S34. ^{19}F{^{1}H}-NMR spectrum of complex **7** (DMSO-d_6, 377 MHz). S22

Figure S35. ^{31}P-NMR spectrum of complex **7** (DMSO-d_6, 121 MHz). S22

Figure S36. HR-ESI-MS spectra of complexes **1-7** (CH_3CN). S23

Figure S37. RP-HPLC chromatograms of complexes **1-7** (CH_3CN). S24

Table S1. Crystal data and structure refinement details for complex **2**. S25

Table S2. Selected bond distances (Å) and bond angles (°) for complex **2** referring to the atom numbering in the image below: S26

Scheme S1. Graphical presentation of the parameters used for the description of π-π stacking. S27

Scheme S2. Graphical presentation of the parameters used for the description of CH-π interactions.[6] S28

Table S3. Packing Analysis for complex **2** for possible π···π interactions (see **Scheme S1** for explanation). S28

Figure S38. π-π and part of the C-H···π interactions in the packing of complex **2** in the crystal, indicated as dashed black lines for π-π and dashed orange lines for C-H···π with the centroid-centroid contact given in Å. Further details of these π-π and C-H···π interactions, including the symmetry transformations are listed in the above **Tables S3** and **S4** (Cg = ring centroid). H atoms are not shown for clarity. ...S29

Table S4. Analysis of *inter*molecular C-H···Cg(Pi-Ring) Interactions (H···Cg < 3.0 Ang. - Gamma < 30.0 Deg) in complex **2** (see **Scheme S2** for explanation). S29

Table S5. Analysis of Potential Hydrogen Bonds and Schemes with d(D···A) < R(D)+R(A)+0.50, d(H···A) < R(H)+R(A)-0.12 Ang., D-H···A > 100.0 Deg S30

Figure S39. C-H···F contacts in the packing of complex **2** in the crystal, indicated as dashed orange lines. Further details of these C-H···F interactions, including the symmetry transformations are listed in the above **Table S5**. S30

Table S6. Crystal data and structure refinement details for complex **5**. S31

Table S7. Selected bond distances (Å) and bond angles (°) for complex **5** referring to the atom numbering in the image below: S32

Scheme S3. Graphical presentation of the parameters used for the description of π-π stacking. S33

Scheme S4. Graphical presentation of the parameters used for the description of CH-π interactions.[6] S34

Table S8. Packing Analysis for complex **5** for possible π···π interactions (see **Scheme S4** for explanation). S34

Figure S40. π-π and part of the C-H···π interactions in the packing of complex **5** in the crystal, indicated as dashed black lines for π-π and dashed orange lines for C-H···π with the centroid-centroid contact given in Å. Further details of these π-π and C-H···π interactions, including the symmetry transformations are listed in the above **Tables S8** and **S9** (Cg = ring centroid). H atoms are not shown for clarity. ...S35

Table S9. Analysis of *inter*molecular C-H...Cg(Pi-Ring) Interactions (H..Cg < 3.0 Ang. - Gamma < 30.0 Deg) in complex **5** (see **Scheme S4** for explanation). S36

Figure S41. Monitoring of the UV/VIS absorption spectra of complexes **1-6** (10 µM) in DMSO after incubation at room temperature for 48 h. S37

Figure S42. Monitoring of the UV/VIS absorption spectra of complexes **1-6** (10 µM) in a DMEM (+10% FBS)/DMSO (95:5) mixture before and after (t = 48 h) incubation at 37 ºC. S38

Figure S43. Picture of the glass vials containing solutions of complexes **1-7** (10 µM) in a DMEM (+10% FBS)/DMSO (95:5) mixture (48 h incubation at 37 ºC). S38

Figure S44. Stability of complex **1** (10 µM) in a DMEM (+10% FBS)/DMSO (95:5) mixture recorded by HPLC/HR-ESI-MS. S39

Figure S45. Stability of complex **2** (10 µM) in a DMEM (+10% FBS)/DMSO (95:5) mixture recorded by HPLC/HR-ESI-MS. S40

Figure S46. Stability of complex **3** (10 µM) in a DMEM (+10% FBS)/DMSO (95:5) mixture recorded by HPLC/HR-ESI-MS. S41

Figure S47. Stability of complex **4** (10 µM) in a DMEM (+10% FBS)/DMSO (95:5) mixture recorded by HPLC/HR-ESI-MS. S42

Figure S48. Stability of complex **5** (10 μM) in a DMEM (+10% FBS)/DMSO (95:5) mixture recorded by HPLC/HR-ESI-MS. S43

Figure S49. Stability of complex **6** (10 μM) in a DMEM (+10% FBS)/DMSO (95:5) mixture recorded by HPLC/HR-ESI-MS. S44

Figure S50. Tyndall effect observed in cuvettes containing solutions of **1-7** (10 μM) in a DMEM (+10% FBS)/DMSO (95:5) mixture at t = 0 and after 48 h of incubation at 37 ºC. S44

Figure S51. UV/VIS absorption spectra of complexes **1-7** (10 μM) recorded in acetonitrile. S45

Figure S52. Emission spectra of complexes **1-7** (10 μM) recorded in CH_3CN. λ_{irrad} = 420 nm S46

Figure S53. Emission spectra of complexes **1-7** (10 μM) recorded in a H_2O/DMSO (99:1) mixture. λ_{irrad} = 420 nm. S46

Table S11. Photophysical characterization of complexes **1-7** (10 μM) in acetonitrile and water (1% DMSO). S47

Figure S54. Monitoring of the emission spectra (λ_{irrad} = 420 nm) of complexes **1-7** (10 μM) while increasing the water fraction. Pictures of the cuvettes containing the aqueous solutions upon light irradiation with a short-wavelength lamp. S48

Figure S55. Photostability of complexes **1-7** (10 μM) in DMSO monitored by UV/VIS absorption spectroscopy. Samples were irradiated for 1 h with a blue (λ_{irrad} = 465 nm, 5 mW/cm^2) lamp. S49

Figure S56. Absorption profile of 1,3-diphenylisobenzofuran after incubation with $[Ru(2,2'\text{-bipyridine})_3]^{2+}$ (5 μM) and complexes **1-7** (30-50 μM) and exposure to blue (λ_{irrad} = 465 nm, 0.4 mW/cm^2) light irradiation for 15 second intervals. S50

Figure S57. Time-dependent monitoring of the fluorescence spectra of HPF (10 μM) upon incubation with complexes **1-7** (10 μM) in a H_2O/DMF (95:5) mixture and irradiation with a blue lamp (λ_{irrad} = 465 nm, 5 mW/cm^2) for short intervals. S51

Figure S58. Time-dependent monitoring of the fluorescence spectra of DHR123 (10 μM) upon incubation with complexes **1-7** (10 μM) in a H_2O/DMF (95:5) mixture and irradiation with a blue lamp (λ_{irrad} = 465 nm, 5 mW/cm^2) for short intervals. S52

Figure S59. Time-dependent monitoring of the fluorescence spectra of Amplex Red (10 μM) upon incubation with complexes **1-7** (5 μM) in a H_2O/DMF (95:5) mixture and irradiation with a blue lamp (λ_{irrad} = 465 nm, 5 mW/cm^2) for short intervals. S53

Figure S60. Hydrogen peroxide strips dipped into irradiated (λ_{irrad} = 465 nm, 5 mW/cm^2, 1 h) solutions of relevant biomolecules (1 mM) in the presence of complexes **1-7** (10 μM) in a H_2O/DMF (95:5) mixture. S54

Figure S61. Monitoring of the UV/VIS absorption profile of NADH (100 μM) upon incubation with complexes **1-6** (5 μM) in a H_2O/DMF (95:5) mixture and exposure to blue light irradiation (λ_{irrad} = 465 nm, 5 mW/cm^2, 10-15 min). S54

Figure S62. Monitoring of the absorption profile of NADH (100 μM) upon incubation for 45 min with complexes **1-7** (5 μM) in a H_2O/DMF (95:5) mixture in the dark. S55

Table S12. Singlet oxygen production quantum yield (Φ_Δ) and NADH photooxidation TOF (h^{-1}) values obtained for complexes **1-7**. S55

Figure S63. Monitoring of the UV/VIS absorption profile of NADH (100 μM) upon incubation with complexes **1-7** (5 μM) and tiron (10 mM) in a H_2O/DMF (95:5) mixture and exposure to blue light irradiation (λ_{irrad} = 465 nm, 5 mW/cm^2, 10-15 min). S56

Figure S64. Monitoring of the UV/VIS absorption profile of NADH (100 μM) upon incubation with complexes **1-7** (5 μM) and mannitol (20 mM) in a H_2O/DMF (95:5) mixture and exposure to blue light irradiation (λ_{irrad} = 465 nm, 5 mW/cm^2, 10-15 min). S57

Figure S65. Monitoring of the UV/VIS absorption profile of NADH (100 μM) upon incubation with complexes **1-7** (5 μM) and sodium pyruvate (10 mM) in a H_2O/DMF (95:5) mixture and exposure to blue light irradiation (λ_{irrad} = 465 nm, 5 mW/cm^2, 10-15 min). S58

Figure S66. Monitoring of the UV/VIS absorption profile of NADH (100 µM) upon incubation with complexes **1-7** (5 µM) and trolox (0.1 mM) in a H_2O/DMF (95:5) mixture and exposure to blue light irradiation (λ_{irrad} = 465 nm, 5 mW/cm^2, 10-15 min). S59

Figure S67. Monitoring of the UV/VIS absorption profile of NADH (100 µM) upon incubation with complexes **1-7** (5 µM) in a dearated H_2O/DMF (95:5) mixture and exposure to blue light irradiation (λ_{irrad} = 465 nm, 5 mW/cm^2, 10-15 min). S60

Table S13. The antiproliferative activity (IC_{50} values[a]) of the investigated complexes determined in A549 cells under hypoxic conditions (1% O_2).[b,c] S60

Figure S68. Localization of tested compounds in A549 cells using confocal microscopy. Panels show: A) Fluorescence channel indicating compound localization; B) Bright-field channel; C) Overlay of fluorescence and bright-field channels. Complexes **4** (Panel 1), **5** (Panel 2), and **7** (Panel 3) were tested. A549 cells were treated for 2 h with 5 µM of each compound. Scale bars represent 20 µm. S61

Figure S69. Investigation of cell death mechanisms in A549 cells following photodynamic treatment. A549 cells were exposed to compounds at their IC_{50} concentrations for a total of 2 h, comprising a 1-h dark incubation followed by 1 h of 420 nm blue light irradiation. The mode of cell death was subsequently assessed 18 h post-treatment via flow cytometry, employing propidium iodide to detect necrosis and Annexin V to detect apoptosis S62

Figure S70. Photodynamic treatment induces mitochondrial membrane potential ($\Delta\psi_m$) loss in A549 cells. (A) Quantitative analysis of TMRE fluorescence intensity. (B) Representative flow cytometry histograms showing MMP distribution. Cells were treated with compounds at IC_{50} concentrations for 2 h (1 h dark incubation followed by 1 h of 420 nm light irradiation). MMP was assessed 18 h post-treatment using TMRE staining. FCCP was employed as a positive control for mitochondrial depolarization. Data represent the mean ±SD of three independent experiments performed in duplicate. ***$p \leq 0.001$ compared to treated, dark incubated cells. S63

Figure S71. Caspase-3/7 activation in A549 cells following photodynamic treatment. (A) Quantitative analysis of fluorescence intensity using the CellEvent™ Caspase-3/7 Green Detection Reagent. (B) Representative flow cytometry histograms showing fluorescence intensity shifts indicative of caspase-3/7 activation. A549 cells were treated with the indicated compounds at their IC_{50} concentrations for 2 h (1 h dark incubation followed by 1 h irradiation at 420 nm). Staurosporine (1 µM, 4 h) served as a positive control. Data represent the mean ± SD of three independent experiments performed in duplicate. ***$p \leq 0.001$ compared to treated, dark-incubated cells. S63

Figure S72. Penetration of compound 4 to HCT-116 spheroids. Z-stack images were acquired by using confocal microscopy. Scale bar represents 200 µm. The graph depicts mean fluorescence intensity from the periphery to the core of spheroids. Error bars represent the standard deviation from two independent experiments S64

2. REFERENCES S64

1. SUPPORTING FIGURES AND TABLES

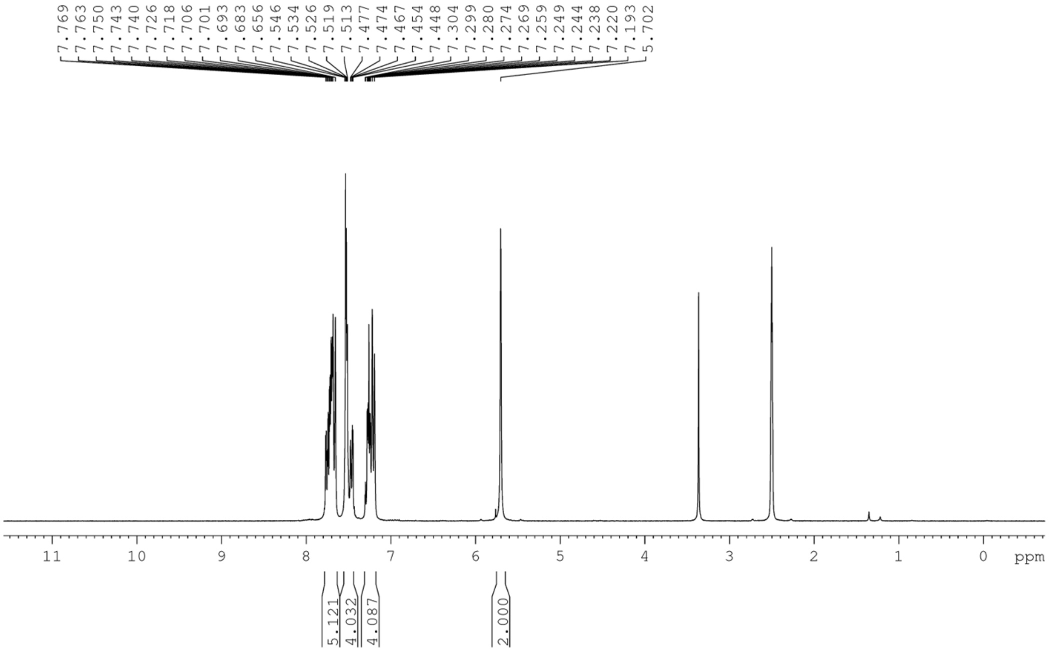

Figure S1. ^{1}H-NMR spectrum of proligand **HC^N6** (DMSO-d_6, 300 MHz).

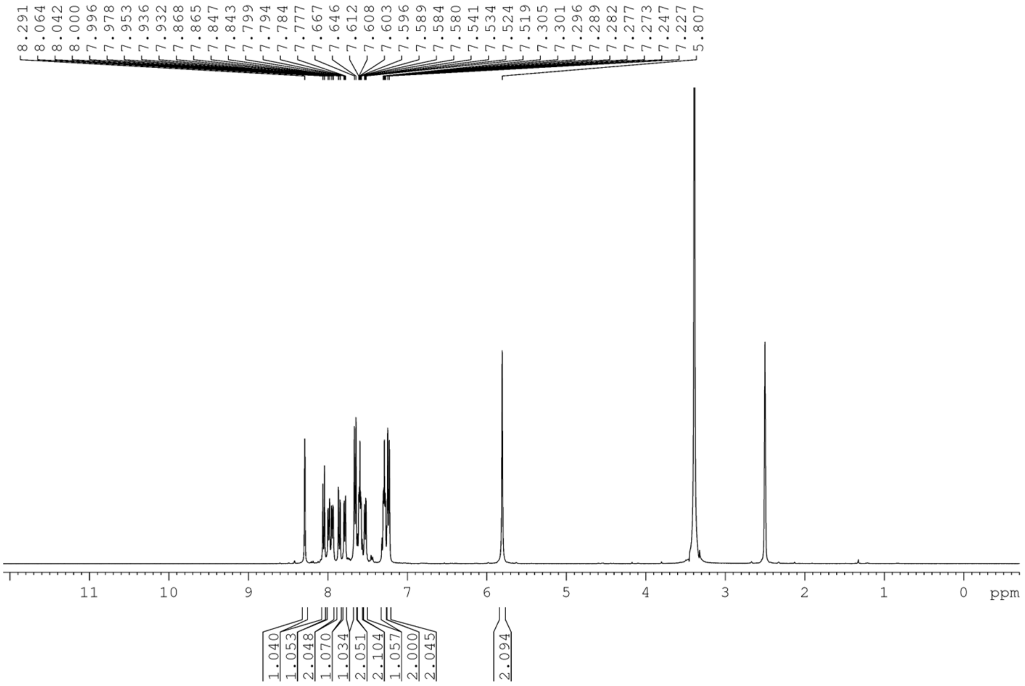

Figure S2. ^{1}H-NMR spectrum of proligand **HC^N7** (DMSO-d_6, 400 MHz).

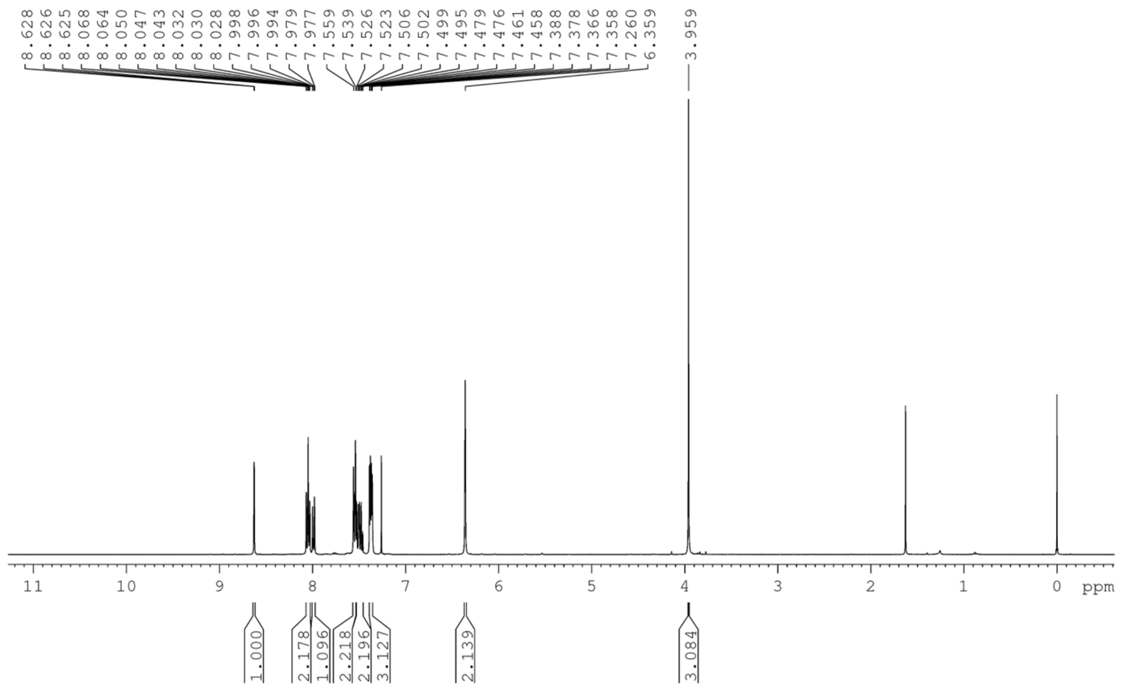

Figure S3. ^{1}H-NMR spectrum of ancillary ligand **L** ($CDCl_3$, 400 MHz).

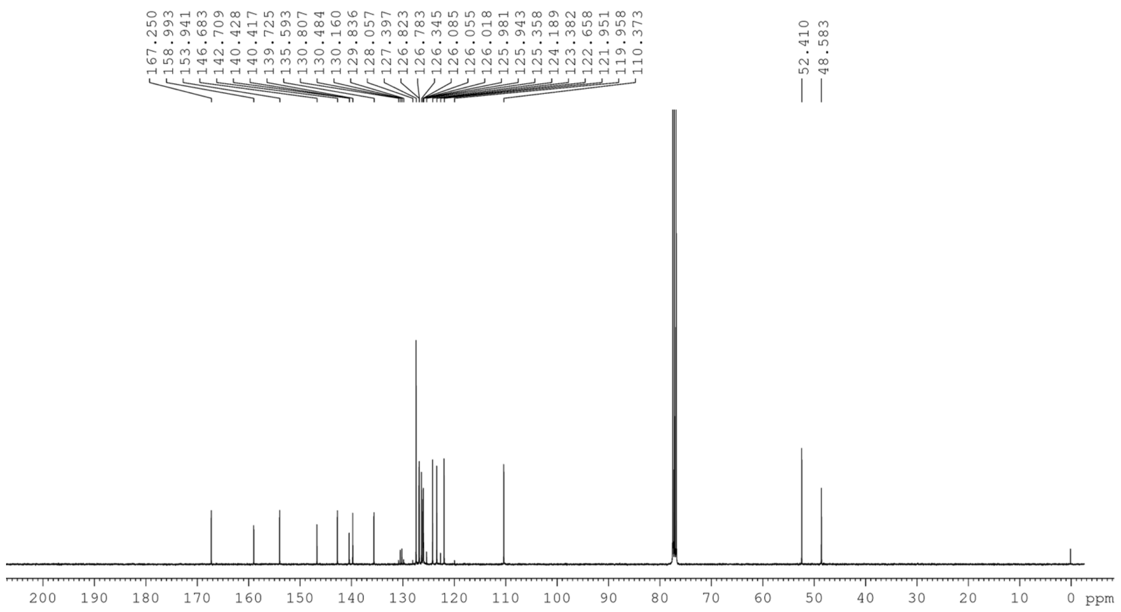

Figure S4. ^{13}C-NMR spectrum of ancillary ligand **L** ($CDCl_3$, 101 MHz).

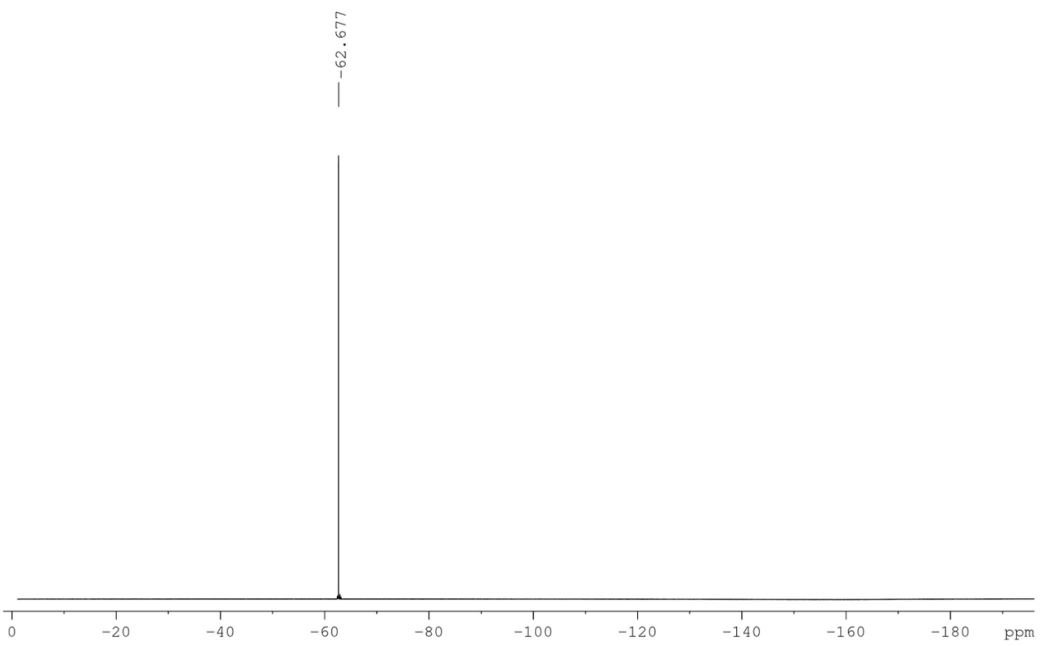

Figure S5. $^{19}F\{^{1}H\}$-NMR spectrum of ancillary ligand **L** ($CDCl_3$, 377 MHz).

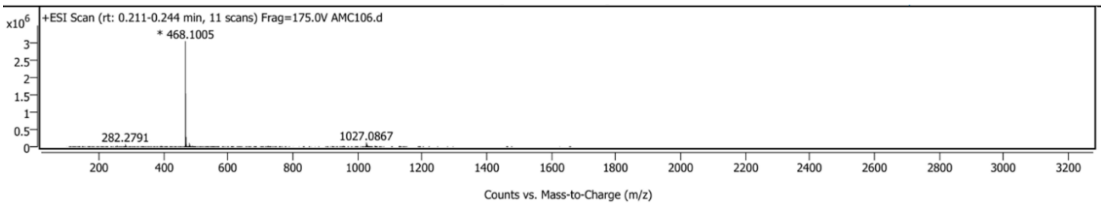

Figure S6. HR-ESI-MS spectrum of ancillary ligand **L** ($CHCl_3$).

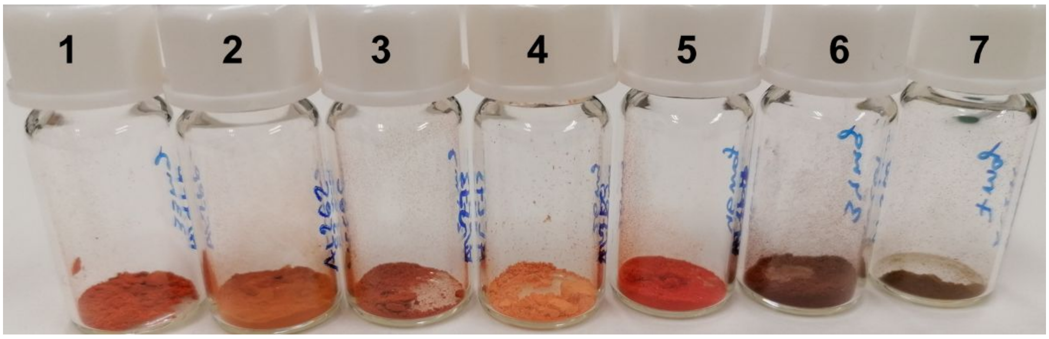

Figure S7. Picture of the pure powder obtained for complexes **1-7**.

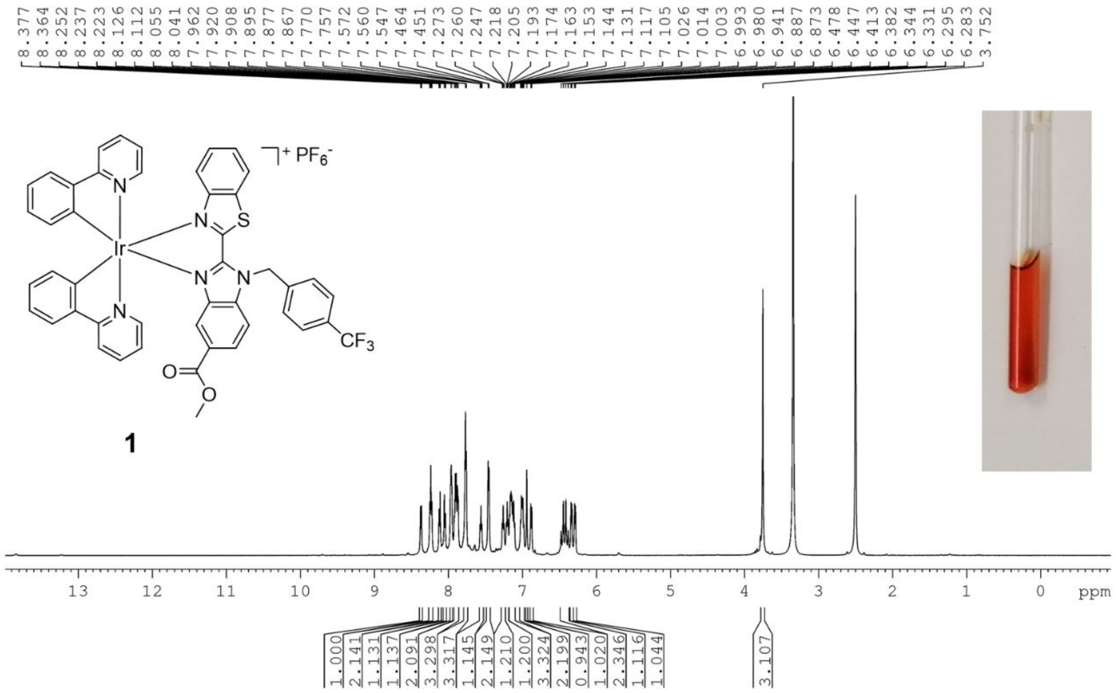

Figure S8. ^{1}H-NMR spectrum of complex **1** (DMSO-d_6, 600 MHz).

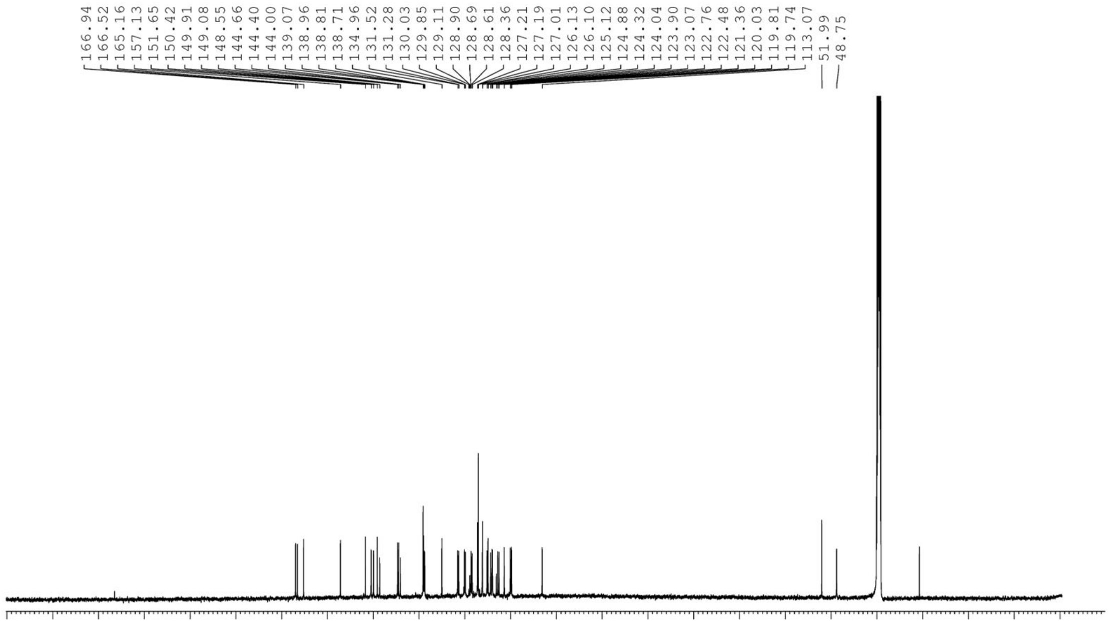

Figure S9. ^{13}C-NMR spectrum of complex **1** (DMSO-d_6, 151 MHz).

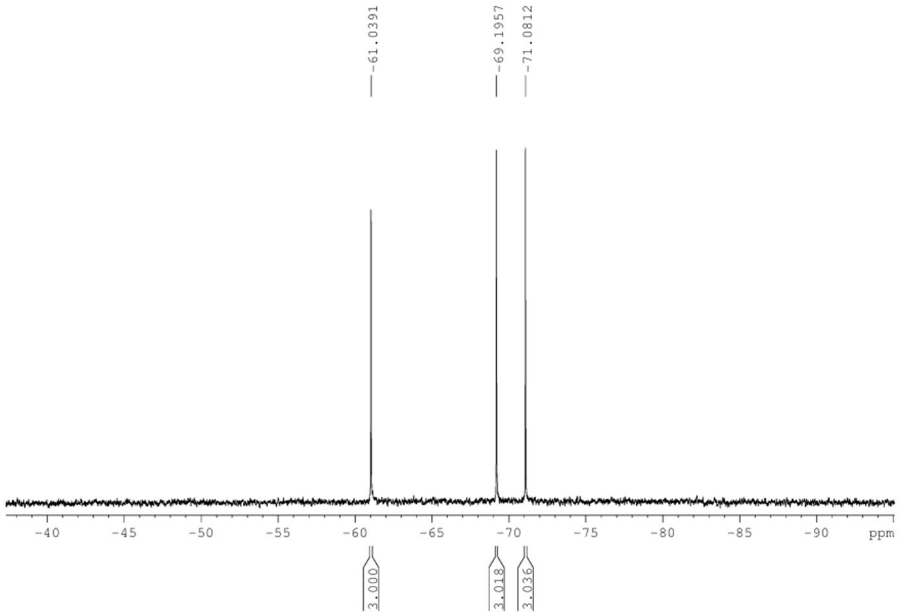

Figure S10. $^{19}F\{^{1}H\}$-NMR spectrum of complex **1** (DMSO-d_6, 377 MHz).

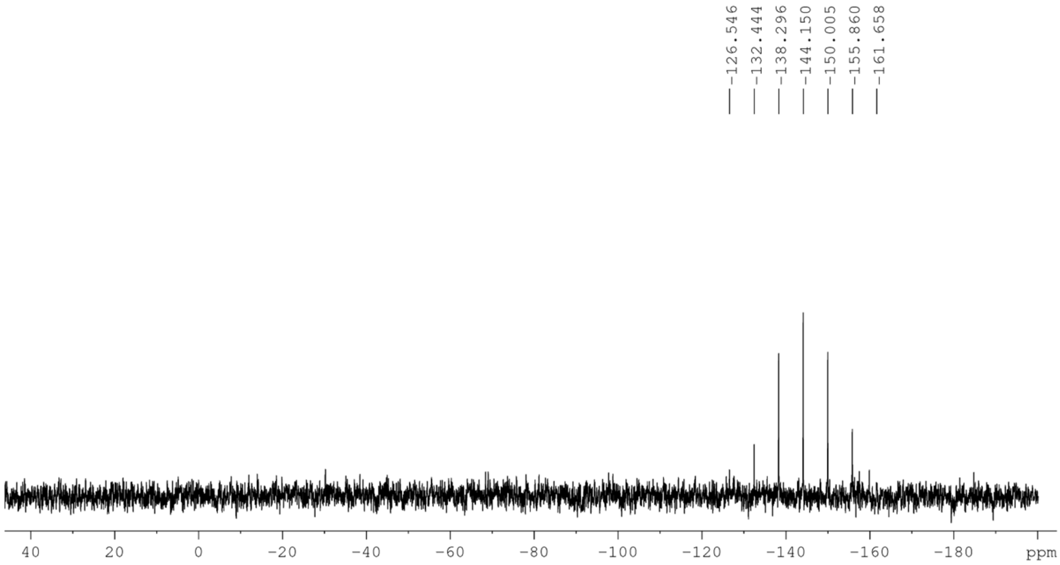

Figure S11. ^{31}P-NMR spectrum of complex **1** (DMSO-d_6, 121 MHz).

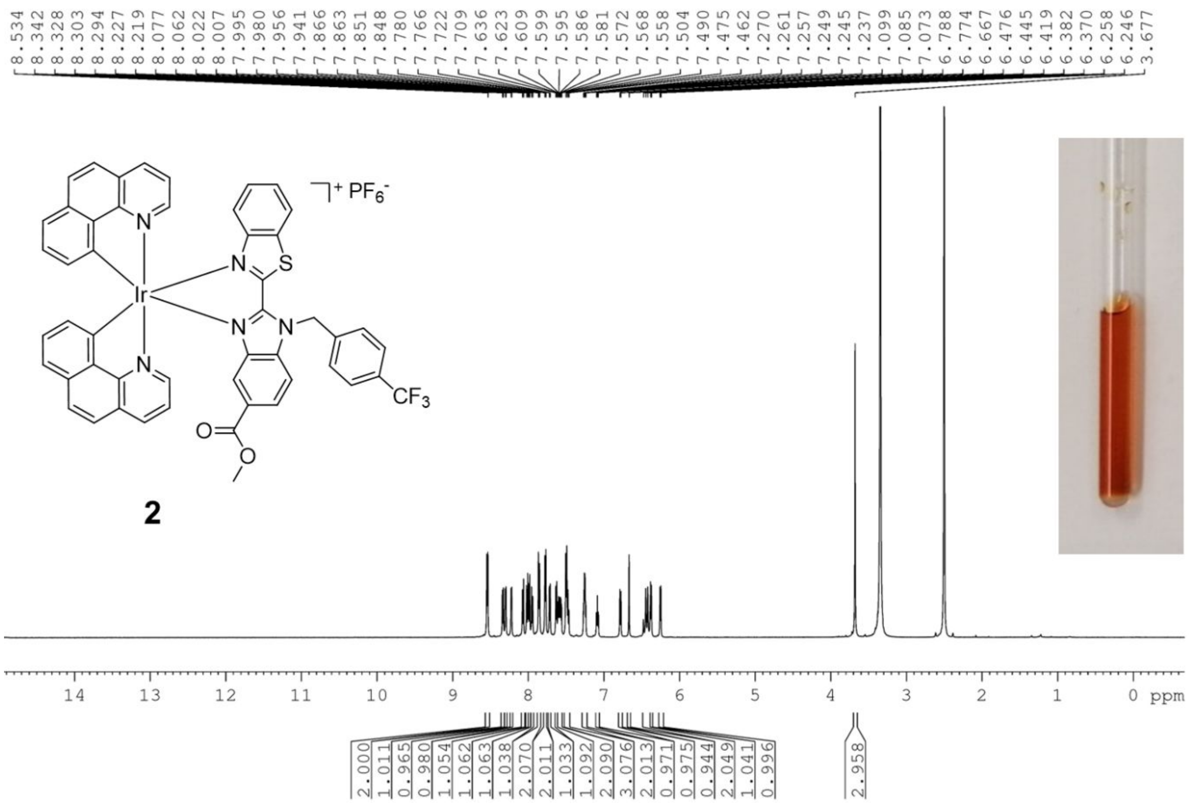

Figure S12. ^{1}H-NMR spectrum of complex **2** (DMSO-d_6, 600 MHz).

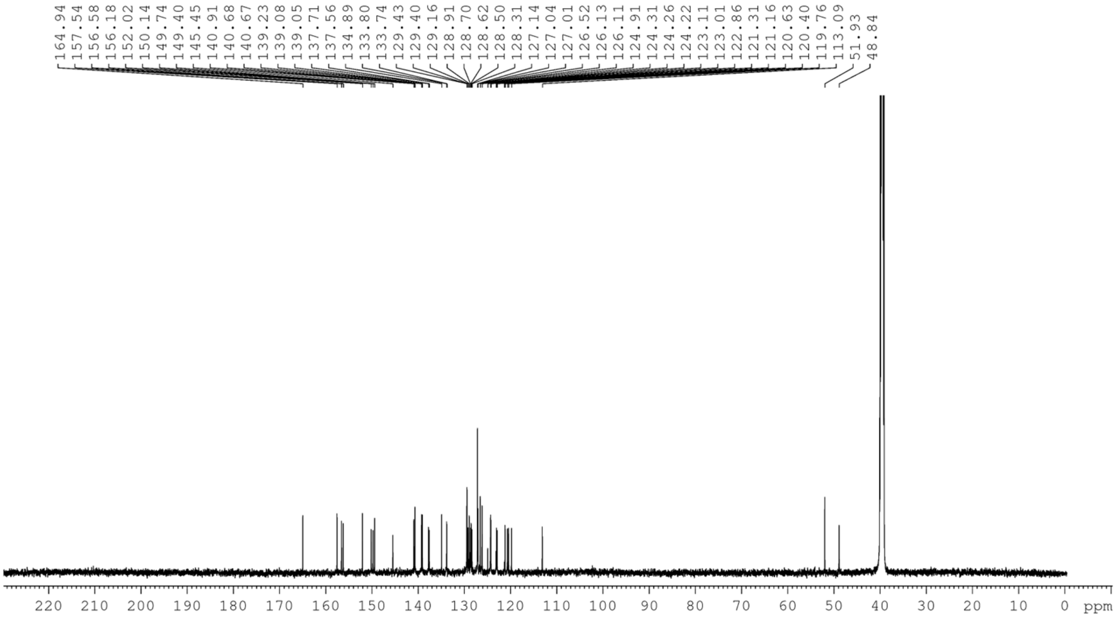

Figure S13. ^{13}C-NMR spectrum of complex **2** (DMSO-d_6, 151 MHz).

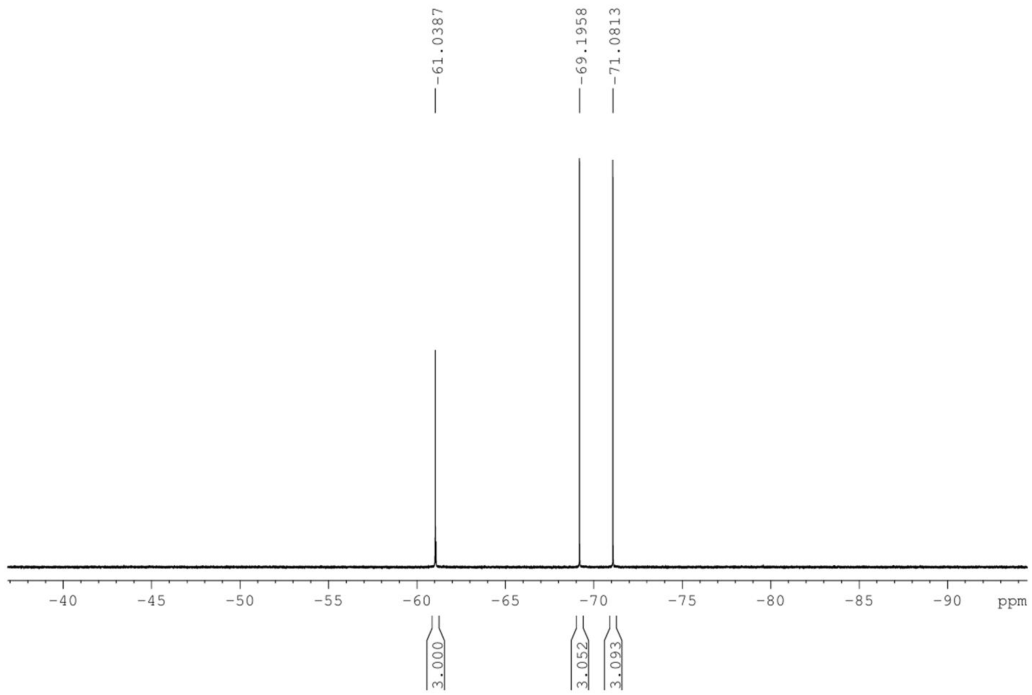

Figure S14. $^{19}F\{^{1}H\}$-NMR spectrum of complex **2** (DMSO-d_6, 377 MHz).

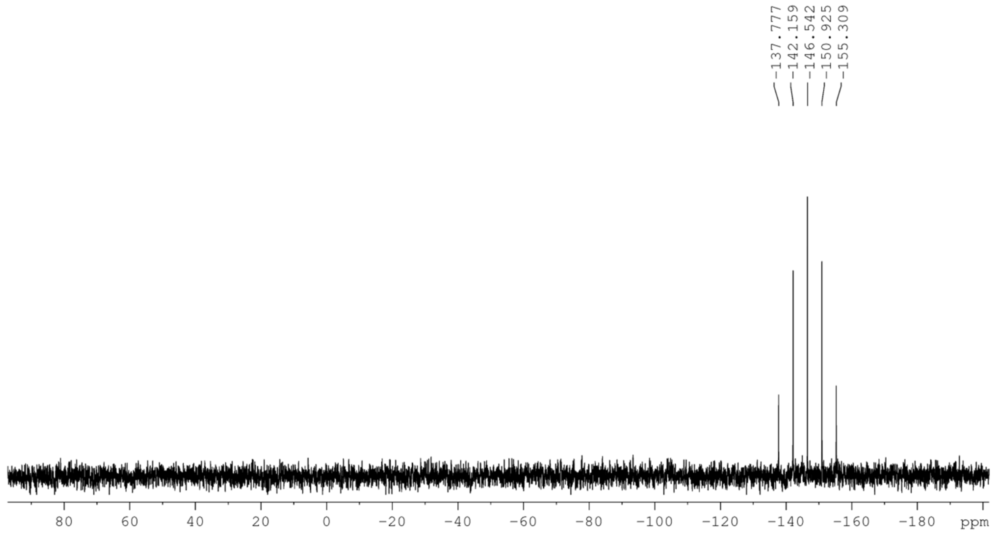

Figure S15. ^{31}P-NMR spectrum of complex **2** (DMSO-d_6, 162 MHz).

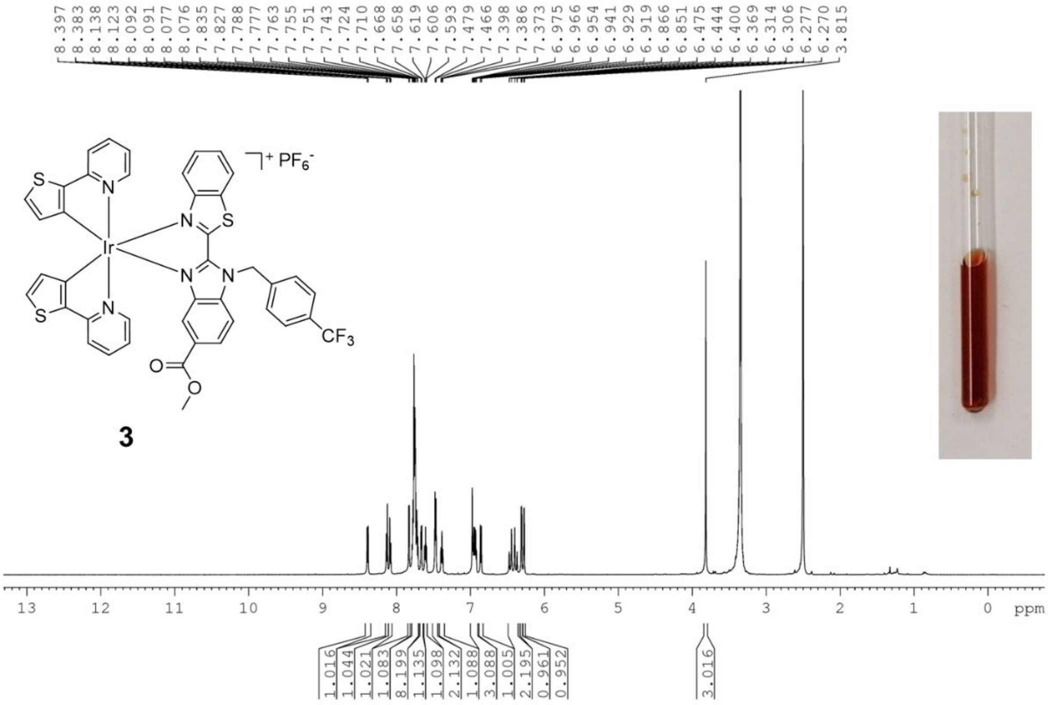

Figure S16. ^{1}H-NMR spectrum of complex **3** (DMSO-d_6, 600 MHz).

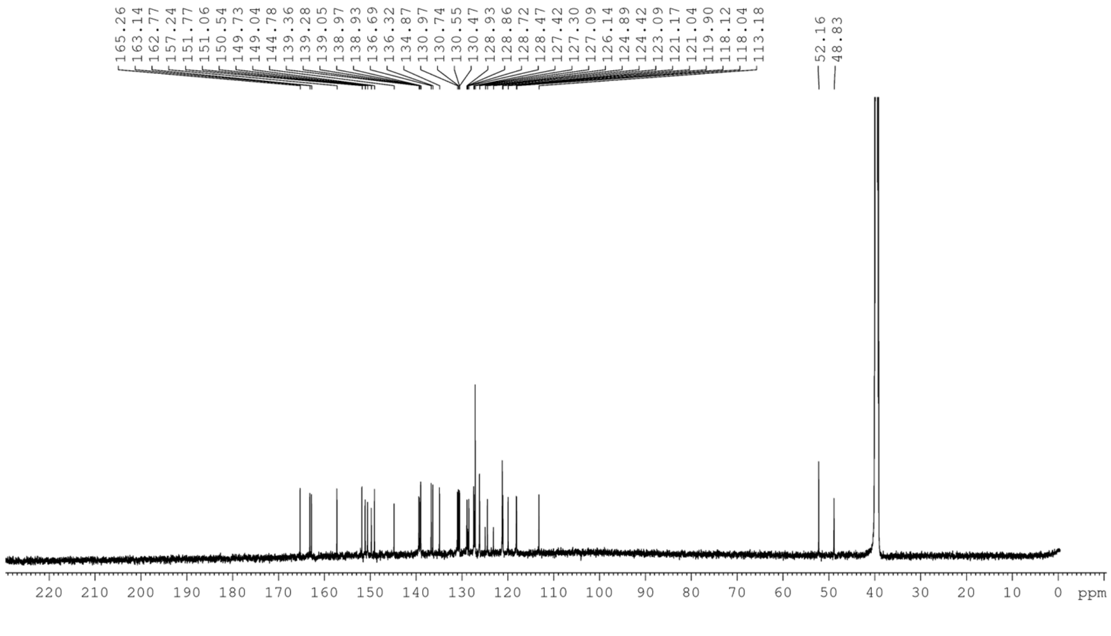

Figure S17. ^{13}C-NMR spectrum of complex **3** (DMSO-d_6, 151 MHz).

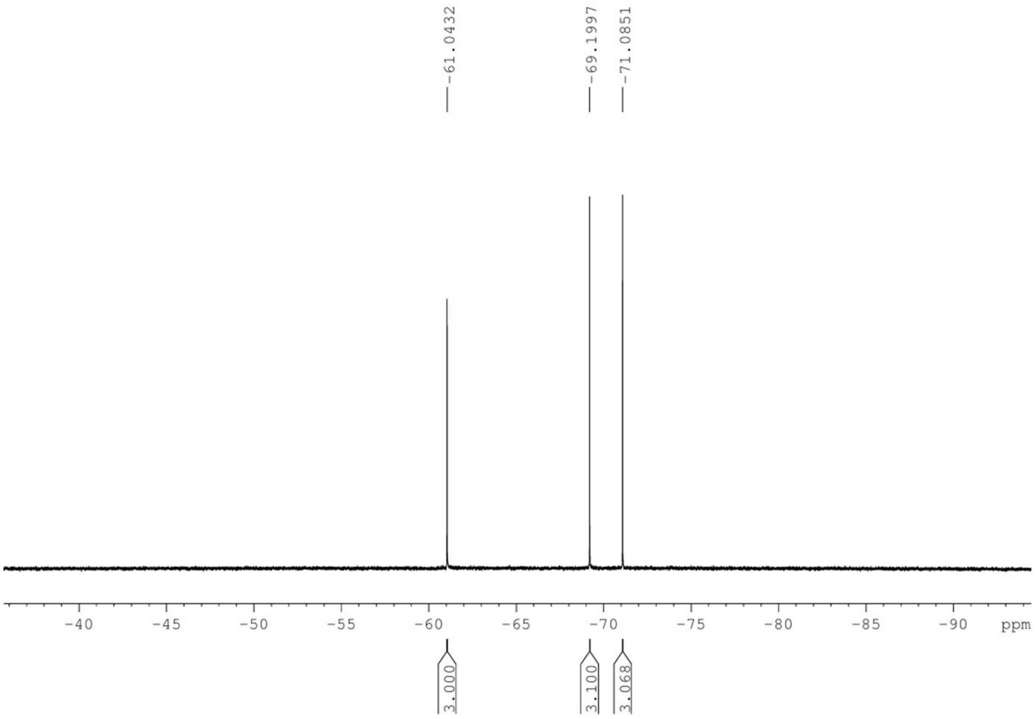

Figure S18. $^{19}F\{^{1}H\}$-NMR spectrum of complex **3** (DMSO-d_6, 377 MHz).

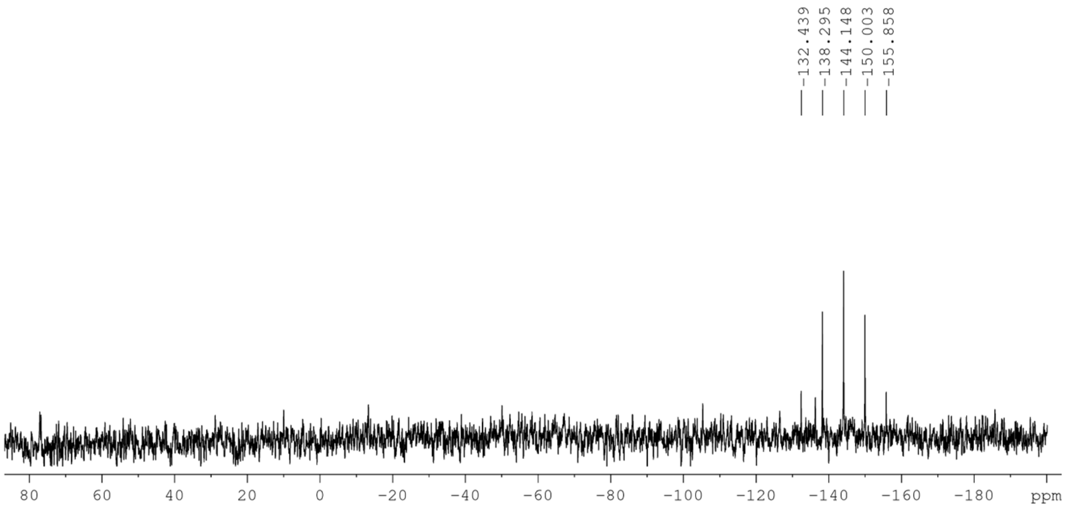

Figure S19. ^{31}P-NMR spectrum of complex **3** (DMSO-d_6, 121 MHz).

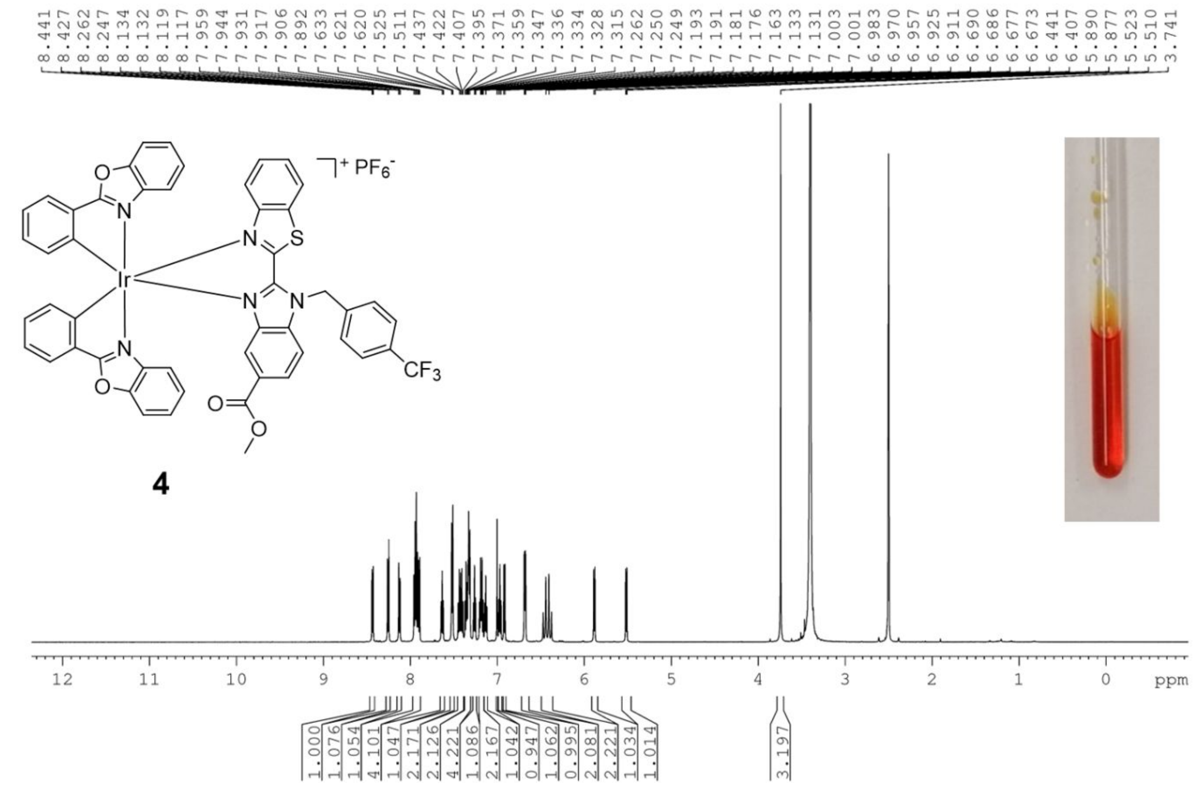

Figure S20. ^{1}H-NMR spectrum of complex **4** (DMSO-d_6, 600 MHz).

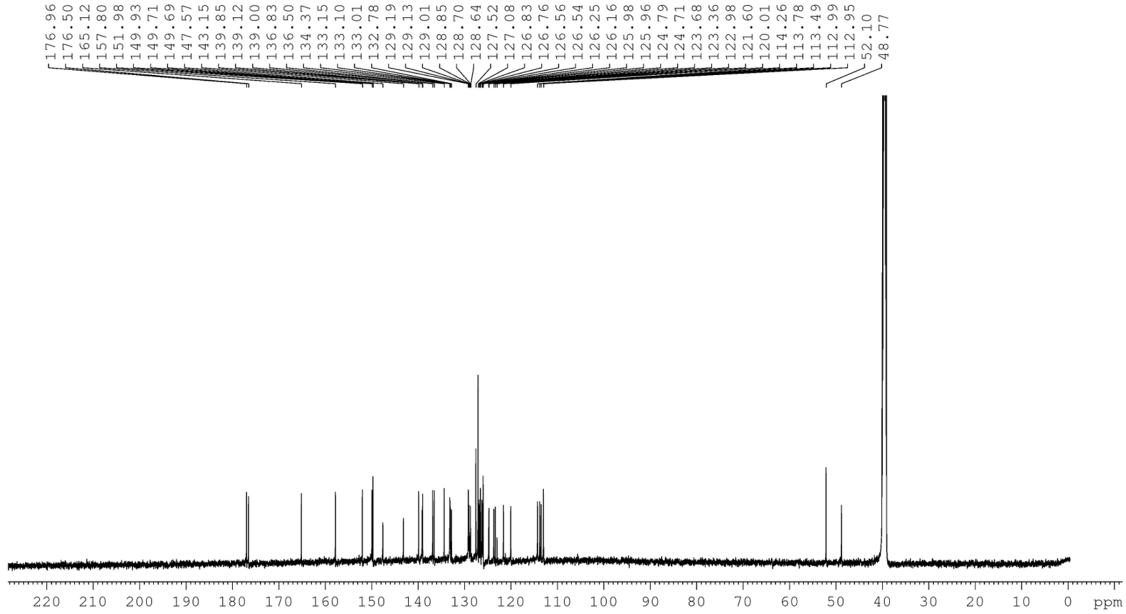

Figure S21. ^{13}C-NMR spectrum of complex **4** (DMSO-d_6, 151 MHz).

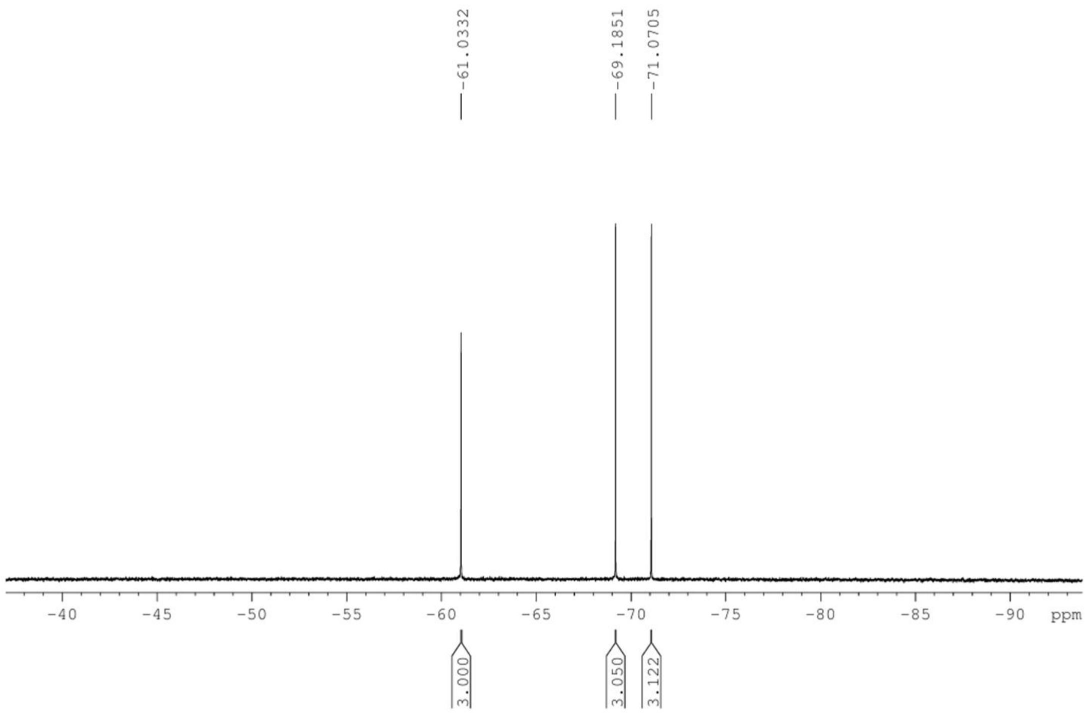

Figure S22. $^{19}F\{^{1}H\}$-NMR spectrum of complex **4** (DMSO-d_6, 377 MHz).

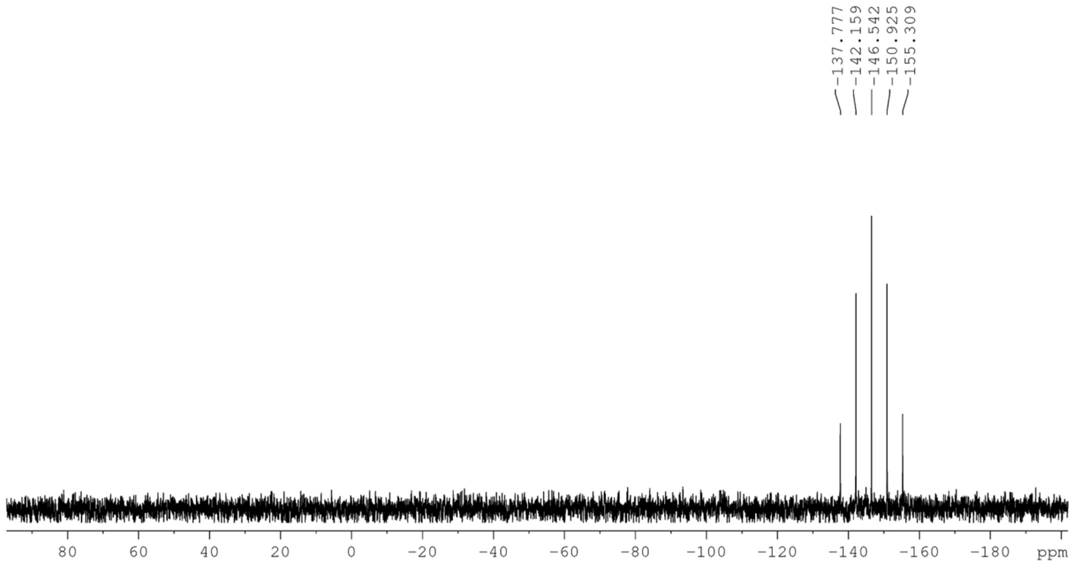

Figure S23. ^{31}P-NMR spectrum of complex **4** (DMSO-d_6, 162 MHz).

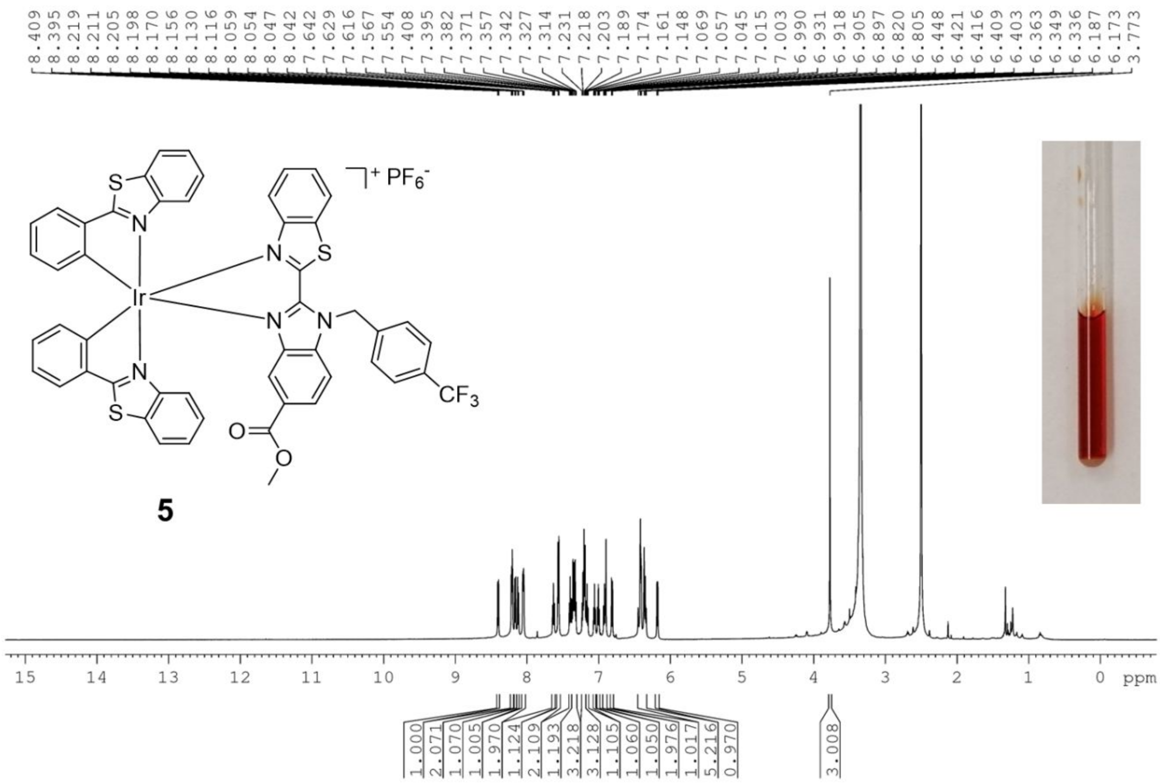

Figure S24. ^{1}H-NMR spectrum of complex **5** (DMSO-d$_6$, 600 MHz).

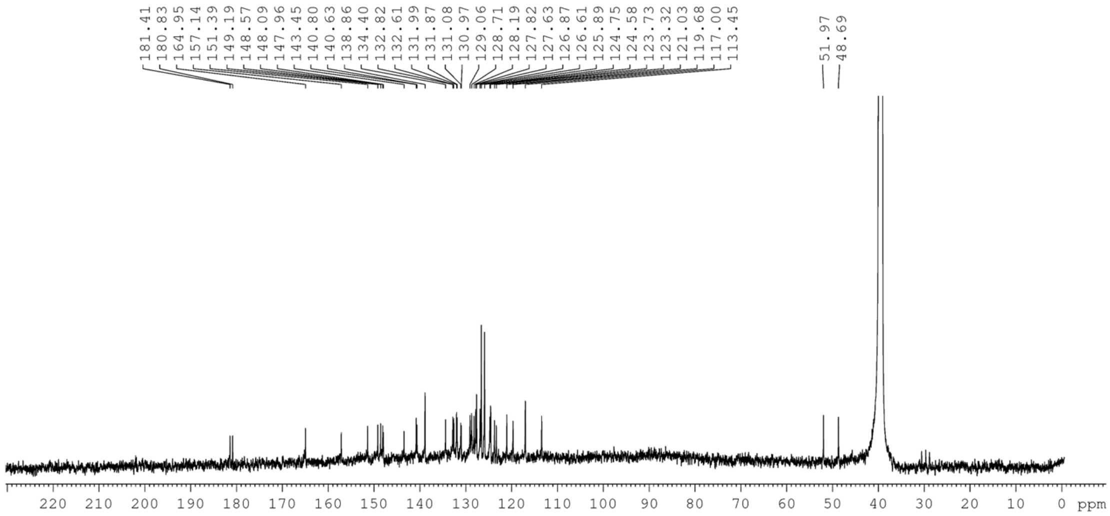

Figure S25. ^{13}C-NMR spectrum of complex **5** (DMSO-d$_6$, 151 MHz).

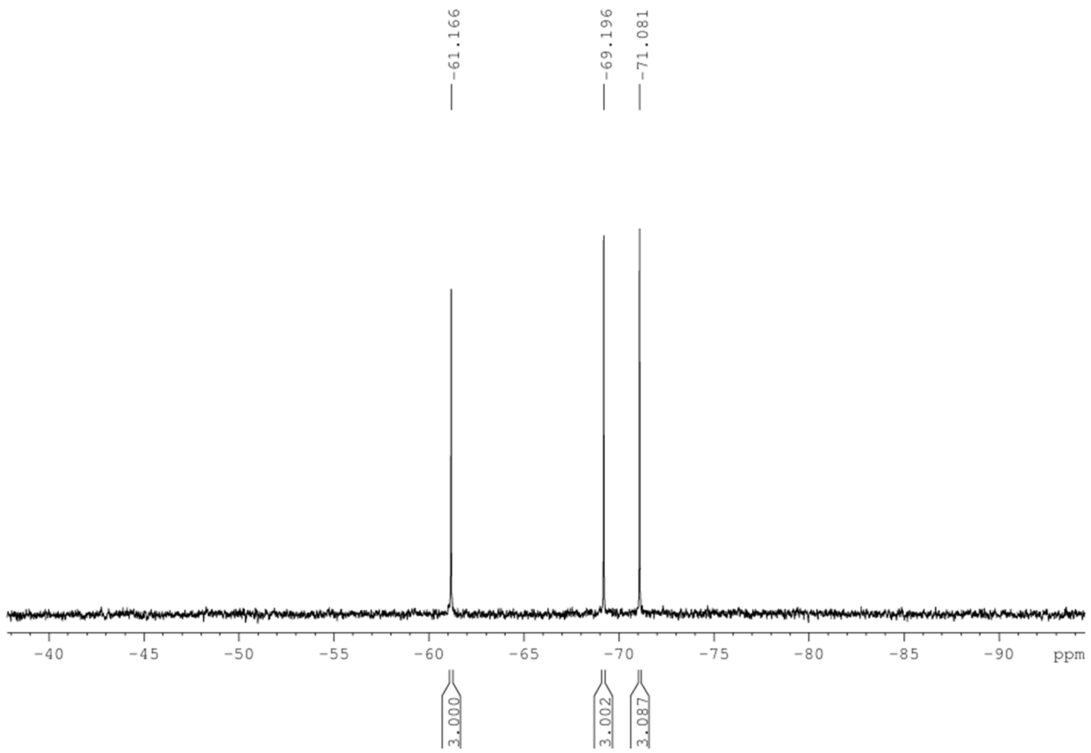

Figure S26. $^{19}F\{^{1}H\}$-NMR spectrum of complex **5** (DMSO-d_6, 377 MHz).

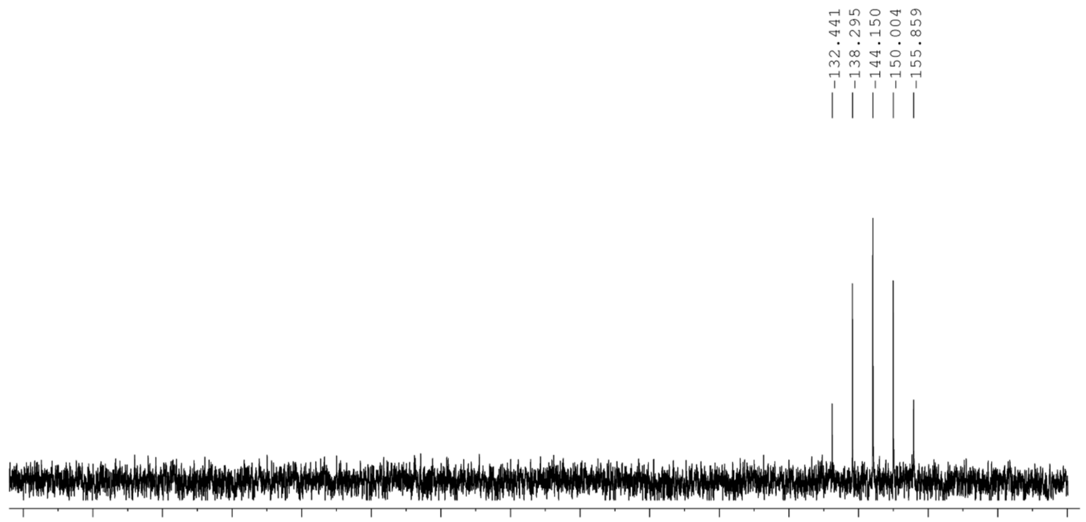

Figure S27. ^{31}P-NMR spectrum of complex **5** (DMSO-d_6, 121 MHz).

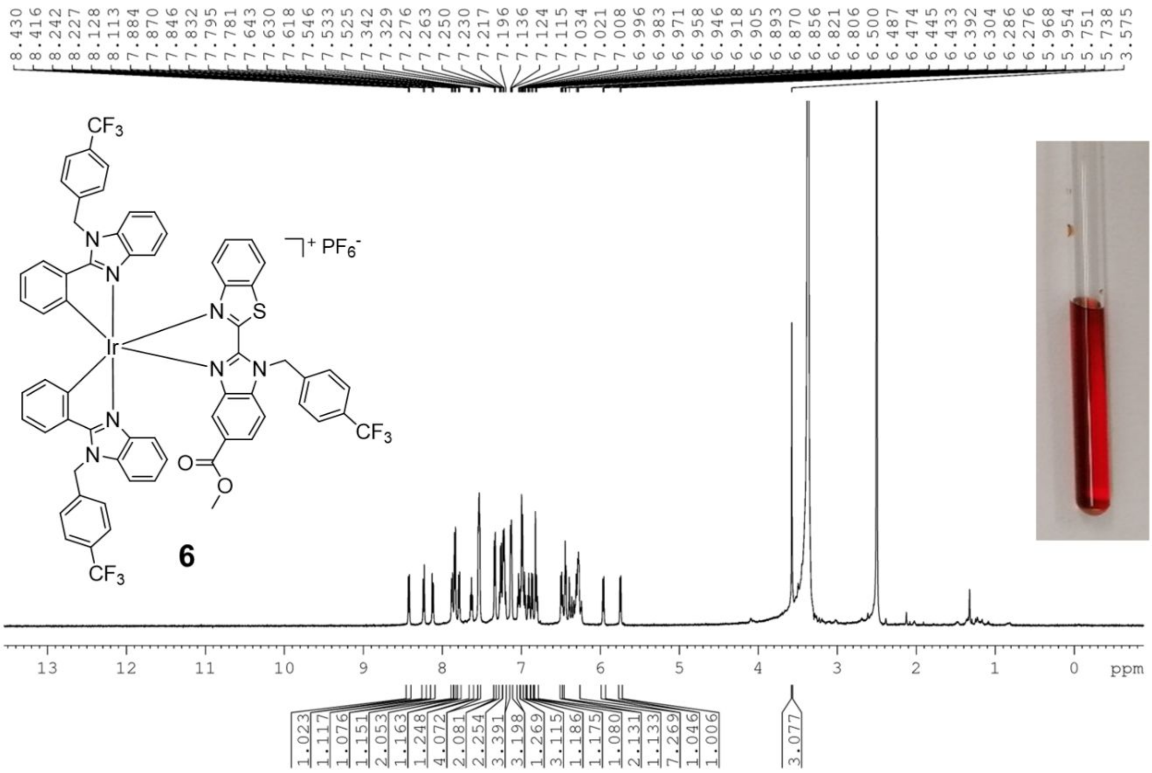

Figure S28. ^{1}H-NMR spectrum of complex **6** (DMSO-d_6, 600 MHz).

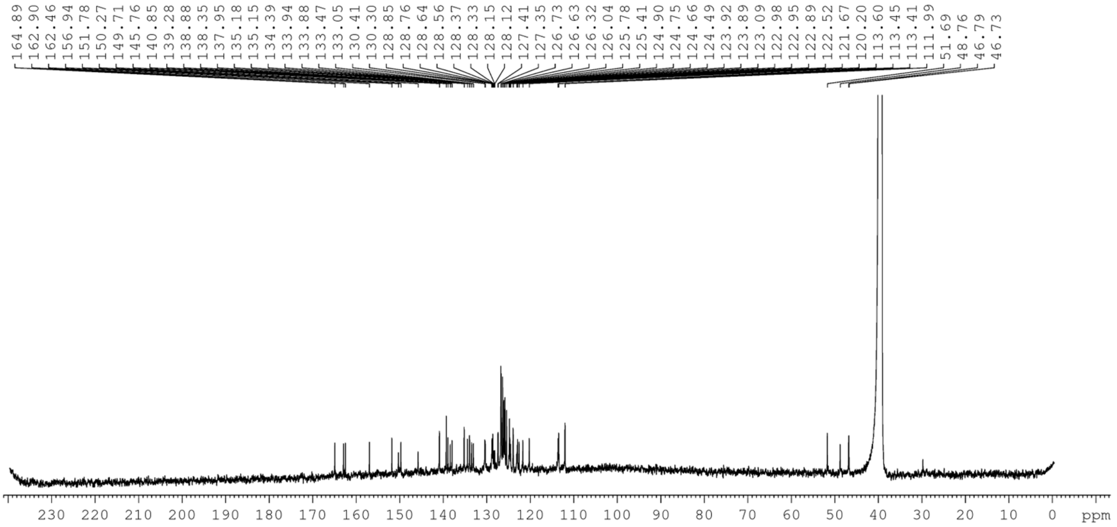

Figure S29. ^{13}C-NMR spectrum of complex **6** (DMSO-d_6, 151 MHz).

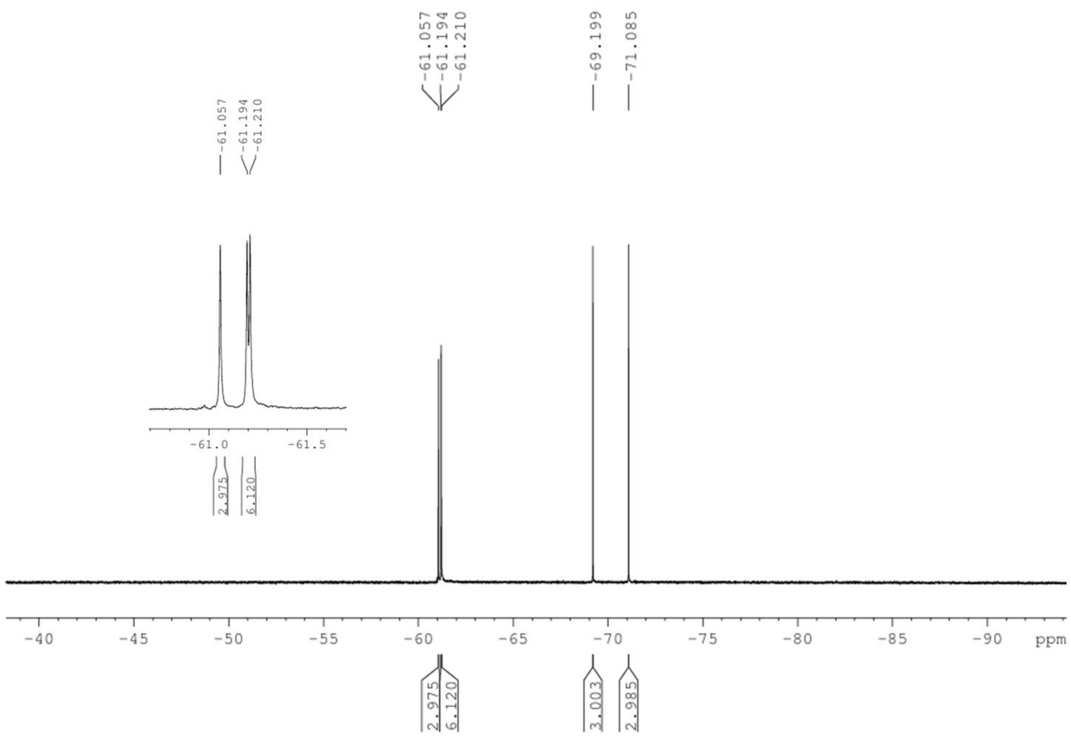

Figure S30. $^{19}F\{^{1}H\}$-NMR spectrum of complex **6** (DMSO-d_6, 377 MHz).

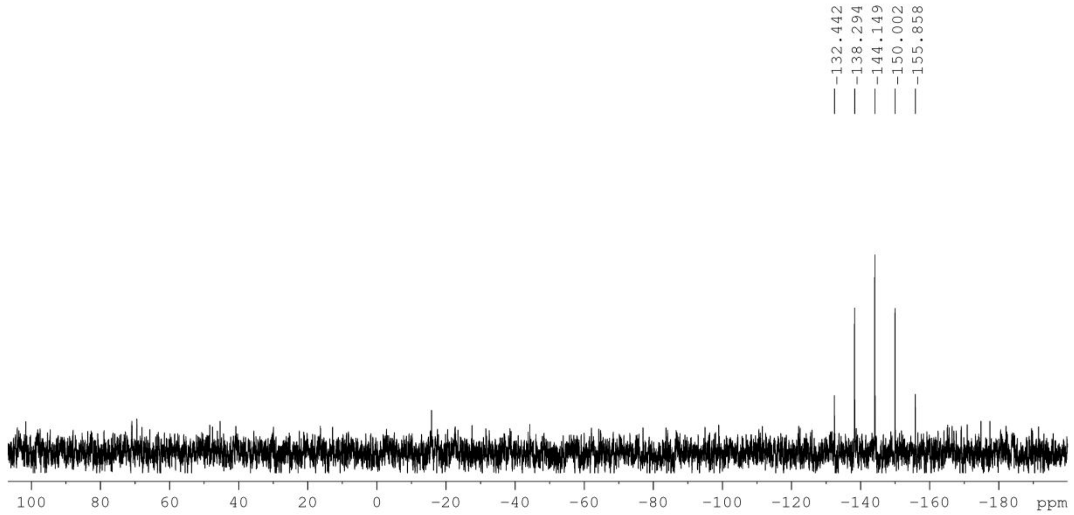

Figure S31. ^{31}P-NMR spectrum of complex **6** (DMSO-d_6, 121 MHz).

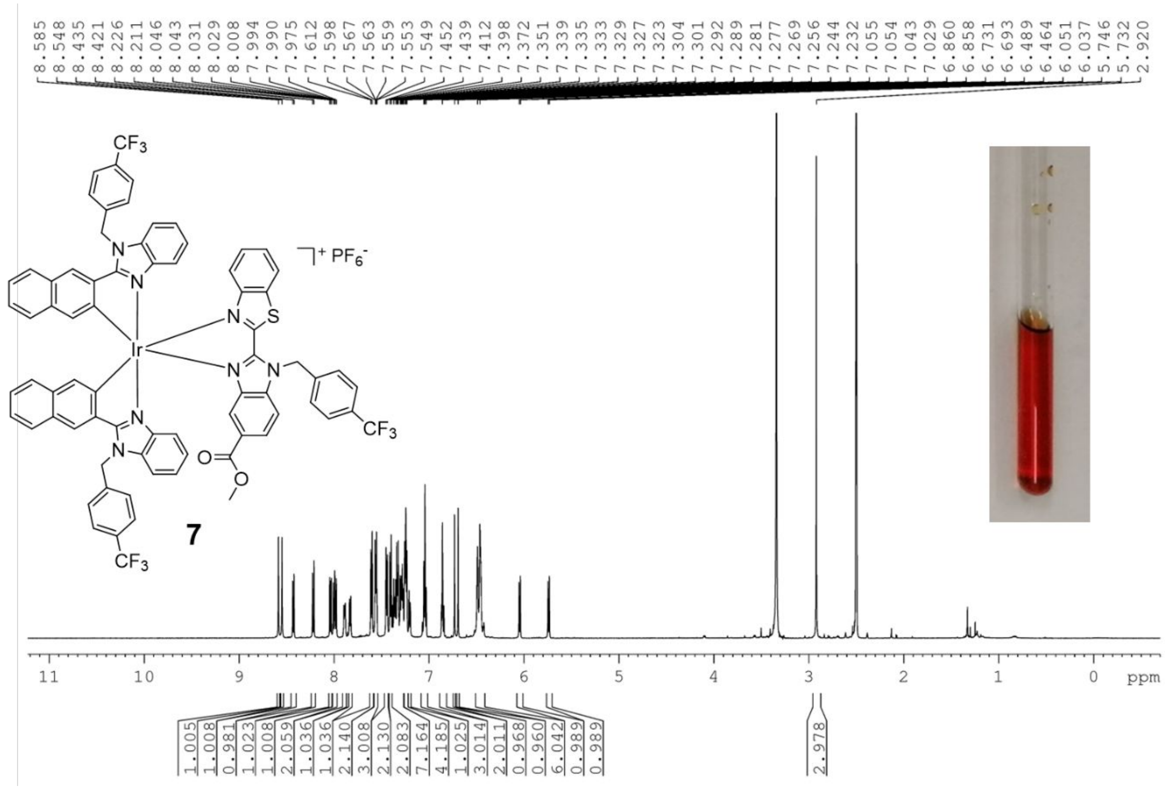

Figure S32. ^{1}H-NMR spectrum of complex **7** (DMSO-d_6, 600 MHz).

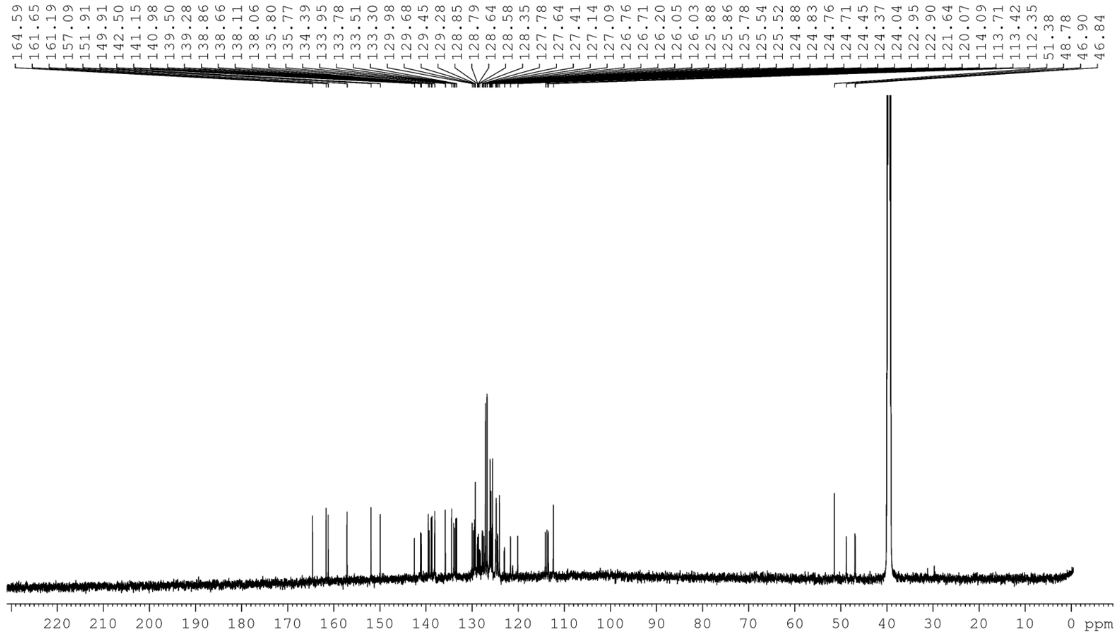

Figure S33. ^{13}C-NMR spectrum of complex **7** (DMSO-d_6, 151 MHz).

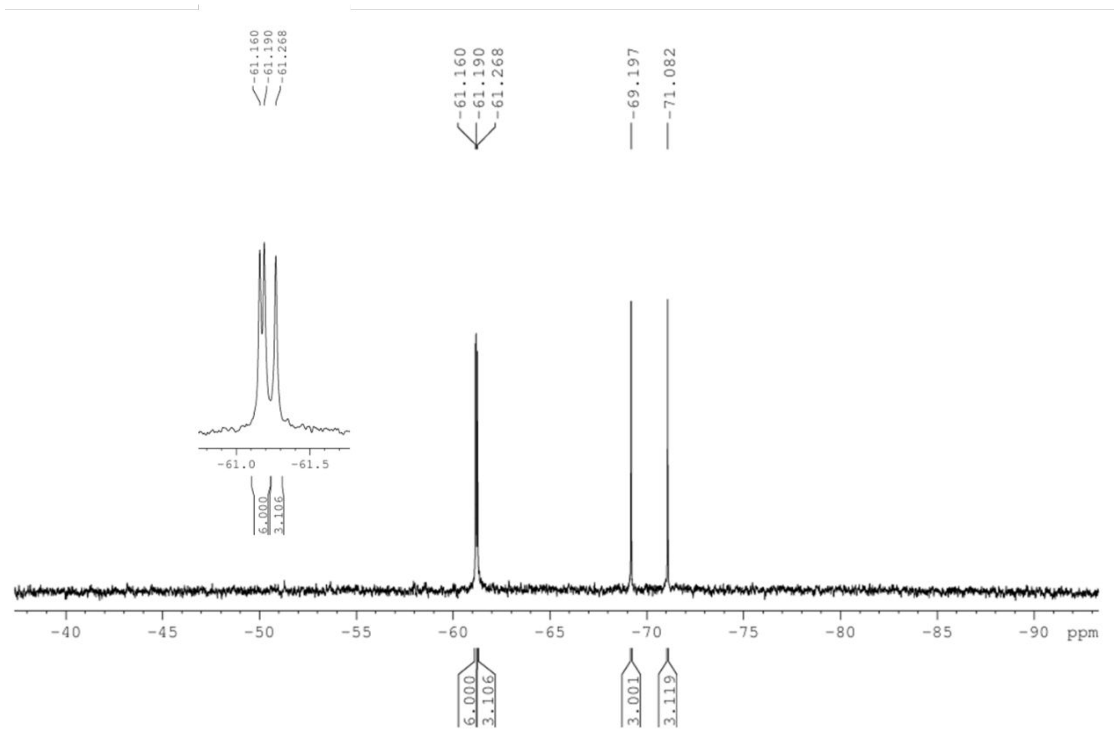

Figure S34. $^{19}F\{^1H\}$-NMR spectrum of complex **7** (DMSO-d_6, 377 MHz).

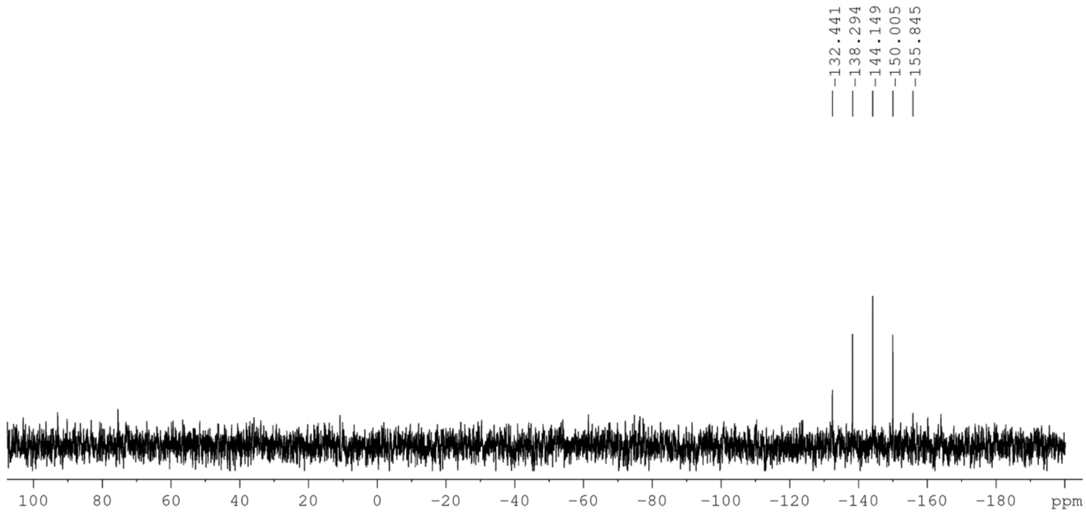

Figure S35. ^{31}P-NMR spectrum of complex **7** (DMSO-d_6, 121 MHz).

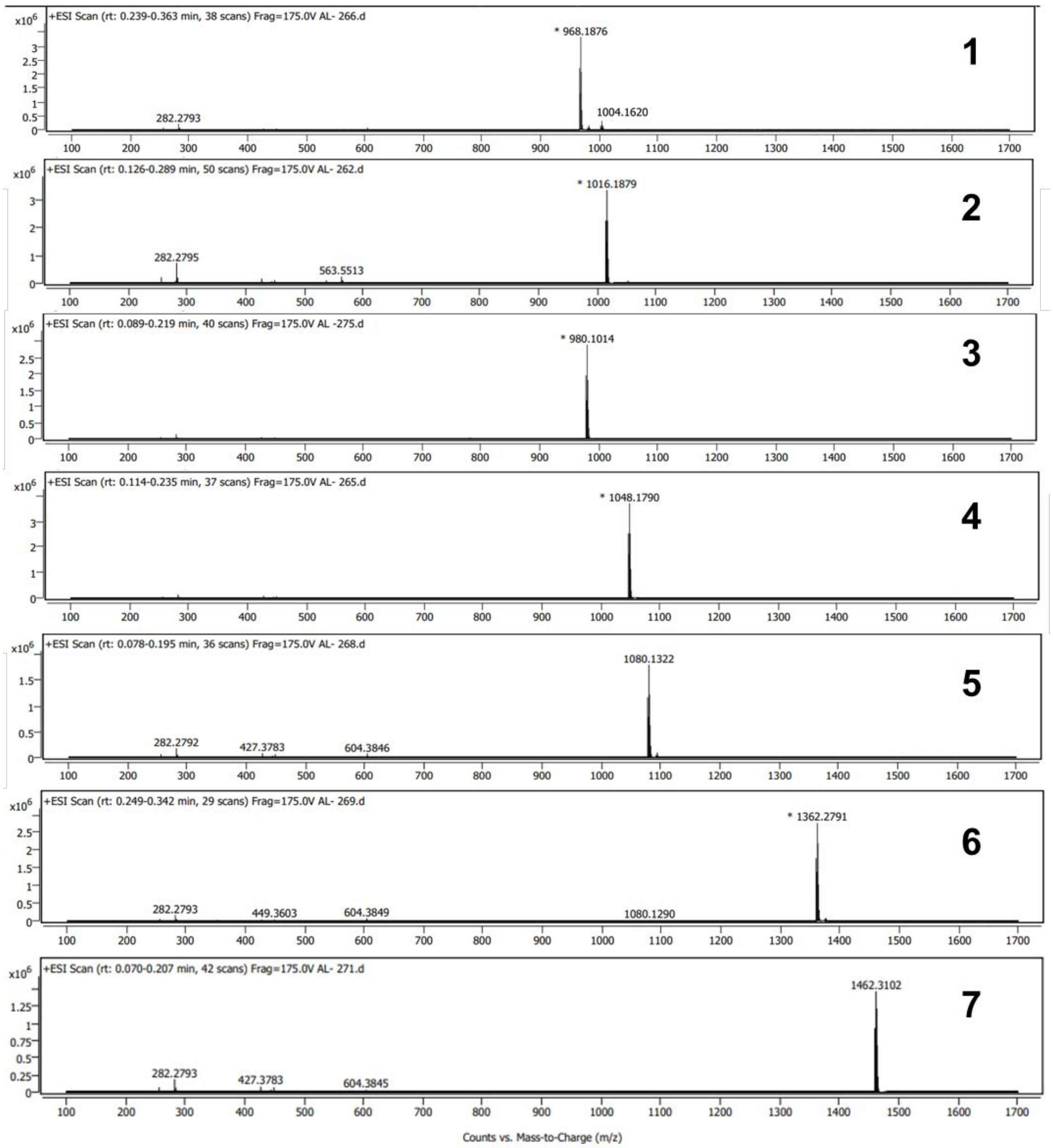

Figure S36. HR-ESI-MS spectra of complexes **1-7** (CH_3CN).

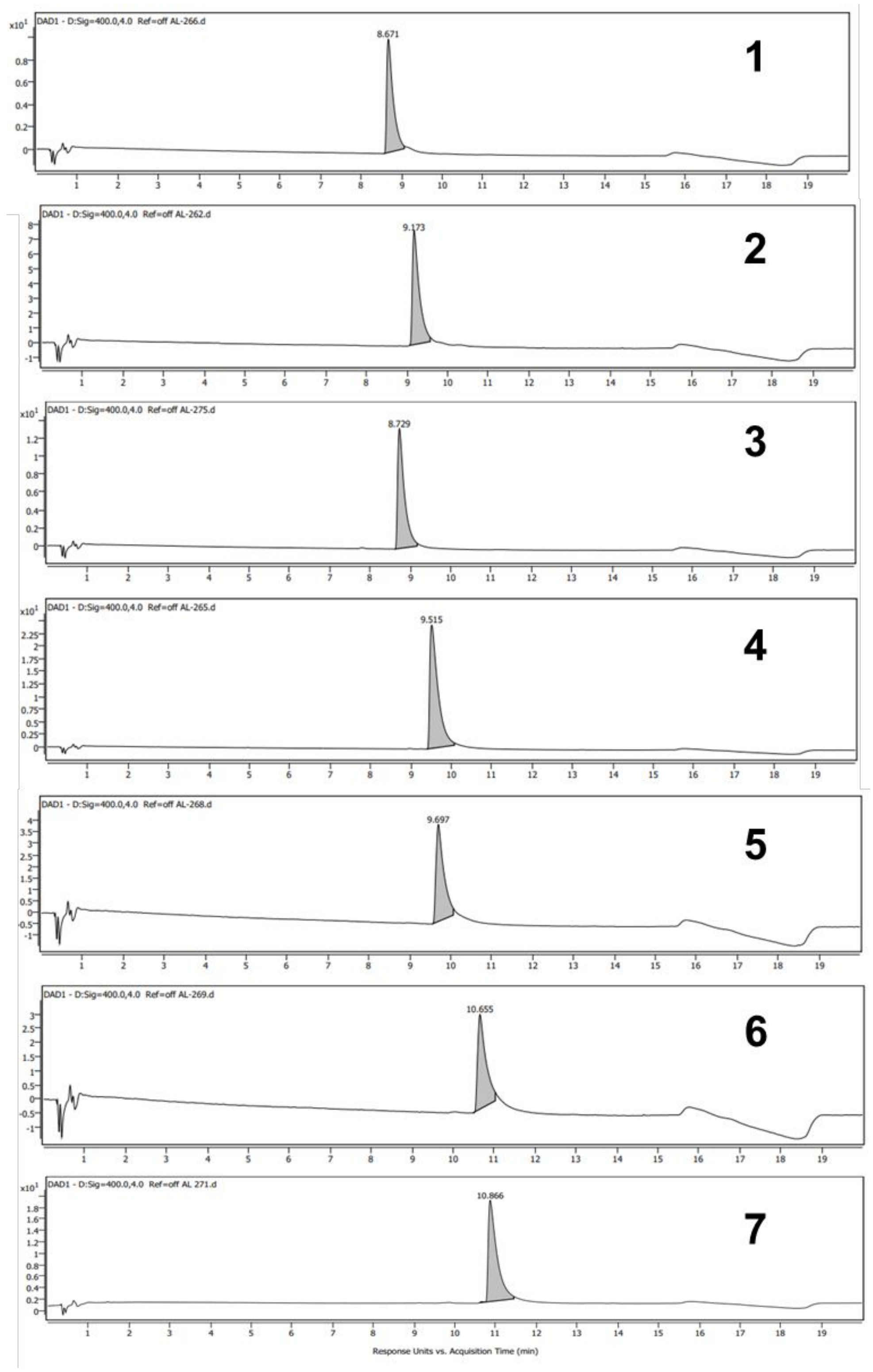

Figure S37. RP-HPLC chromatograms of complexes **1-7** (CH_3CN).

Table S1. Crystal data and structure refinement details for complex **2**.

Crystal data

$C_{50}H_{32}F_3IrN_5O_2S{\cdot}F_6P{\cdot}2(CH_2Cl_2)$	$Z = 2$
$M_r = 1330.89$	$F(000) = 1312$
Triclinic, $P\bar{1}$	$D_x = 1.787$ Mg m^{-3}
$a = 8.773$ (2) Å	Mo $K\alpha$ radiation, $\lambda = 0.71073$ Å
$b = 12.163$ (4) Å	Cell parameters from 9547 reflections
$c = 23.445$ (8) Å	$\theta = 2.4$–$27.4°$
$\alpha = 95.894$ (7)°	$\mu = 3.07$ mm^{-1}
$\beta = 94.461$ (10)°	$T = 100$ K
$\gamma = 93.811$ (7)°	Needle, orange
$V = 2473.8$ (14) Å^3	0.37 × 0.06 × 0.04 mm

Data collection

Bruker D8 Quest CCD diffractometer	8856 reflections with $I > 2\sigma(I)$
Radiation source: fine-focus sealed tube	$R_{int} = 0.054$
ω and ϕ scans	$\theta_{max} = 25.9°$, $\theta_{min} = 1.7°$
Absorption correction: multi-scan (*SADABS*; Krause, 2015)	$h = -10 \rightarrow 10$
$T_{min} = 0.599$, $T_{max} = 0.746$	$k = -14 \rightarrow 14$
133151 measured reflections	$l = -28 \rightarrow 28$
9557 independent reflections	

Refinement

Refinement on F^2	Primary atom site location: structure-invariant direct methods
Least-squares matrix: full	Secondary atom site location: difference Fourier map
$R[F^2 > 2\sigma(F^2)] = 0.0324$<br>$R[F^2$, all data$] = 0.0378$	Hydrogen site location: inferred from neighbouring sites
$wR[F^2 > 2\sigma(F^2)] = 0.0727$<br>$wR[F^2$, all data$] = 0.0761$	H-atom parameters constrained
$S = 1.123$	$w = 1/[\sigma^2(F_o^2) + (0.0131P)^2 + 14.2799P]$ where $P = (F_o^2 + 2F_c^2)/3$

9557 reflections	$(\Delta/\sigma)_{max}$ = 0.001
673 parameters	Δ□$_{max}$ = 2.03 e Å^{-3}
0 restraints	Δ□$_{min}$ = -1.52 e Å^{-3}

Table S2. Selected bond distances (Å) and bond angles (°) for complex **2** referring to the atom numbering in the image below:

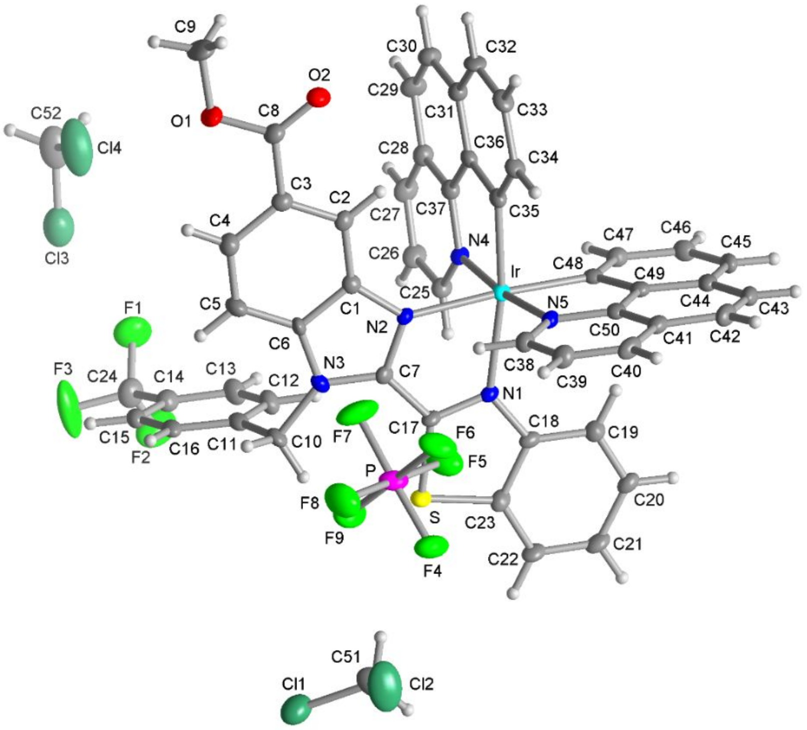

Ir—C35	2.022 (4)	P—F8	1.585 (4)
Ir—C48	2.023 (4)	P—F4	1.593 (3)
Ir—N5	2.053 (3)	P—F5	1.595 (3)
Ir—N4	2.053 (3)	P—F9	1.596 (3)
Ir—N2	2.145 (3)	P—F7	1.599 (3)
Ir—N1	2.185 (3)	P—F6	1.604 (3)
S—C17	1.719 (4)		
S—C23	1.731 (4)		
C35—Ir—C48	87.92 (16)	C17—S—C23	89.5 (2)
C35—Ir—N5	93.09 (15)		
C48—Ir—N5	81.42 (15)	F8—P—F4	90.0 (2)
C35—Ir—N4	81.72 (15)	F8—P—F5	179.5 (2)

C48—Ir—N4	93.99 (15)	F4—P—F5	89.56 (19)
N5—Ir—N4	173.22 (14)	F8—P—F9	90.7 (2)
C35—Ir—N2	96.07 (14)	F4—P—F9	90.77 (17)
C48—Ir—N2	176.01 (14)	F5—P—F9	88.95 (19)
N5—Ir—N2	98.34 (13)	F8—P—F7	91.4 (2)
N4—Ir—N2	86.59 (13)	F4—P—F7	178.3 (2)
C35—Ir—N1	171.24 (14)	F5—P—F7	89.0 (2)
C48—Ir—N1	100.39 (14)	F9—P—F7	90.20 (19)
N5—Ir—N1	90.91 (13)	F8—P—F6	90.46 (19)
N4—Ir—N1	94.83 (13)	F4—P—F6	90.20 (18)
N2—Ir—N1	75.63 (13)	F5—P—F6	89.89 (18)
		F9—P—F6	178.5 (2)
		F7—P—F6	88.80 (19)

The PLATON[1,2]-listing "Analysis of Short Ring-Interactions" for possible π-stacking interactions yielded a single π-stacking interaction for complex **2** with rather short centroid-centroid contacts (<3.8 Å), near parallel ring planes (alpha < 10° to ~0° or even exactly 0° by symmetry), small slip angles (β, γ <25°) and vertical displacements (slippage <1.5 Å) which translate into a sizable overlap of the aryl-plane areas (**Scheme S1**, **Table S3**, **Figure S38**).[3,4]

Scheme S1. Graphical presentation of the parameters used for the description of π-π stacking.

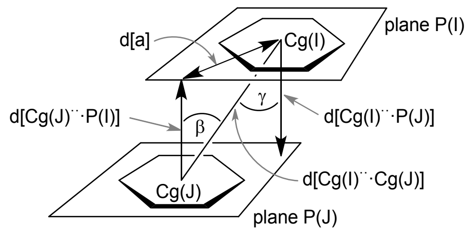

Significant intermolecular C-H···π contacts start below around 2.7 Å for the (C-H)···ring centroid distances with H-perp also starting at below 2.6-2.7 Å and C-H··Cg > 145° (**Scheme S2, Table S5**, **Figure S38**).[5–10]

Scheme S2. Graphical presentation of the parameters used for the description of CH-π interactions.[6]

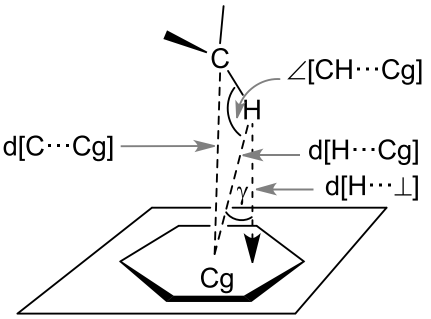

Table S3. Packing Analysis for complex **2** for possible π···π interactions (see **Scheme S1** for explanation).

==

Analysis of Short Ring-Interactions with Cg-Cg Distances < 4.0 Ang., Alpha < 20.000 Deg. and Beta < 60.0 Deg.

==

- Cg(I) = Plane number I (= ring number in () above)
- Alpha = Dihedral Angle between Planes I and J (Deg)
- Beta = Angle Cg(I)-->Cg(J) or Cg(I)-->Me vector and normal to plane I (Deg)
- Gamma = Angle Cg(I)-->Cg(J) vector and normal to plane J (Deg)
- Cg-Cg = Distance between ring Centroids (Ang.)
- CgI_Perp = Perpendicular distance of Cg(I) on ring J (Ang.)
- CgJ_Perp = Perpendicular distance of Cg(J) on ring I (Ang.)
- Slippage = Distance between Cg(I) and Perpendicular Projection of Cg(J) on Ring I (Ang).

Cg(I) Res(I) Cg(J) [ARU(J)]	Cg-Cg	Alpha	Beta	Gamma	CgI_Perp	CgJ_Perp	Slippage
Cg(9) [1] -> Cg(14) [1565.01]	3.604(3)	4.5(2)	26.5	25.3	-3.2578(18)	3.2261(17)	1.606
Cg(14) [1] -> Cg(9) [1545.01]	3.604(3)	4.5(2)	25.3	26.5	3.2262(17)	-3.2578(18)	1.541

[1545] = X,-1+Y,Z

[1565] = X,1+Y,Z

Cg9 = centroid of ring C11-C12-C13-C14-C15-C16

Cg14 = centroid of ring C44-C45-C46-C47-C48-C29

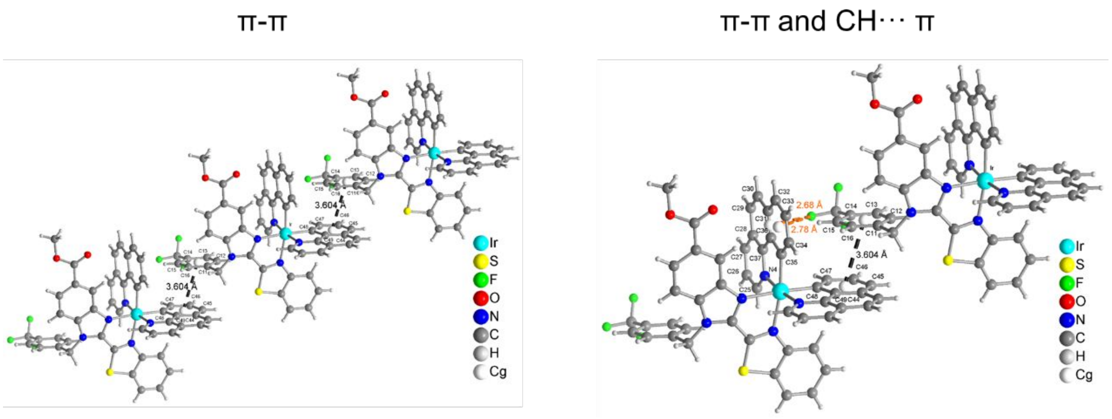

Figure S38. π-π and part of the C-H···π interactions in the packing of complex **2** in the crystal, indicated as dashed black lines for π-π and dashed orange lines for C-H···π with the centroid-centroid contact given in Å. Further details of these π-π and C-H···π interactions, including the symmetry transformations are listed in the above **Tables S3** and **S4** (Cg = ring centroid). H atoms are not shown for clarity.

Table S4. Analysis of *inter*molecular C-H···Cg(Pi-Ring) Interactions (H···Cg < 3.0 Ang. - Gamma < 30.0 Deg) in complex **2** (see **Scheme S2** for explanation).

- Cg(J) = Center of gravity of ring J (Plane number above)
- H-Perp = Perpendicular distance of H to ring plane J
- Gamma = Angle between Cg-H vector and ring J normal
- C-H..Cg = C-H-Cg angle (degrees)
- C..Cg = Distance of X to Cg (Angstrom)
- C-H, Pi = Angle of the X-H bond with the Pi-plane (i.e.' Perpendicular = 90 degrees, Parallel = 0 degrees)

X--H(I) Res(I) Cg(J) [ARU(J)]	H..Cg	H-Perp	Gamma	X-H..Cg	X..Cg	X-H,Pi
C(15) -H(15) [1] -> Cg(12) [1565.01]	2.78	2.64	17.96	141	3.563(5)	68
C(15) -H(15) [1] -> Cg(19) [1565.01]	2.68	2.64	9.37	166	3.606(5)	68
C(15) -H(15) [1] -> Cg(21) [1565.01]	2.95	2.65	26.16	166	3.882(5)	68
C(52) -H(52B) [4] -> Cg(17) [2566.01]	2.85	2.82	8.42	147	3.723(7)	49 (from CH_2Cl_2)

[1565] = X,1+Y,Z

[2566] = -X,1-Y,1-Z

Cg12 = centroid of ring C31-C32-C33-C34-C35-C36

Cg19 = centroid of ring C28-C29-C30-C31-C32-C33-C34-C35-C36-C37

Cg21 = centroid of ring N4-C25-C26-C27-C28-C29-C30-C31-C32-C33-C34-C35-C36-C37

Cg17 = centroid of ring N4-C25-C26-C27-C28-C29-C30-C31-C36-C37

Table S5. Analysis of Potential Hydrogen Bonds and Schemes with d(D···A) < R(D)+R(A)+0.50, d(H···A) < R(H)+R(A)-0.12 Ang., D-H···A > 100.0 Deg

Donor --- H....Acceptor [ARU]	D - H	H...A	D...A	D - H...A
C(21) --H(21) ..F(9) [2667.02]	0.95	2.52	3.425(5)	160
C(25) --H(25) ..F(6) [1455.02]	0.95	2.49	3.364(6)	153
C(38) --H(38) ..F(5) [1555.02]	0.95	2.46	2.978(6)	114
C(51) --H(51B) ..F(4) [1555.02]	0.99	2.29	3.238(7)	159 (from CH_2Cl_2)

[1455.] = [1_455] = -1+x,y,z

[2667.] = [2_667] = 1-x,1-y,2-z

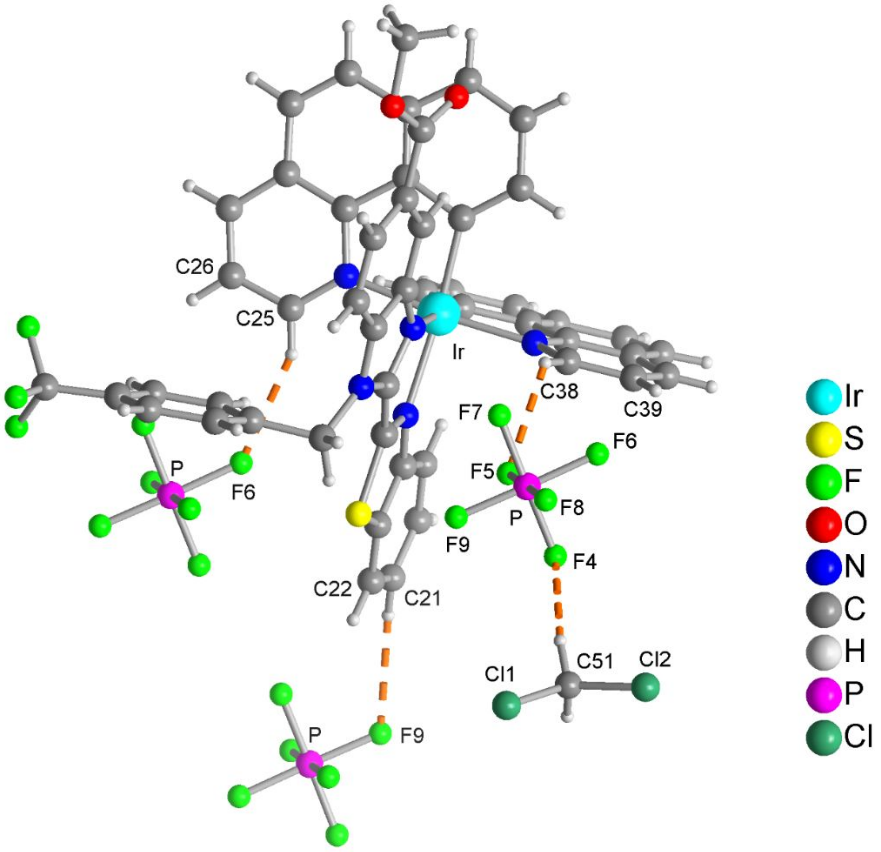

Figure S39. C-H···F contacts in the packing of complex **2** in the crystal, indicated as dashed orange lines. Further details of these C-H···F interactions, including the symmetry transformations are listed in the above **Table S5**.

Table S6. Crystal data and structure refinement details for complex **5**.

Crystal data

$C_{50}H_{32}F_3IrN_5O_2S_3 \cdot F_6P \cdot 3(CH_2Cl_2)$	$Z = 2$
$M_r = 1479.93$	$F(000) = 1460$
Triclinic, $P\bar{1}$	$D_x = 1.762$ Mg m^{-3}
$a = 11.2743$ (8) Å	Mo $K\alpha$ radiation, $\lambda = 0.71073$ Å
$b = 15.6813$ (12) Å	Cell parameters from 9928 reflections
$c = 16.4064$ (12) Å	$\theta = 2.3$–$30.7°$
$\alpha = 86.346$ (3)°	$\mu = 2.90$ $\mu\mu^{-1}$
$\beta = 87.239$ (2)°	$T = 100$ K
$\gamma = 74.660$ (2)°	Prism, orange
$V = 2790.1$ (4) Å^3	0.24 × 0.09 × 0.09 mm

Data collection

Bruker D8 Quest CCD diffractometer	11476 reflections with $I > 2\sigma(I)$
Radiation source: fine-focus sealed tube	$R_{int} = 0.033$
ω and ϕ scans	$\theta_{max} = 26.9°$, $\theta_{min} = 1.8°$
Absorption correction: multi-scan (*SADABS*; Krause, 2015)	$h = -14 \rightarrow 14$
$T_{min} = 0.623$, $T_{max} = 0.746$	$k = -19 \rightarrow 19$
193981 measured reflections	$l = -20 \rightarrow 20$
11994 independent reflections	

Refinement

Refinement on F^2	Primary atom site location: structure-invariant direct methods
Least-squares matrix: full	Secondary atom site location: difference Fourier map
$R[F^2 > 2\sigma(F^2)] = 0.0240$<br>$R[F^2$, all data$] = 0.0267$	Hydrogen site location: inferred from neighbouring sites
$wR[F^2 > 2\sigma(F^2)] = 0.0557$<br>$wR[F^2$, all data$] = 0.0582$	H-atom parameters constrained
$S = 1.165$	$w = 1/[\sigma^2(F_o^2) + (0.0155P)^2 + 7.6433P]$

	where $P = (F_o^2 + 2F_c^2)/3$
11994 reflections	$(\Delta/\sigma)_{max}$ = 0.005
722 parameters	Δ□$_{max}$ = 1.98 e Å^{-3}
0 restraints	Δ□$_{min}$ = -1.28 e Å^{-3}

Table S7. Selected bond distances (Å) and bond angles (°) for complex **5** referring to the atom numbering in the image below:

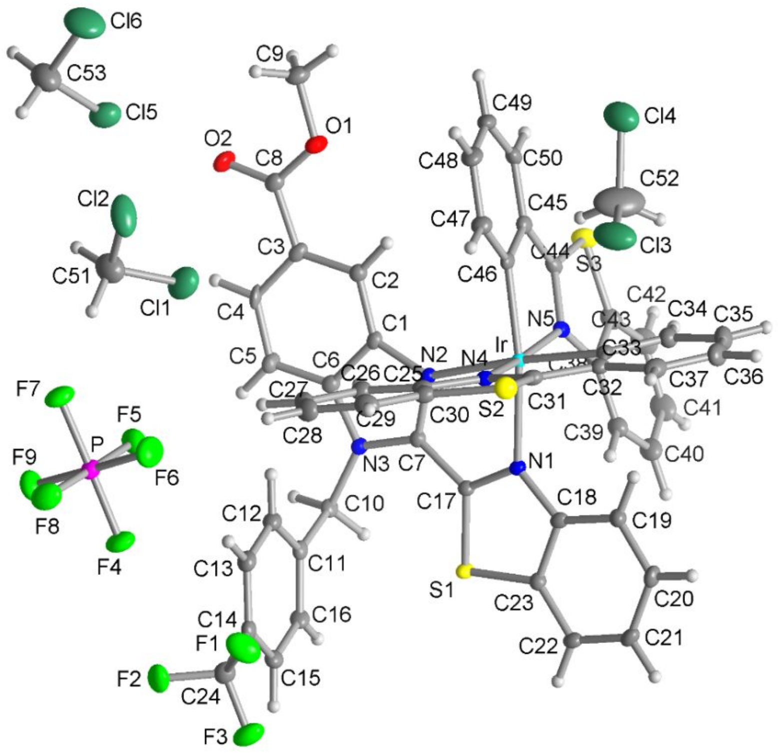

Ir—C46	2.013 (3)	S1—C17	1.714 (3)
Ir—C33	2.015 (3)	S1—C23	1.725 (3)
Ir—N5	2.056 (2)	S2—C31	1.718 (3)
Ir—N4	2.064 (2)	S2—C30	1.736 (3)
Ir—N2	2.162 (2)	S3—C44	1.724 (3)
Ir—N1	2.201 (2)	S3—C43	1.739 (3)
F1—C24	1.345 (3)	P—F4	1.5900 (19)
F2—C24	1.338 (3)	P—F6	1.595 (2)
F3—C24	1.332 (3)	P—F8	1.6001 (19)
		P—F9	1.6006 (19)
		P—F5	1.6082 (19)
		P—F7	1.6087 (18)

C46—Ir—C33	88.62 (10)	C17—S1—C23	89.13 (12)
C46—Ir—N5	80.27 (10)	C31—S2—C30	89.54 (13)
C33—Ir—N5	93.32 (10)	C44—S3—C43	89.78 (13)
C46—Ir—N4	92.89 (10)		
C33—Ir—N4	80.03 (10)		
N5—Ir—N4	170.63 (8)		
C46—Ir—N2	95.74 (9)		
C33—Ir—N2	175.63 (9)		
N5—Ir—N2	87.25 (8)		
N4—Ir—N2	99.89 (8)		
C46—Ir—N1	171.01 (9)		
C33—Ir—N1	100.34 (9)		
N5—Ir—N1	98.27 (8)		
N4—Ir—N1	89.48 (8)		
N2—Ir—N1	75.30 (8)		

The PLATON[1,2]-listing "Analysis of Short Ring-Interactions" for possible π-stacking interactions yielded significant π-stacking for complex **5** with rather short centroid-centroid contacts (<3.8 Å), near parallel ring planes (alpha < 10° to ~0° or even exactly 0° by symmetry), small slip angles (β, γ <25°) and vertical displacements (slippage <1.5 Å) which translate into a sizable overlap of the aryl-plane areas (**Scheme S3**, **Table S8**, **Figure S40**).[3,4]

Scheme S3. Graphical presentation of the parameters used for the description of π-π stacking.

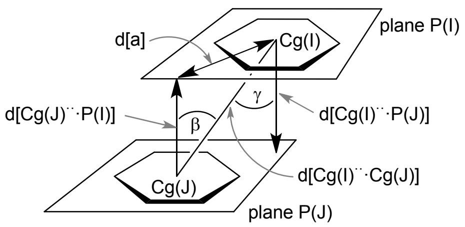

Significant intermolecular C-H···π contacts start below around 2.7 Å for the (C-)H···ring centroid distances with H-perp also starting at below 2.6-2.7 Å and C-H··Cg > 145° (**Scheme S4, Table S9**, **Figure S40**).[6–11]

Scheme S4. Graphical presentation of the parameters used for the description of CH-π interactions.[6]

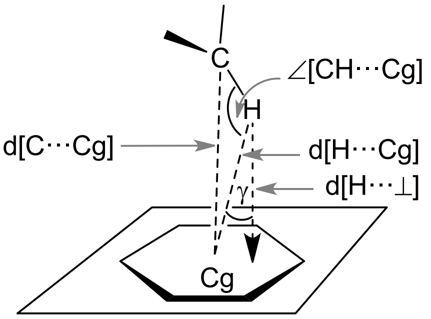

Table S8. Packing Analysis for complex **5** for possible π···π interactions (see **Scheme S4** for explanation).

==

Analysis of Short Ring-Interactions with Cg-Cg Distances < 4.0 Ang., Alpha < 20.000 Deg. and Beta < 60.0 Deg.

==

- Cg(I) = Plane number I (= ring number in () above)
- Alpha = Dihedral Angle between Planes I and J (Deg)
- Beta = Angle Cg(I)-->Cg(J) or Cg(I)-->Me vector and normal to plane I (Deg)
- Gamma = Angle Cg(I)-->Cg(J) vector and normal to plane J (Deg)
- Cg-Cg = Distance between ring Centroids (Ang.)
- CgI_Perp = Perpendicular distance of Cg(I) on ring J (Ang.)
- CgJ_Perp = Perpendicular distance of Cg(J) on ring I (Ang.)
- Slippage = Distance between Cg(I) and Perpendicular Projection of Cg(J) on Ring I (Ang).

Cg(I)	Res(I)	Cg(J)	[ARU(J)]	Cg-Cg	Alpha	Beta	Gamma	CgI_Perp	CgJ_Perp	Slippage
Cg(4)	[1] ->	Cg(10)	[2566.01]	3.8029(15)	1.00(12)	25.9	26.9	3.3899(10)	3.4195(10)	1.664
Cg(10)	[1] ->	Cg(4)	[2566.01]	3.8028(15)	1.00(12)	26.9	25.9	3.4195(10)	3.3899(10)	1.723
Cg(10)	[1] ->	Cg(10)	[2566.01]	3.5446(15)	0.00(12)	16.1	16.1	3.4050(10)	3.4050(10)	0.985
Cg(10)	[1] ->	Cg(15)	[2566.01]	3.4909(13)	0.42(10)	13.3	13.0	3.4012(10)	3.3970(8)	0.804
Cg(15)	[1] ->	Cg(10)	[2566.01]	3.4909(13)	0.42(10)	13.0	13.3	3.3969(8)	3.4012(10)	0.786
Cg(15)	[1] ->	Cg(15)	[2566.01]	3.6961(12)	0.00(7)	23.1	23.1	3.3997(8)	3.3997(8)	1.450

[2566] = -X,1-Y,1-Z

Cg4 = centroid of ring S1-N1-C17-C18-C23

Cg10 = centroid of ring C18-C19-C20-C21-C22-C23

C15 = centroid of ring S1-N1-C17-C18- C19-C20-C21-C22-C23

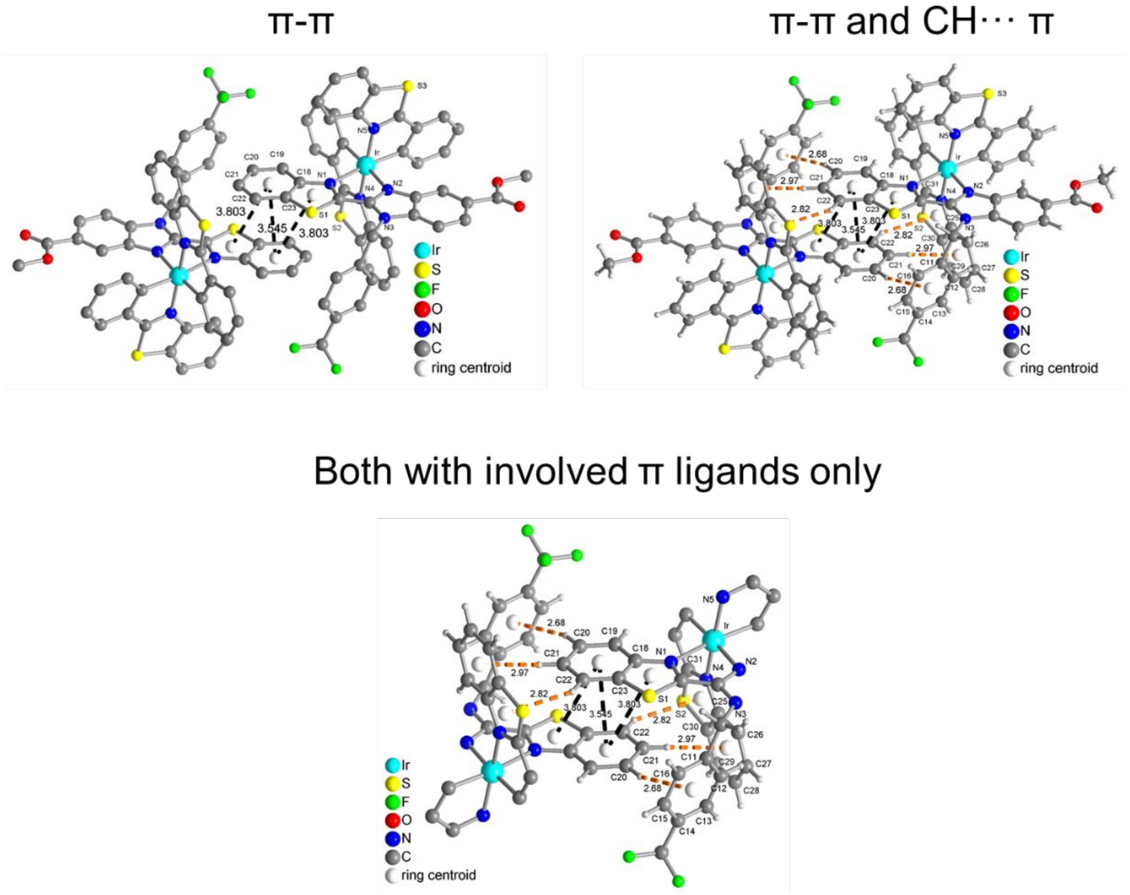

Figure S40. π-π and part of the C-H$\cdots\pi$ interactions in the packing of complex **5** in the crystal, indicated as dashed black lines for π-π and dashed orange lines for C-H$\cdots\pi$ with the centroid-centroid contact given in Å. Further details of these π-π and C-H$\cdots\pi$ interactions, including the symmetry transformations are listed in the above **Tables S8** and **S9** (Cg = ring centroid). H atoms are not shown for clarity.

Table S9. Analysis of *inter*molecular C-H...Cg(Pi-Ring) Interactions (H..Cg < 3.0 Ang. - Gamma < 30.0 Deg) in complex **5** (see **Scheme S4** for explanation).

- Cg(J) = Center of gravity of ring J (Plane number above)
- H-Perp = Perpendicular distance of H to ring plane J
- Gamma = Angle between Cg-H vector and ring J normal
- C-H..Cg = C-H-Cg angle (degrees)
- C..Cg = Distance of X to Cg (Angstrom)
- C-H, Pi = Angle of the X-H bond with the Pi-plane (i.e.' Perpendicular = 90 degrees, Parallel = 0 degrees)

X--H(I) Res(I) Cg(J) [ARU(J)]	H..Cg	H-Perp	Gamma	X-H..Cg	X..Cg	X-H,Pi
C(4) -H(4) [1] -> Cg(9) [2656.01]	2.96	2.62	27.88	138	3.719(3)	71
C(20) -H(20) [1] -> Cg(9) [2566.01]	2.68	2.51	20.29	162	3.591(3)	82
C(21) -H(21) [1] -> Cg(11) [2566.01]	2.97	2.78	20.88	144	3.781(3)	60
C(21) -H(21) [1] -> Cg(16) [2566.01]	2.93	2.76	19.22	133	3.642(3)	60
C(22) -H(22) [1] -> Cg(5) [2566.01]	2.82	2.76	11.25	138	3.584(3)	59
C(40) -H(40) [1] -> Cg(11) [1455.01]	2.81	2.72	14.36	175	3.756(3)	78

[2656] = 1-X,-Y,1-Z

[2566] = -X,1-Y,1-Z

[1455] = -1+X,Y,Z

Cg5 = centroid of ring S2-N4-C25-C30-C31

Cg9 = centroid of ring C11-C12-C13-C14-C15-C16

Cg11 = centroid of ring C25-C26-C27-C28-C29-C30

Cg16 = centroid of ring S2-N4-C25-C26-C27-C28-C29-C30-C31

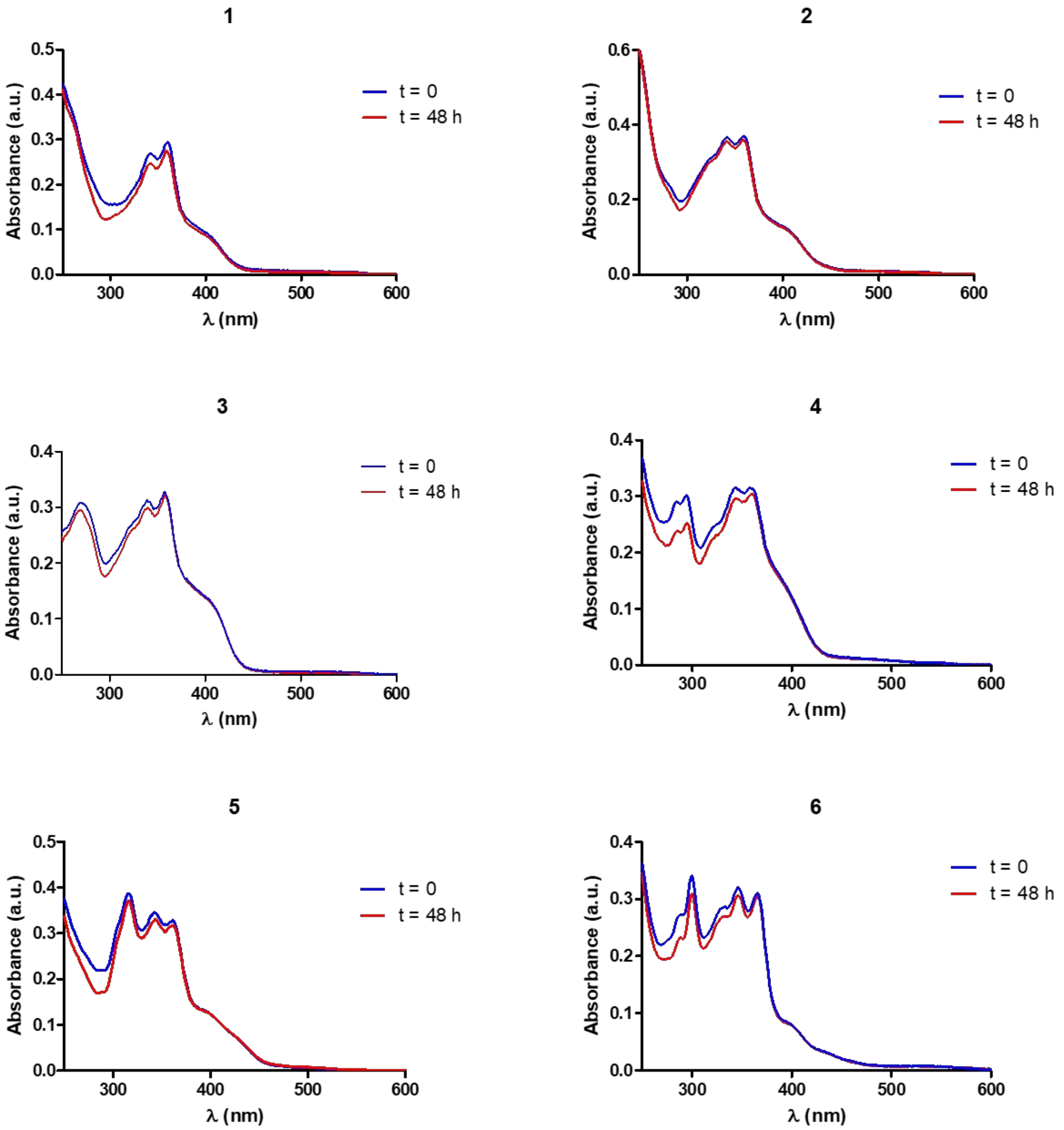

Figure S41. Monitoring of the UV/VIS absorption spectra of complexes **1-6** (10 μM) in DMSO after incubation at room temperature for 48 h.

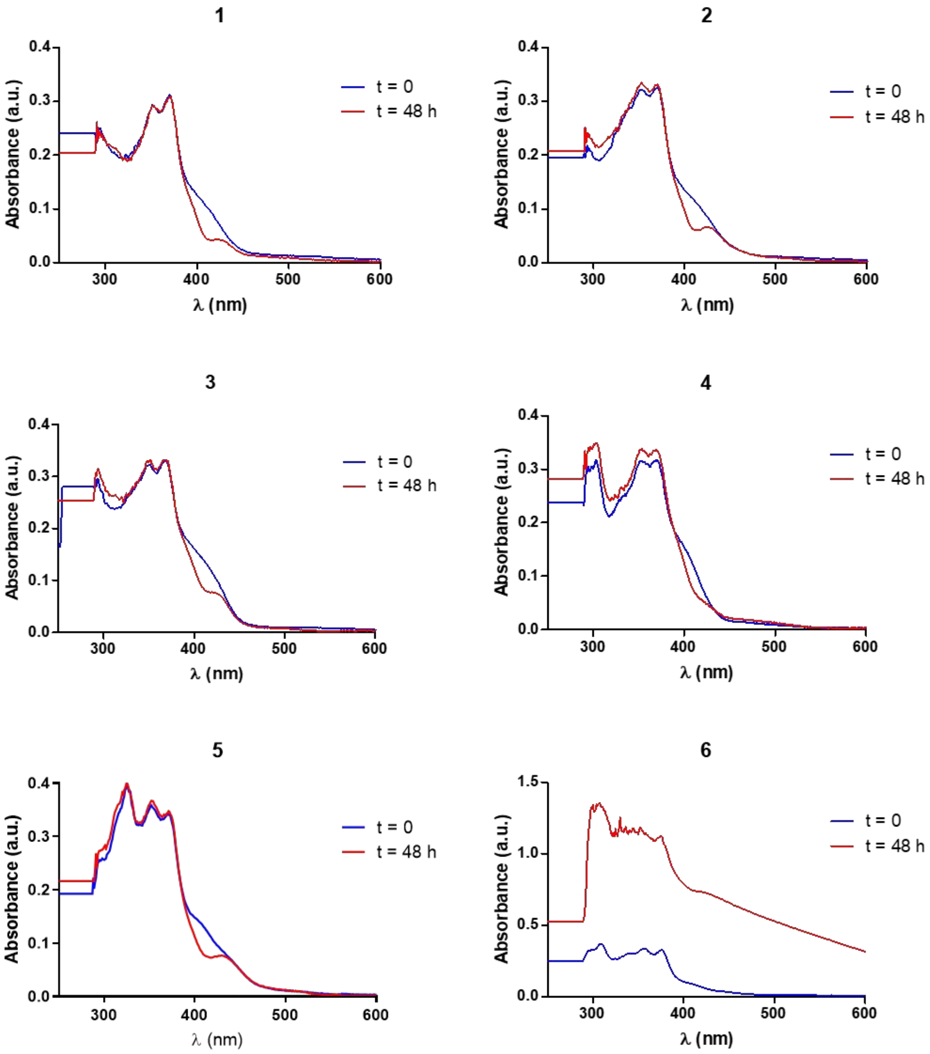

Figure S42. Monitoring of the UV/VIS absorption spectra of complexes **1-6** (10 μM) in a DMEM (+10% FBS)/DMSO (95:5) mixture before and after (t = 48 h) incubation at 37 ºC.

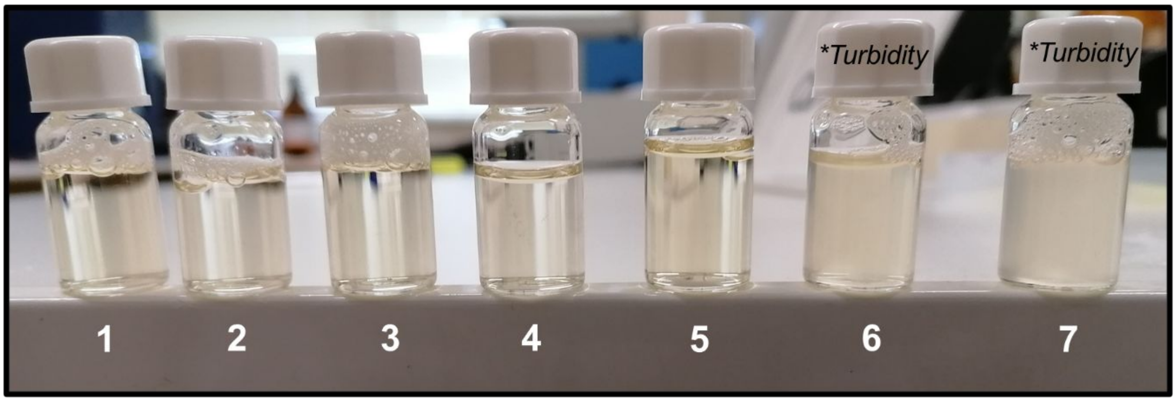

Figure S43. Picture of the glass vials containing solutions of complexes **1-7** (10 μM) in a DMEM (+10% FBS)/DMSO (95:5) mixture (48 h incubation at 37 ºC).

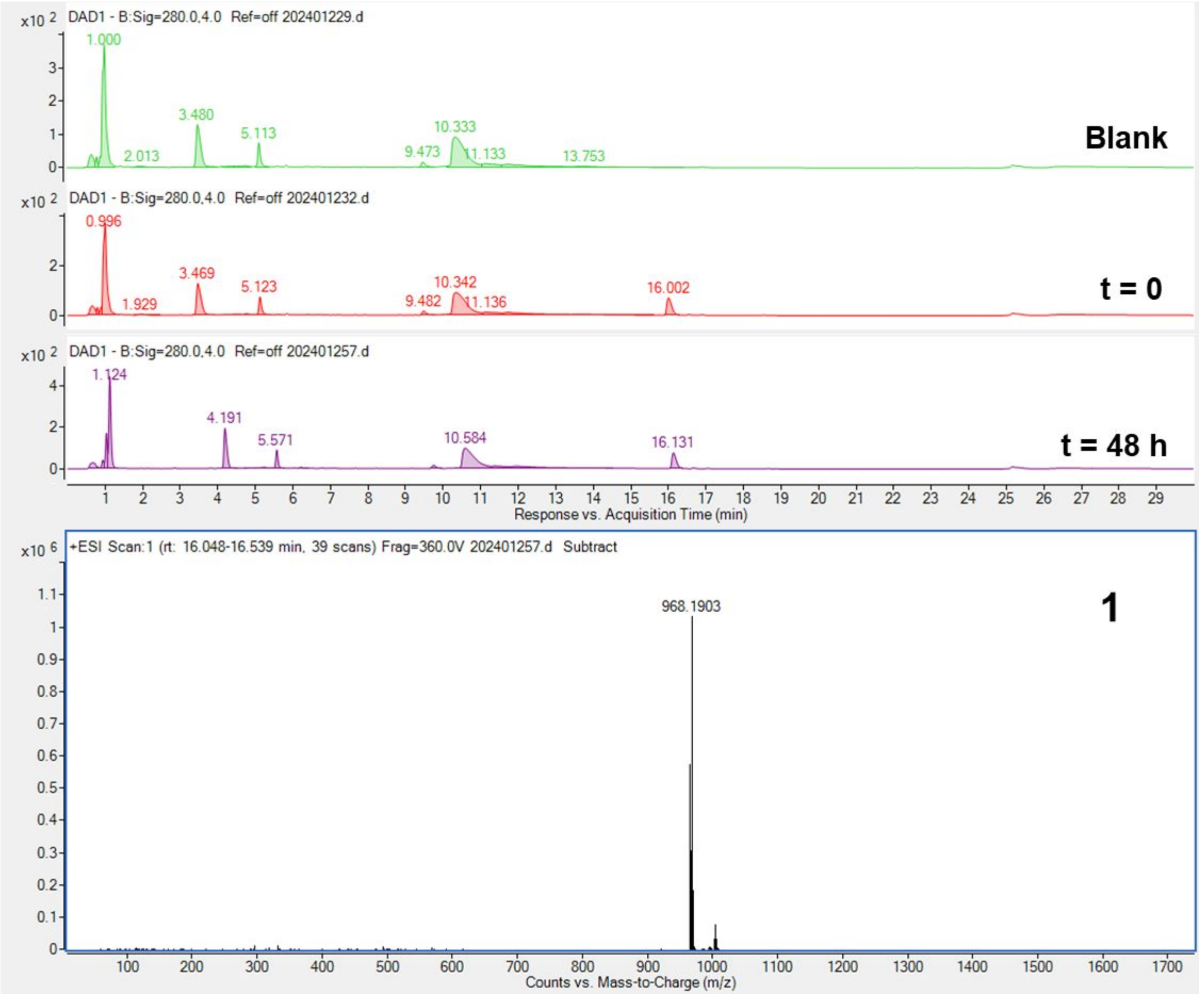

Figure S44. Stability of complex **1** (10 μM) in a DMEM (+10% FBS)/DMSO (95:5) mixture recorded by HPLC/HR-ESI-MS.

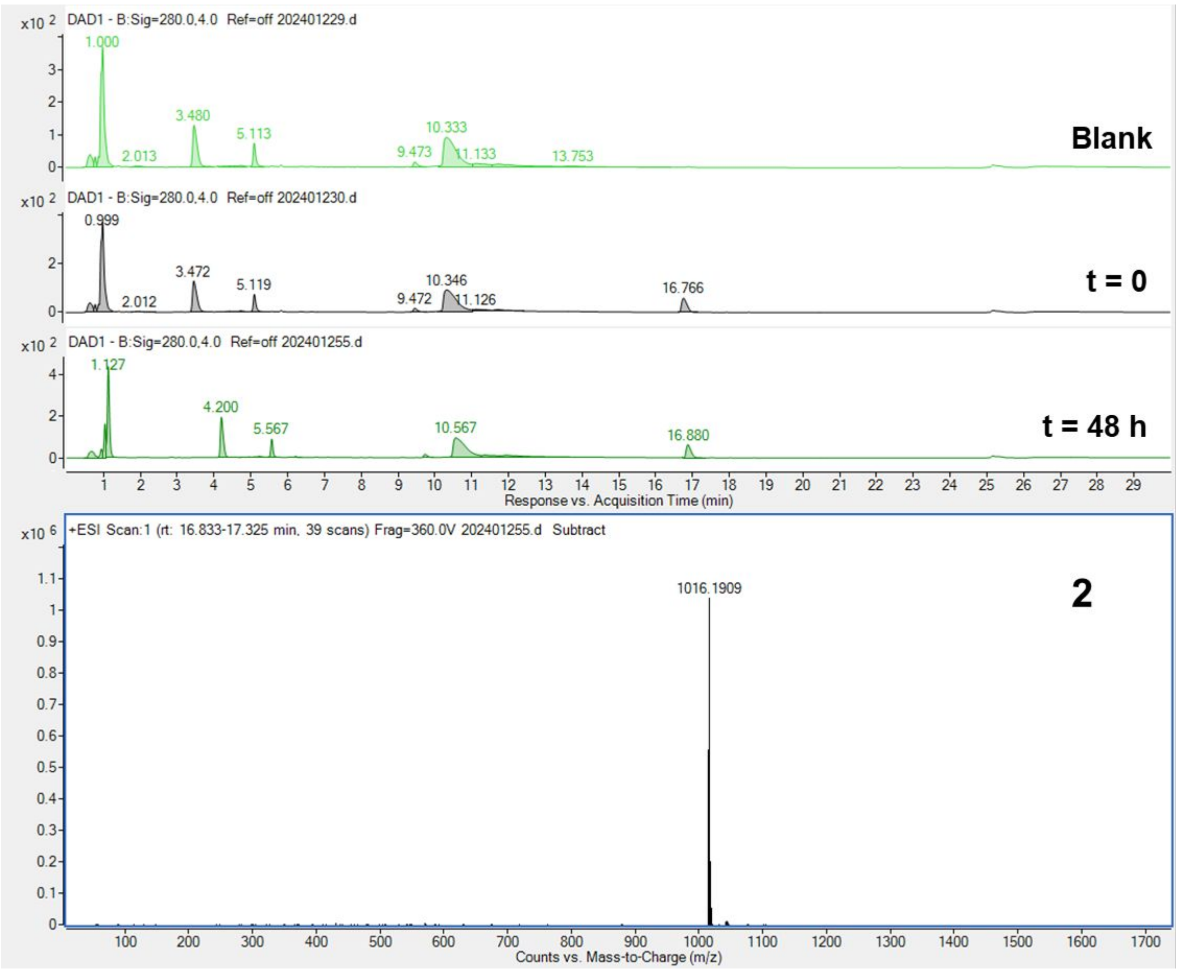

Figure S45. Stability of complex **2** (10 μM) in a DMEM (+10% FBS)/DMSO (95:5) mixture recorded by HPLC/HR-ESI-MS.

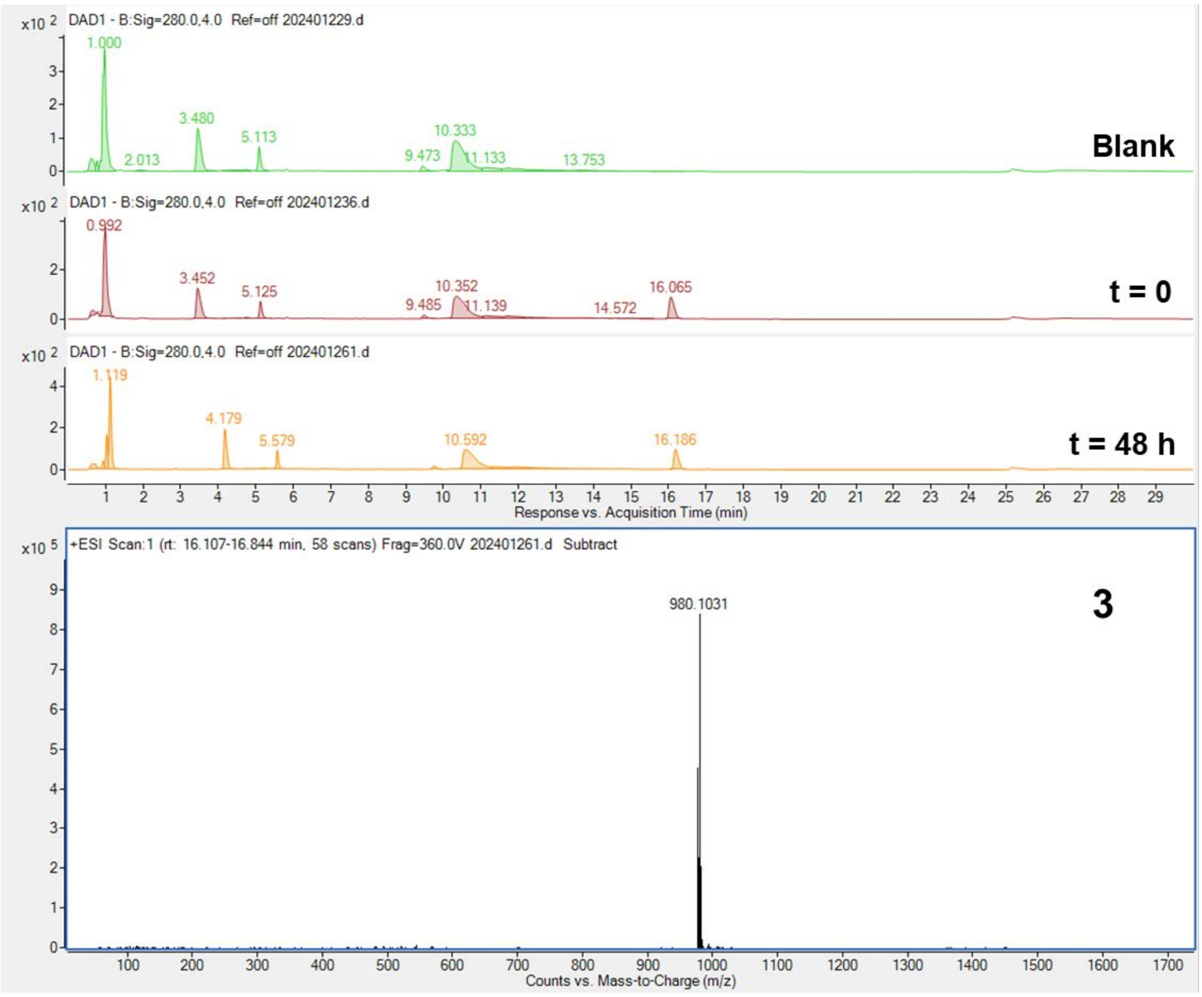

Figure S46. Stability of complex **3** (10 µM) in a DMEM (+10% FBS)/DMSO (95:5) mixture recorded by HPLC/HR-ESI-MS.

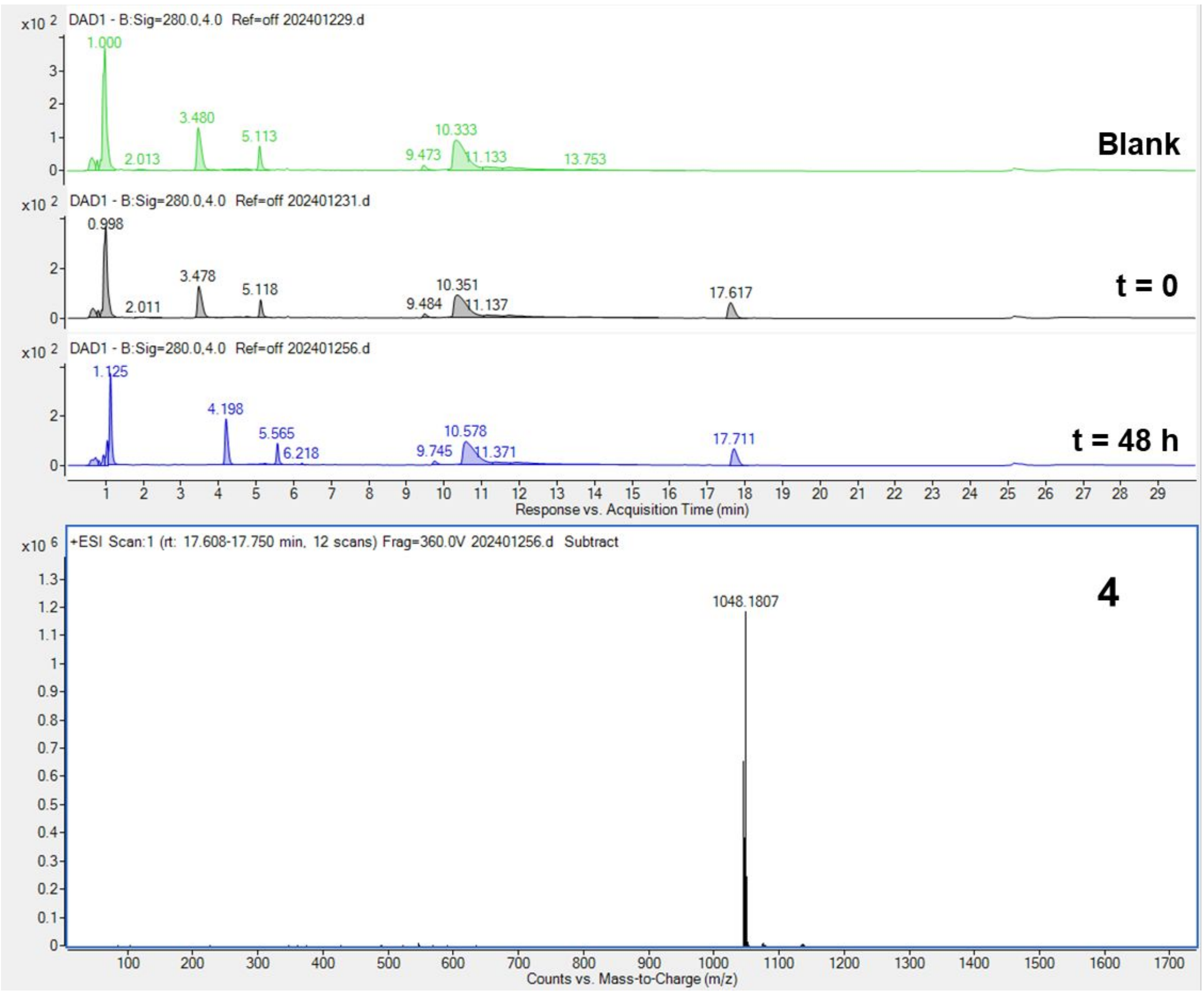

Figure S47. Stability of complex **4** (10 µM) in a DMEM (+10% FBS)/DMSO (95:5) mixture recorded by HPLC/HR-ESI-MS.

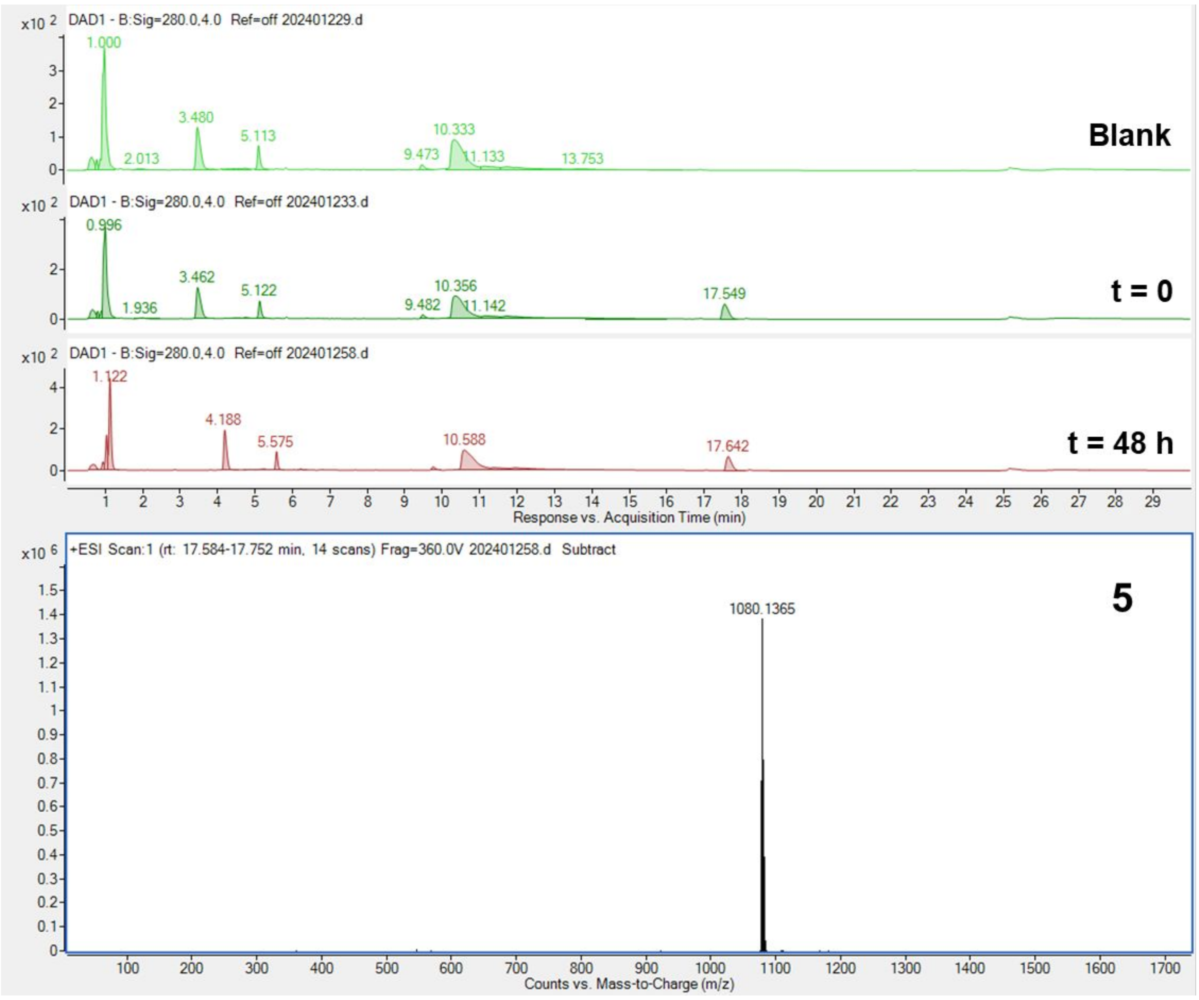

Figure S48. Stability of complex **5** (10 μM) in a DMEM (+10% FBS)/DMSO (95:5) mixture recorded by HPLC/HR-ESI-MS.

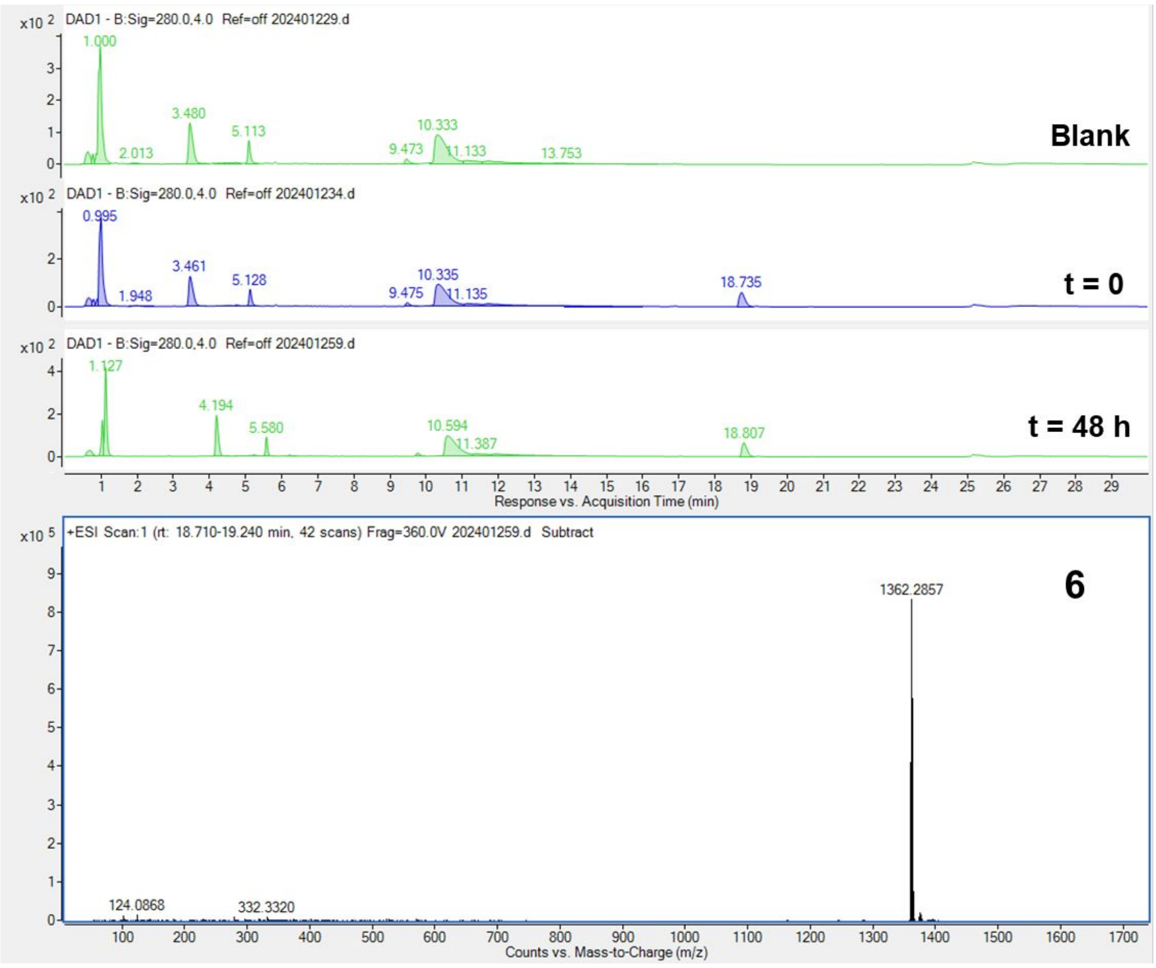

Figure S49. Stability of complex **6** (10 μM) in a DMEM (+10% FBS)/DMSO (95:5) mixture recorded by HPLC/HR-ESI-MS.

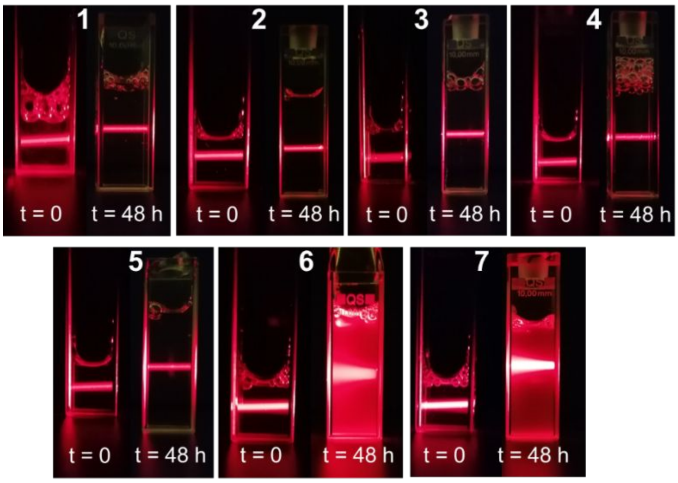

Figure S50. Tyndall effect observed in cuvettes containing solutions of **1-7** (10 μM) in a DMEM (+10% FBS)/DMSO (95:5) mixture at t = 0 and after 48 h of incubation at 37 ºC.

Table S10. Nanoparticle tracking analysis showing the size distribution and particle concentration of **1-7** aggregates in culture medium. Two independent measurements were conducted.

Complex	Mean diameter	Particles/mL
**1**	189 / 241.6	3.19 x 10^8
**2**	168.7 / 169.8	1.31 x 10^8
**3**	181.3 / 182.6	1.83 x 10^8
**4**	194.6 / 194.5	1.69 x 10^8
**5**	67 / 67.5	7.73 x 10^7
**6**	168.8 / 169.8	1.26 x 10^8
**7**	181.3 / 182.6	1.83 x 10^8

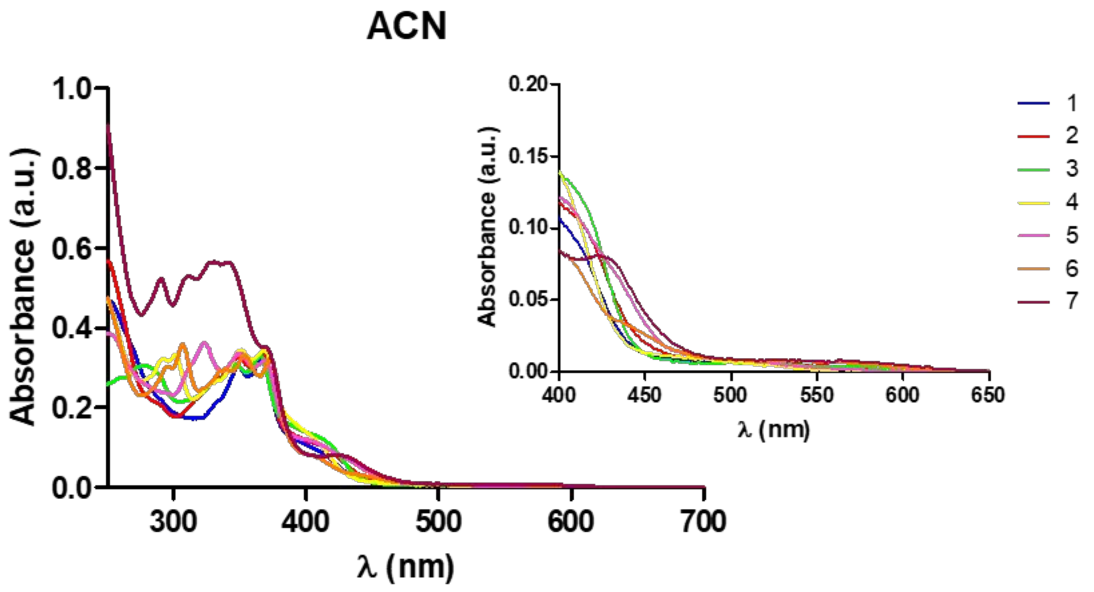

Figure S51. UV/VIS absorption spectra of complexes **1-7** (10 μM) recorded in acetonitrile.

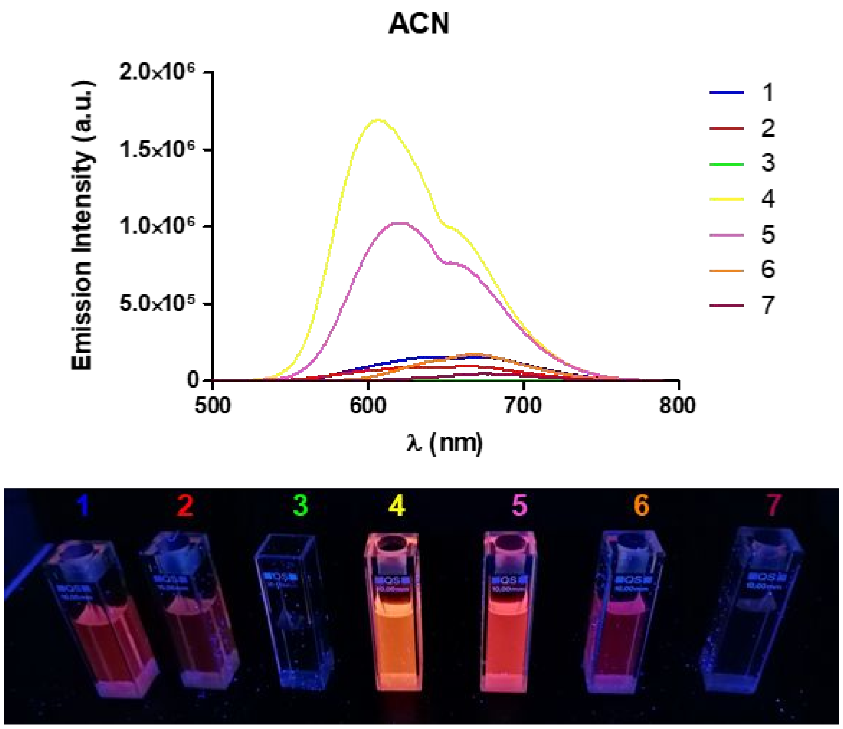

Figure S52. Emission spectra of complexes **1-7** (10 μM) recorded in CH_3CN. λ_{irrad} = 420 nm.

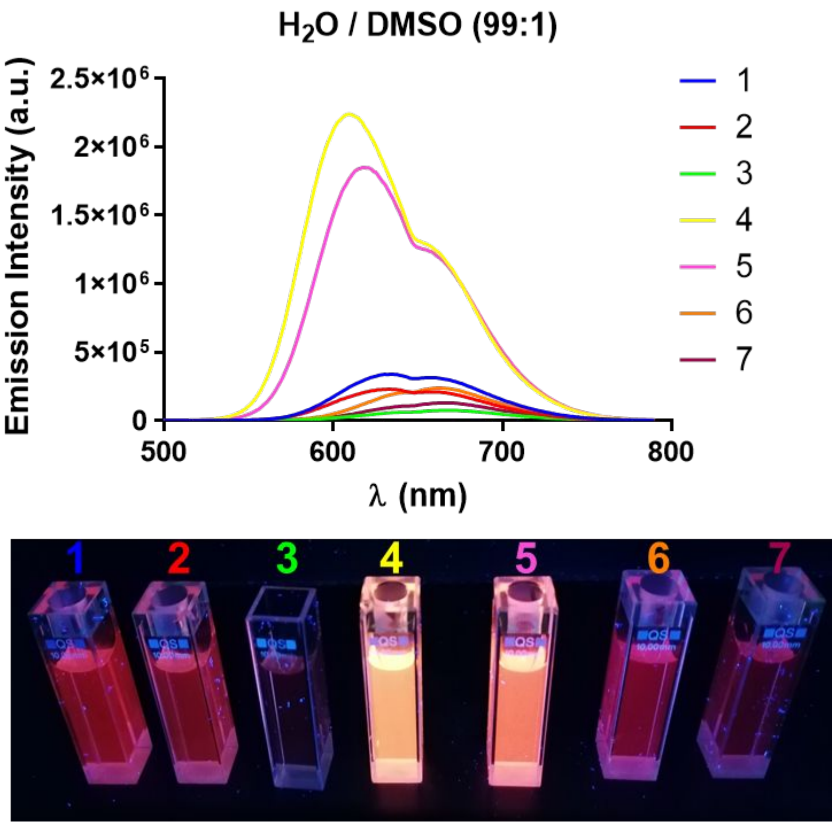

Figure S53. Emission spectra of complexes **1-7** (10 μM) recorded in a H_2O/DMSO (99:1) mixture. λ_{irrad} = 420 nm.

Table S11. Photophysical characterization of complexes **1-7** (10 μM) in acetonitrile and water (1% DMSO).

Complex	Solvent	λ_{abs} (ε, x10^3 M^{-1} cm^{-1})	λ_{exc} (nm)	λ_{em} (nm)	Φ_{PL}[a]	τ, ns (%)[b]
**1**	CH_3CN	336 (sh, 22.36), 348 (30.2), 367 (33.6), 418 (sh, 7.26)	366	633, 662	0.047	32 (9.5%)<br>158 (90.5 %)
	H_2O[a]	354 (24.93), 371 (26.33), 421 (sh, 6.76)	370	634, 660		
**2**	CH_3CN	294 (sh, 19.34), 334 (sh, 27.83), 349 (32.73), 367 (33.63), 423 (sh, 8.01)	365	635, 667	0.022	34 (18.5%)<br>119 (81.5 %)
	H_2O[a]	339 (sh, 23.94), 355 (27.7), 369 (27.19), 427 (sh, 7.45)	371	633, 660		
**3**	CH_3CN	260 (sh, 27.46), 279 (30.55), 335 (sh, 26.99), 347 (31.02), 365 (33.08), 419 (b, 10.46)	364	682	0.013	28 (25.7%)<br>77 (74.3 %)
	H_2O[a]	264 (sh, 26.22), 281 (28.41), 337 (sh, 24.72), 349 (27.35), 368 (27.81), 422 (sh, 10.25)	348	668		
**4**	CH_3CN	292 (32.32), 301 (33.25), 337 (sh, 27.5), 351 (34.5), 367 (34.35), 412 (sh, 10.21)	367	606, 660	0.135	58 (12.9%)<br>292 (87.1%)
	H_2O[a]	294 (25.82), 302 (26.29), 333 (sh, 21.23), 354 (26.95), 369 (26.37), 412 (sh, 9.59)	370	610, 663		
**5**	CH_3CN	323 (36.2), 348 (33.46), 369 (31.86), 410 (sh, 11.0)	350	620, 660	0.096	40 (7.9%)<br>213 (92.1%)
	H_2O[a]	327 (32.26), 355 (29.84), 372 (28.11), 430 (sh, 7.88)	372	618, 661		
**6**	CH_3CN	296 (30.29), 307 (35.88), 339 (29.8), 353 (33.25), 374 (31.9), 415 (sh, 6.3)	372	668	0.042	35 (5.5%)<br>214 (94.5%)
	H_2O[a]	298 (28.72), 309 (32.55), 344 (sh, 26.44), 360 (28.33), 378 (26.81), 417 (sh, 6.24)	372	662		
**7**	CH_3CN	290, (0.5224), 311 (sh, 53.1), 330 (56.51), 346 (54.65), 373 (sh, 33.4), 431 (sh, 7.74)	371	678	0.025	31 (18.7%)<br>111 (81.3%)
	H_2O[a]	292 (40.28), 315 (sh, 42.05), 333 (43.95), 346 (43.77), 376 (sh, 26.62), 438 (sh, 6.04)	375	666		

[a] 1% DMSO. [b] Determined for deaerated H_2O-DMSO (99:1) mixtures.

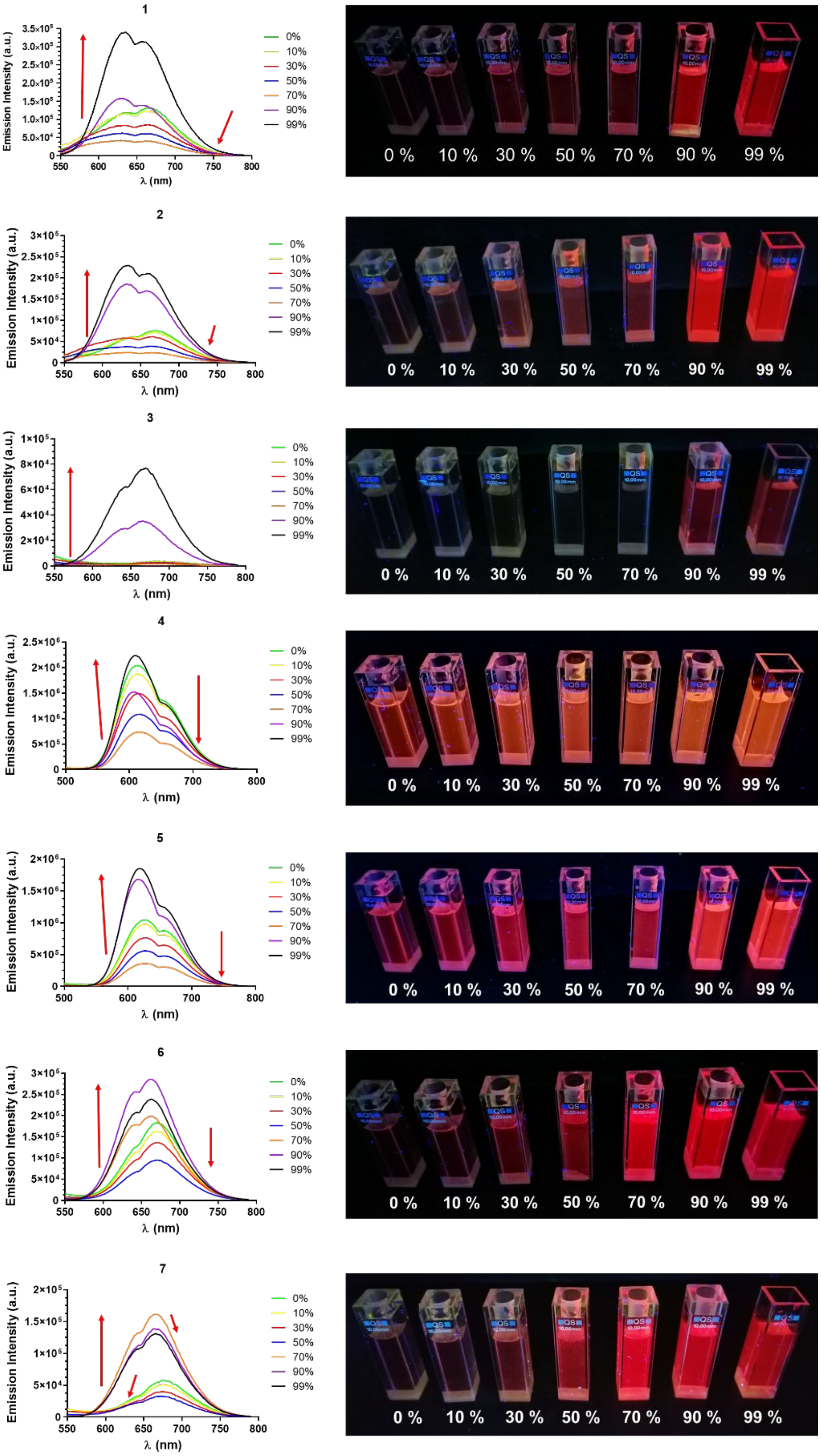

Figure S54. Monitoring of the emission spectra (λ_{irrad} = 420 nm) of complexes **1-7** (10 µM) while increasing the water fraction. Pictures of the cuvettes containing the aqueous solutions upon light irradiation with a short-wavelength lamp.

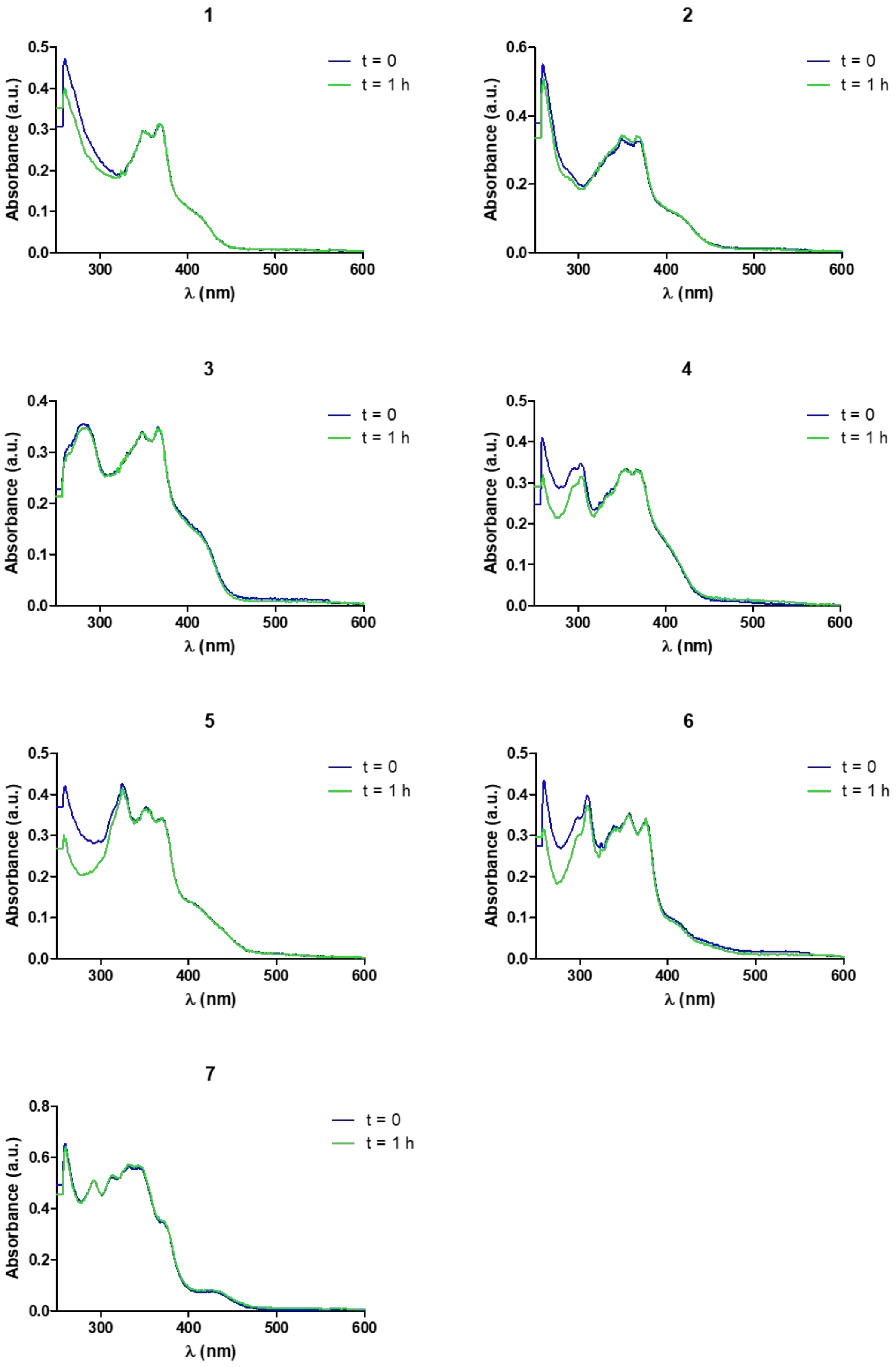

Figure S55. Photostability of complexes **1-7** (10 μM) in DMSO monitored by UV/VIS absorption spectroscopy. Samples were irradiated for 1 h with a blue (λ_{irrad} = 465 nm, 5 mW/cm^2) lamp.

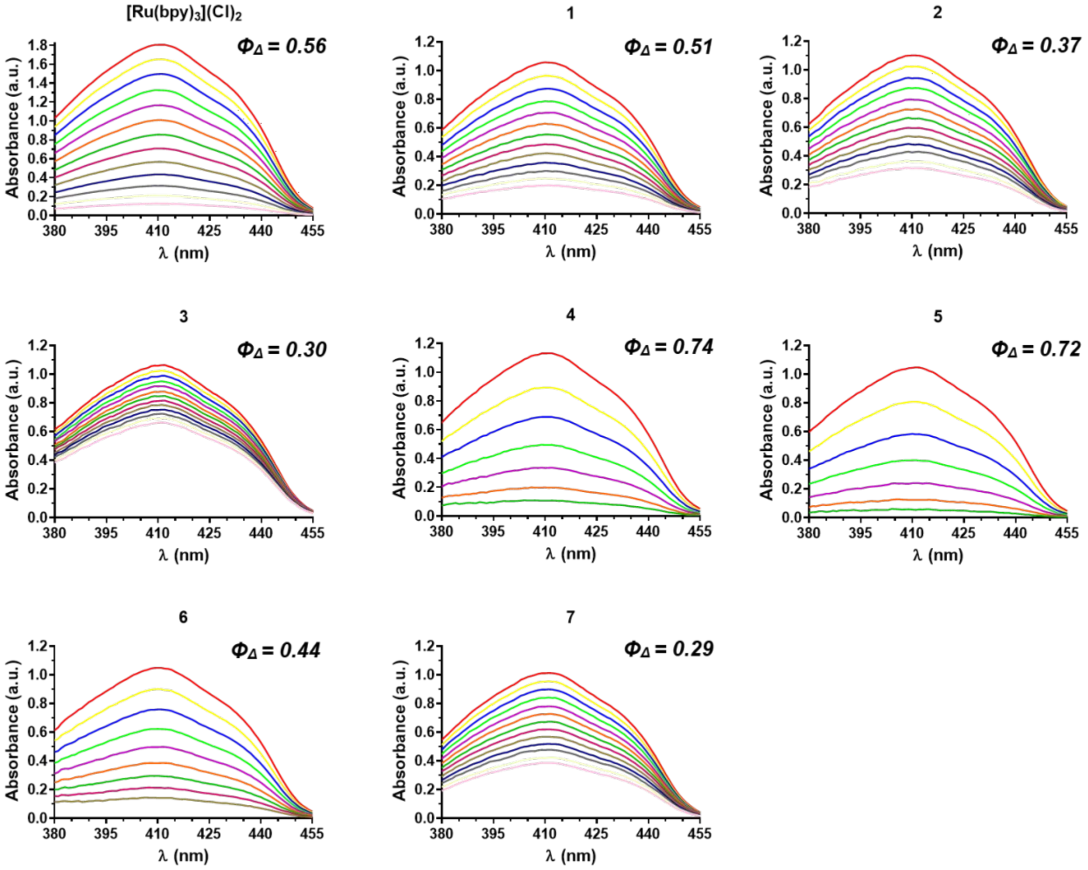

Figure S56. Absorption profile of 1,3-diphenylisobenzofuran after incubation with [Ru(2,2'-bipyridine)$_3$]$^{2+}$ (5 μM) and complexes **1-7** (30-50 μM) and exposure to blue (λ_{irrad} = 465 nm, 0.4 mW/cm^2) light irradiation for 15 second intervals.

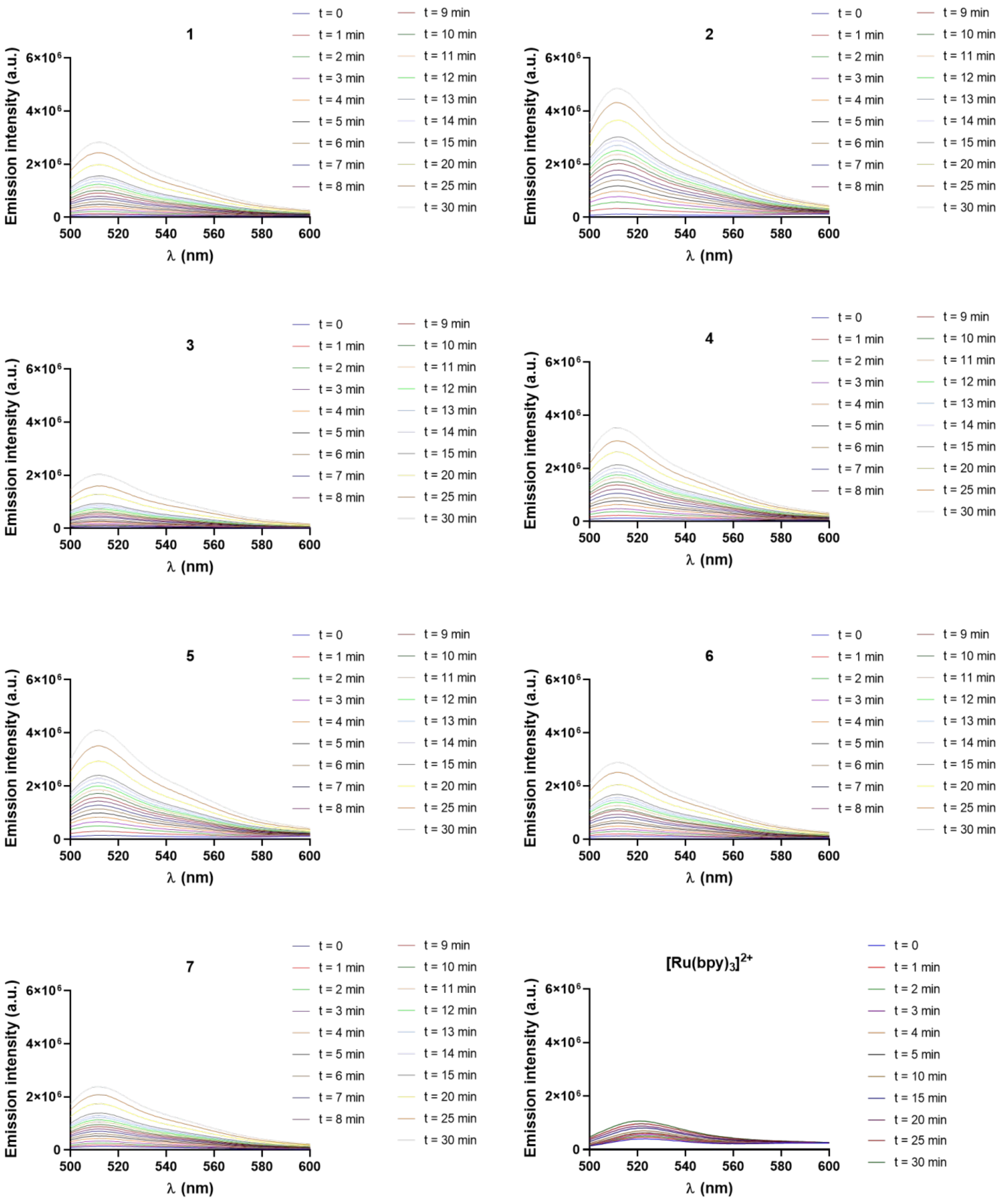

Figure S57. Time-dependent monitoring of the fluorescence spectra of HPF (10 μM) upon incubation with complexes **1-7** (10 μM) in a H_2O/DMF (95:5) mixture and irradiation with a blue lamp (λ_{irrad} = 465 nm, 5 mW/cm^2) for short intervals.

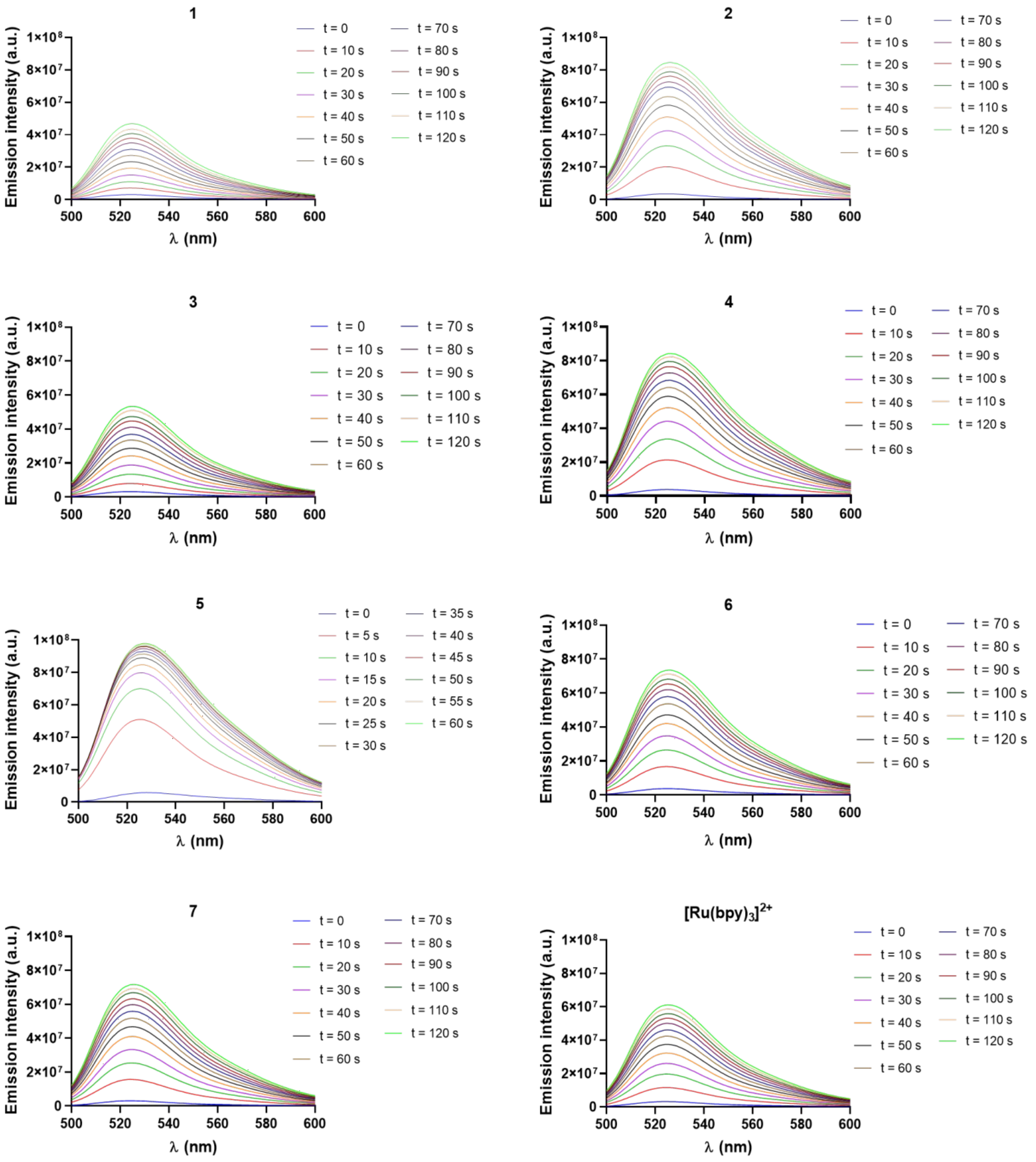

Figure S58. Time-dependent monitoring of the fluorescence spectra of DHR123 (10 μM) upon incubation with complexes **1-7** (10 μM) in a H_2O/DMF (95:5) mixture and irradiation with a blue lamp (λ_{irrad} = 465 nm, 5 mW/cm^2) for short intervals.

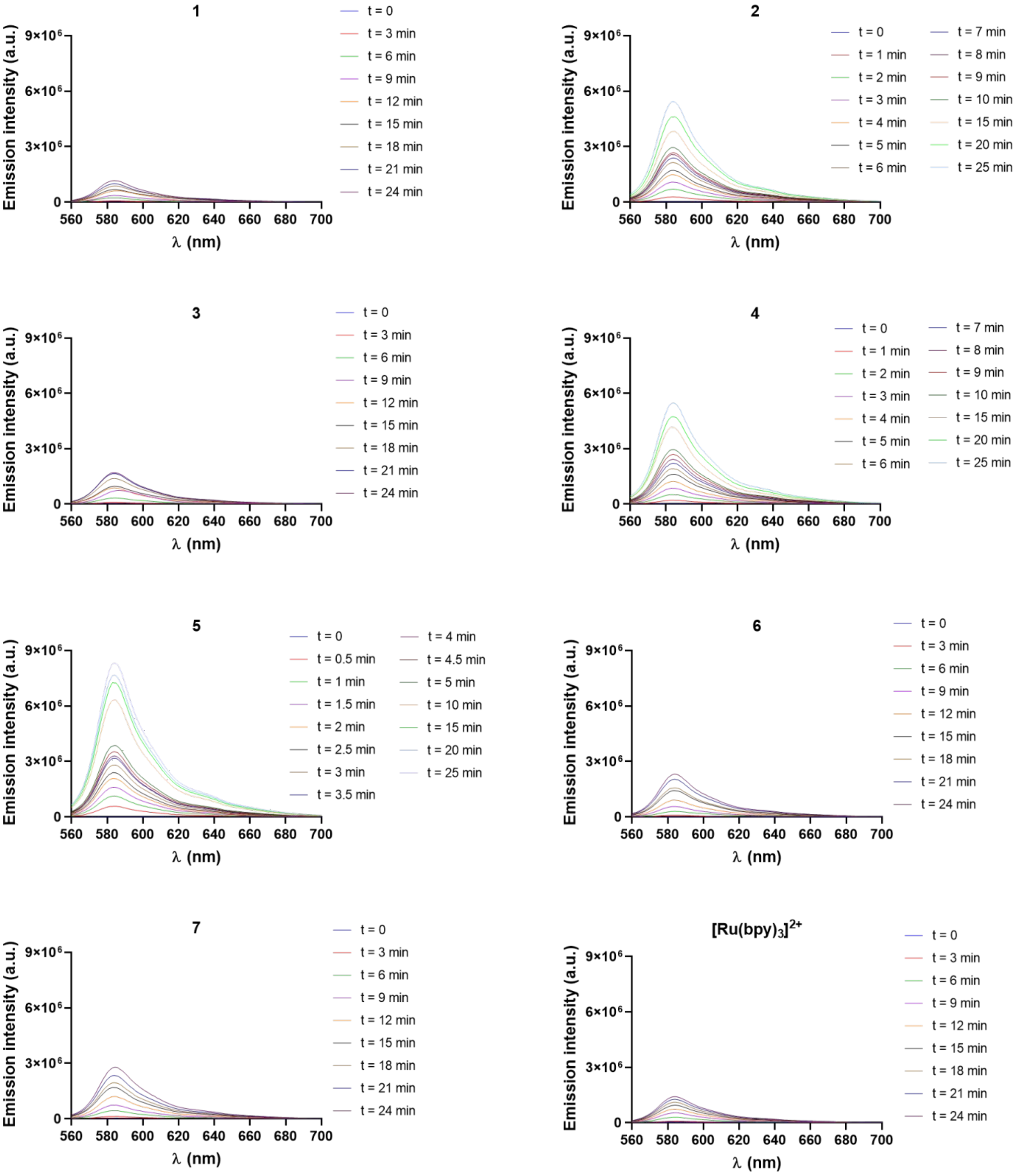

Figure S59. Time-dependent monitoring of the fluorescence spectra of Amplex Red (10 μM) upon incubation with complexes **1-7** (5 μM) in a H_2O/DMF (95:5) mixture and irradiation with a blue lamp (λ_{irrad} = 465 nm, 5 mW/cm^2) for short intervals.

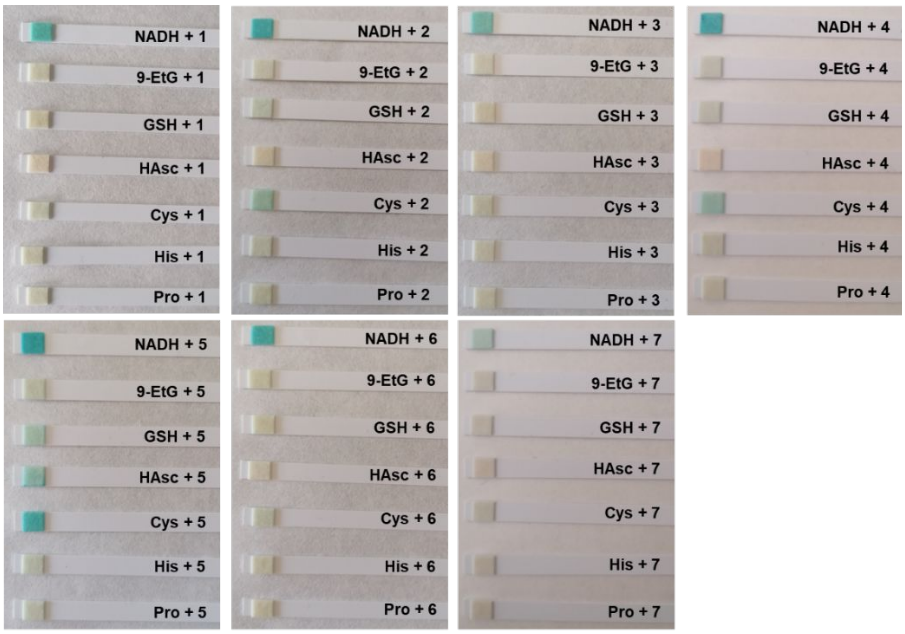

Figure S60. Hydrogen peroxide strips dipped into irradiated (λ_{irrad} = 465 nm, 5 mW/cm^2, 1 h) solutions of relevant biomolecules (1 mM) in the presence of complexes **1-7** (10 µM) in a H_2O/DMF (95:5) mixture.

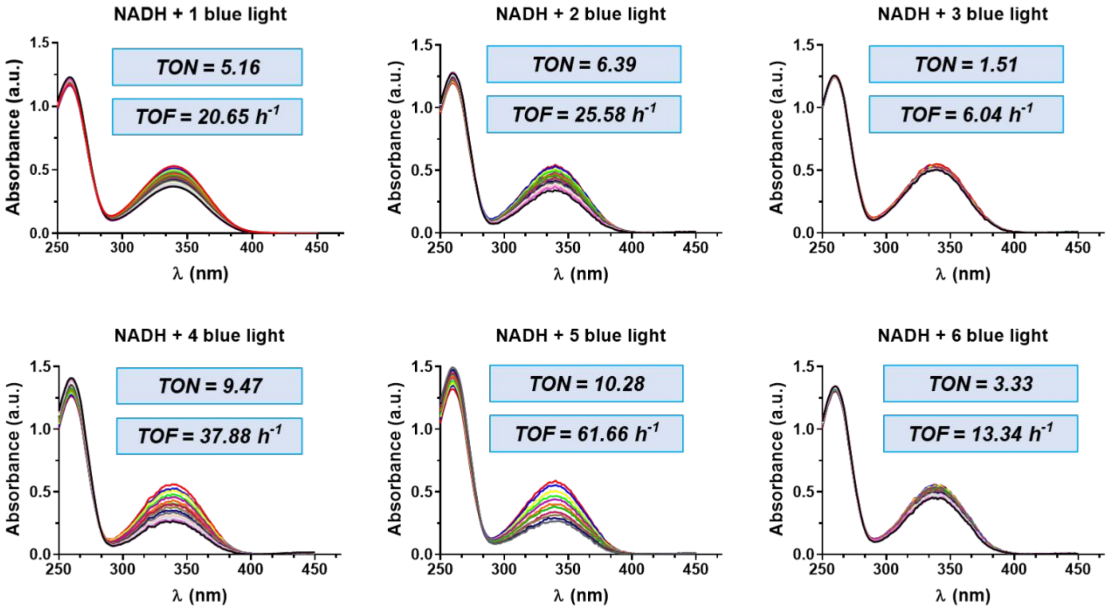

Figure S61. Monitoring of the UV/VIS absorption profile of NADH (100 µM) upon incubation with complexes **1-6** (5 µM) in a H_2O/DMF (95:5) mixture and exposure to blue light irradiation (λ_{irrad} = 465 nm, 5 mW/cm^2, 10-15 min).

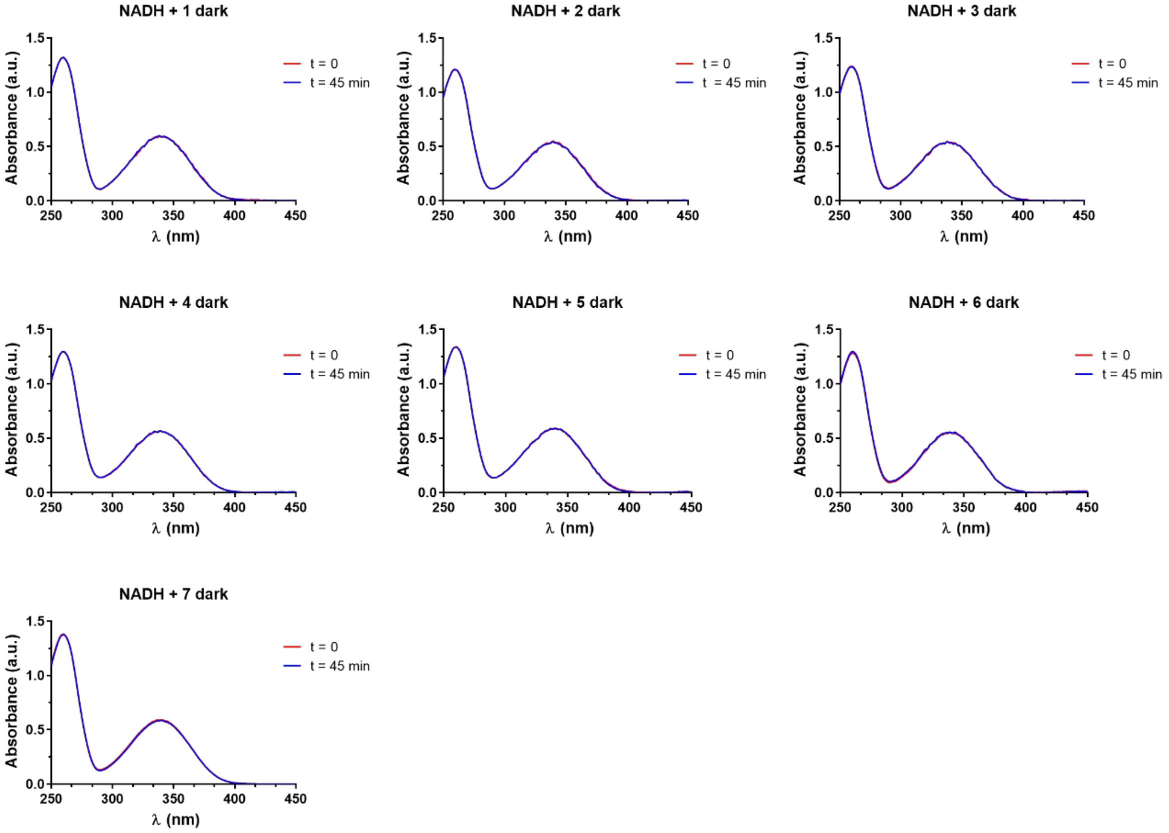

Figure S62. Monitoring of the absorption profile of NADH (100 μM) upon incubation for 45 min with complexes **1-7** (5 μM) in a H_2O/DMF (95:5) mixture in the dark.

Table S12. Singlet oxygen production quantum yield (Φ_Δ) and NADH photooxidation TOF (h^{-1}) values obtained for complexes **1-7**.

Complex	Φ_Δ ($^1O_2$)	TOF (NADH, h^{-1})
**1**	0.51	20.65
**2**	0.37	25.58
**3**	0.30	6.04
**4**	0.74	37.88
**5**	0.72	61.66
**6**	0.44	13.34
**7**	0.29	124.46
**$[Ru(bpy)_3](Cl)_2$**	0.56	-

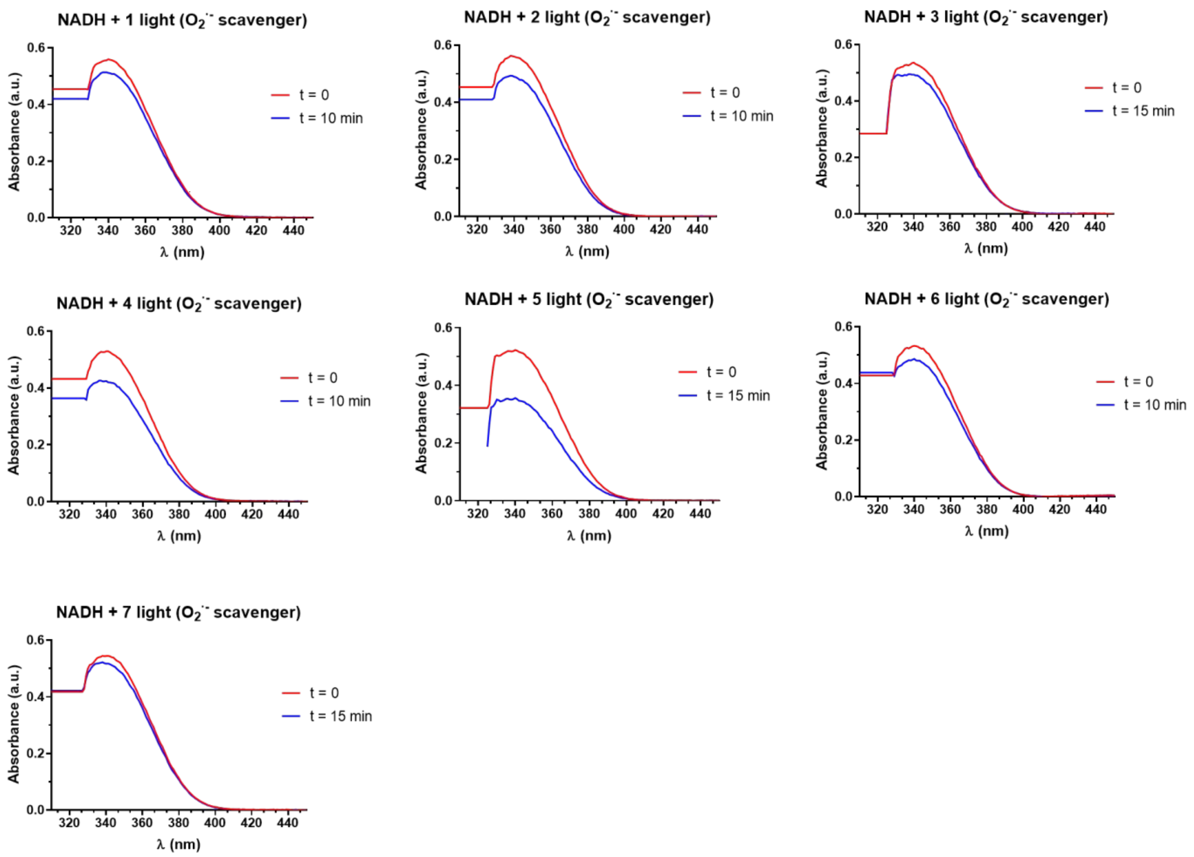

Figure S63. Monitoring of the UV/VIS absorption profile of NADH (100 µM) upon incubation with complexes **1-7** (5 µM) and tiron (10 mM) in a H_2O/DMF (95:5) mixture and exposure to blue light irradiation (λ_{irrad} = 465 nm, 5 mW/cm^2, 10-15 min).

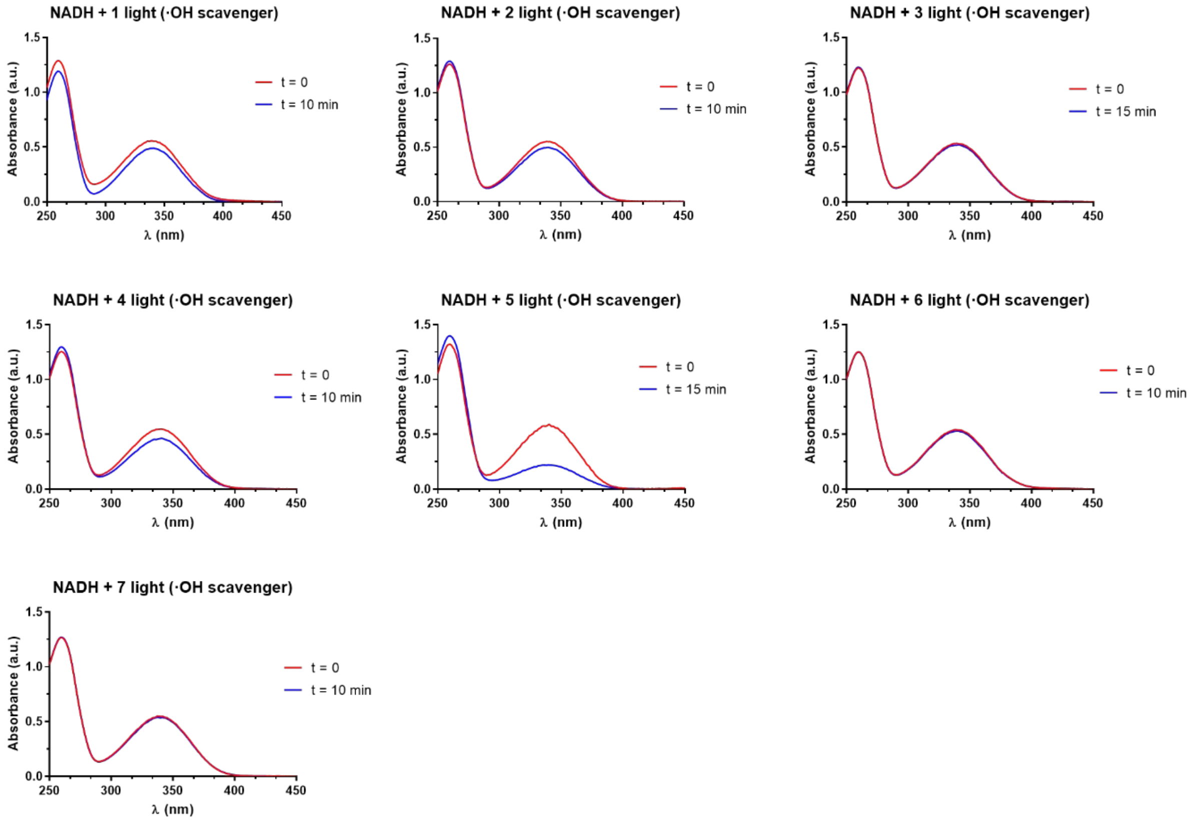

Figure S64. Monitoring of the UV/VIS absorption profile of NADH (100 µM) upon incubation with complexes **1-7** (5 µM) and mannitol (20 mM) in a H_2O/DMF (95:5) mixture and exposure to blue light irradiation (λ_{irrad} = 465 nm, 5 mW/cm^2, 10-15 min).

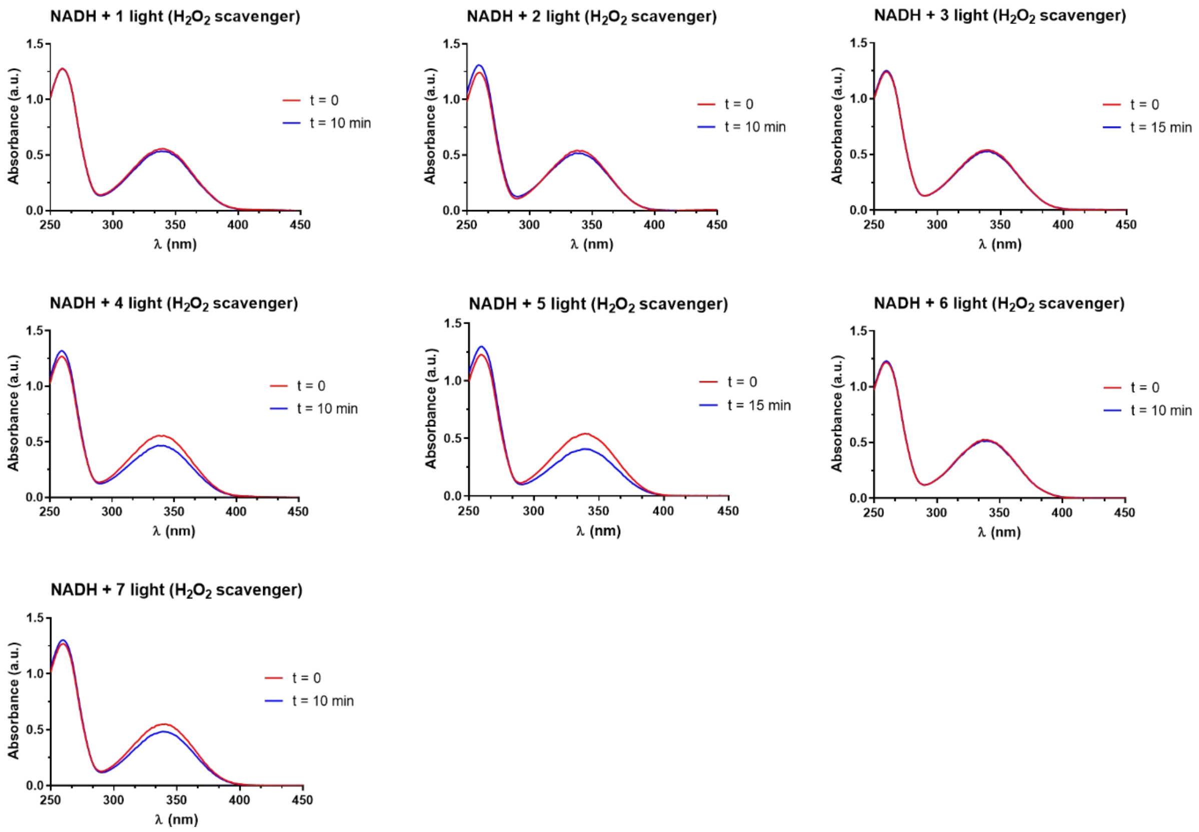

Figure S65. Monitoring of the UV/VIS absorption profile of NADH (100 µM) upon incubation with complexes **1-7** (5 µM) and sodium pyruvate (10 mM) in a H_2O/DMF (95:5) mixture and exposure to blue light irradiation (λ_{irrad} = 465 nm, 5 mW/cm^2, 10-15 min).

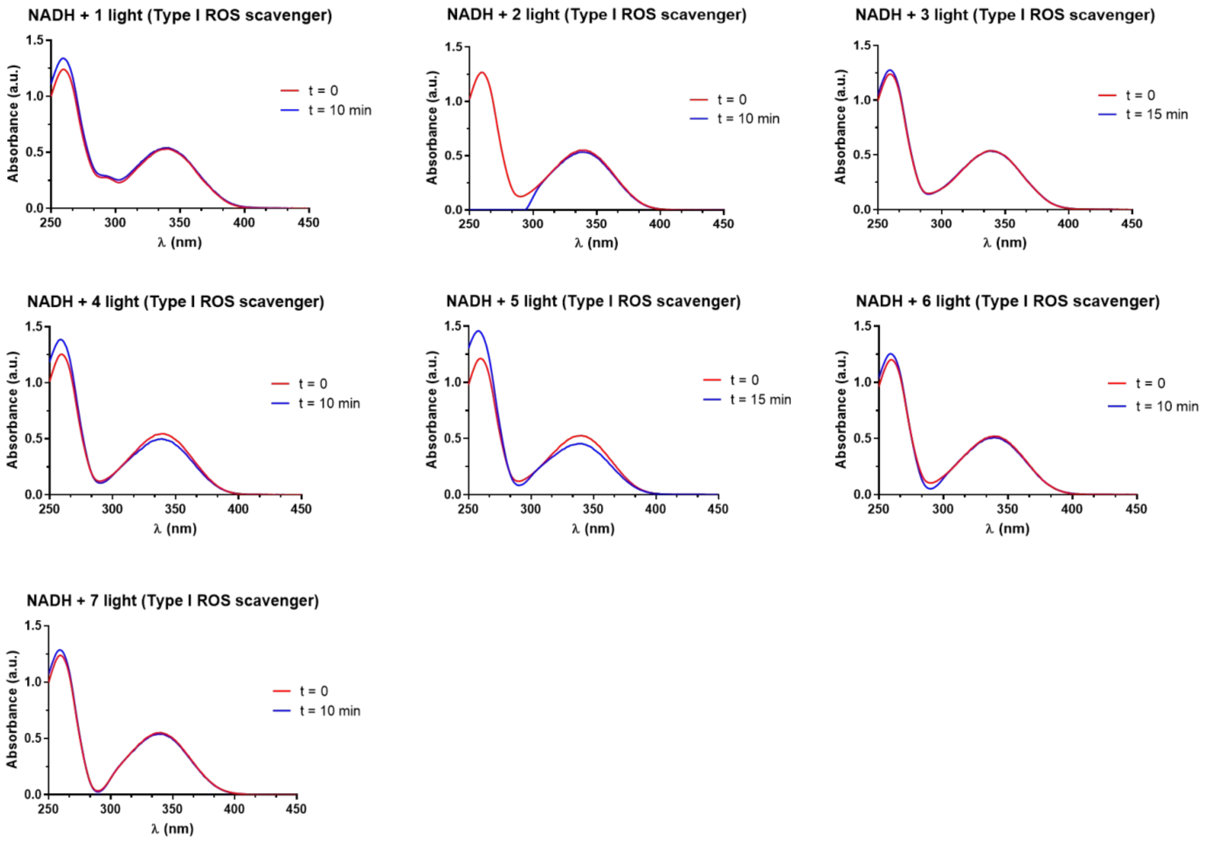

Figure S66. Monitoring of the UV/VIS absorption profile of NADH (100 μM) upon incubation with complexes **1-7** (5 μM) and trolox (0.1 mM) in a H_2O/DMF (95:5) mixture and exposure to blue light irradiation (λ_{irrad} = 465 nm, 5 mW/cm^2, 10-15 min).

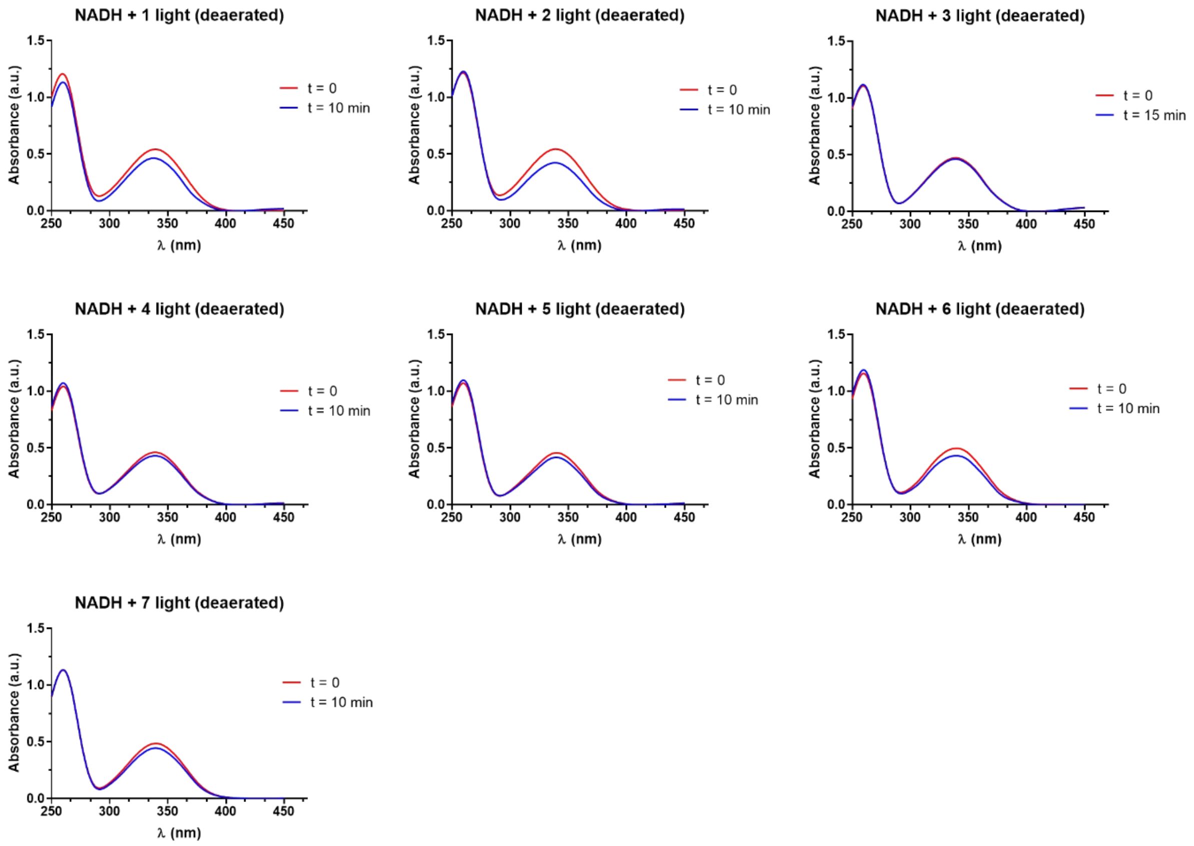

Figure S67. Monitoring of the UV/VIS absorption profile of NADH (100 μM) upon incubation with complexes **1-7** (5 μM) in a dearated H_2O/DMF (95:5) mixture and exposure to blue light irradiation (λ_{irrad} = 465 nm, 5 mW/cm^2, 10-15 min).

Table S13. The antiproliferative activity (IC_{50} values[a]) of the investigated complexes determined in A549 cells under hypoxic conditions (1% O_2).[b,c]

	Dark	Irradiated	PI
**1**	14 ± 2	1.9 ± 0.3	7.2
**2**	10.3 ± 0.8	3.9 ± 0.5	2.6
**3**	13 ± 2	1.5 ± 0.2	8.6
**4**	9 ± 1	0.37 ± 0.07	23.5
**5**	8.2 ± 0.7	1.0 ± 0.2	8.3
**6**	16 ± 3	8.0 ± 0.9	1.9
**7**	≥100	≥100	Nd

[a] Concentration that causes 50% inhibition of cell proliferation. [b] Cells were treated for 60 min with increasing concentrations of the investigated complexes, followed by 60 min of irradiation with blue light (420 nm; 58 W/m^2) and 70 h of drug-free incubation. The entire incubation was conducted under hypoxic conditions (1% O_2). IC_{50} values were determined by MTT assay. [d] PI - phototoxicity index was calculated as the ratio of IC_{50} determined under the dark conditions / IC_{50} determined for 420 nm irradiated samples.

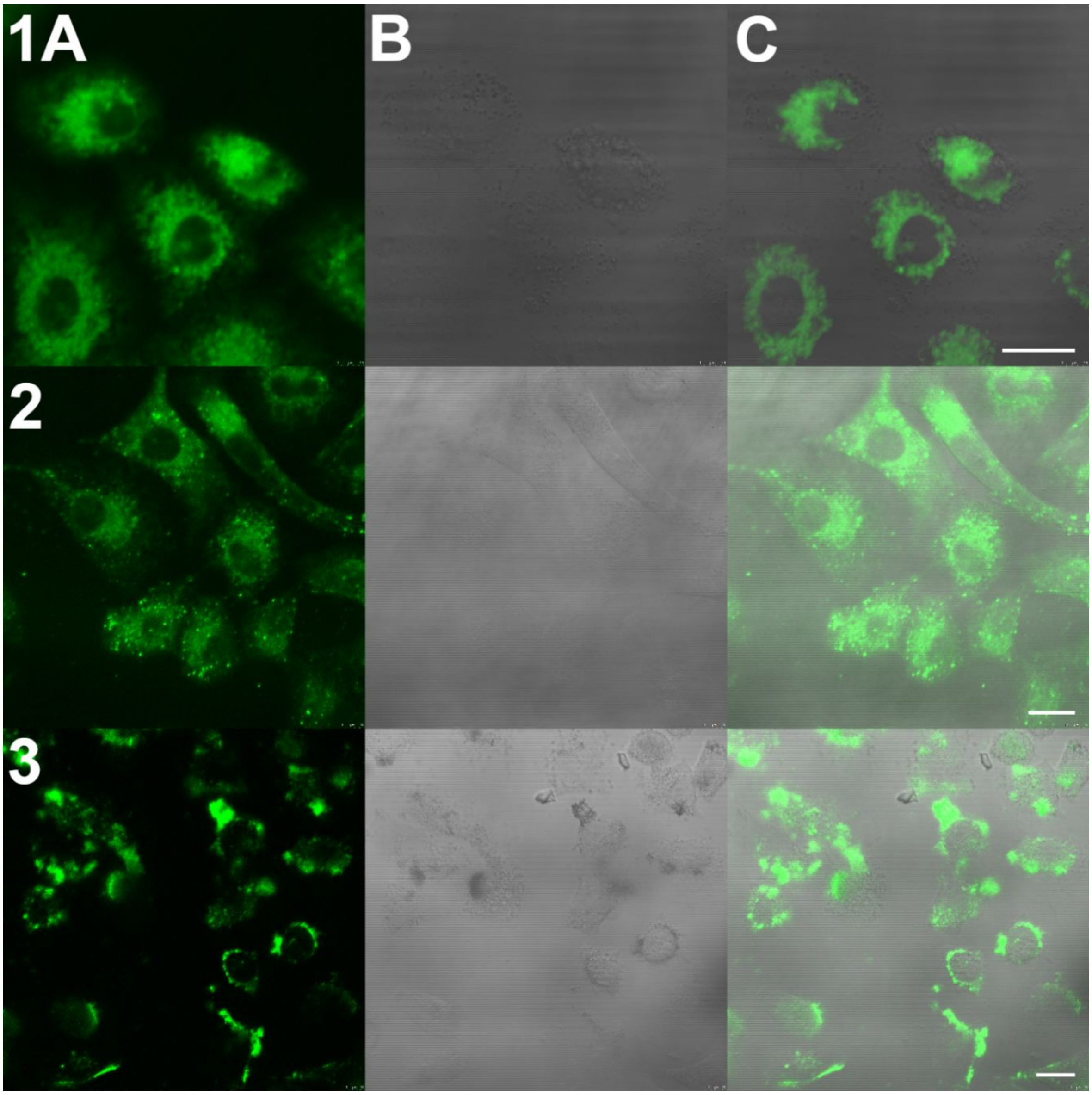

Figure S68. Localization of tested compounds in A549 cells using confocal microscopy. Panels show: A) Fluorescence channel indicating compound localization; B) Bright-field channel; C) Overlay of fluorescence and bright-field channels. Complexes **4** (Panel 1), **5** (Panel 2), and **7** (Panel 3) were tested. A549 cells were treated for 2 h with 5 μM of each compound. Scale bars represent 20 μm.

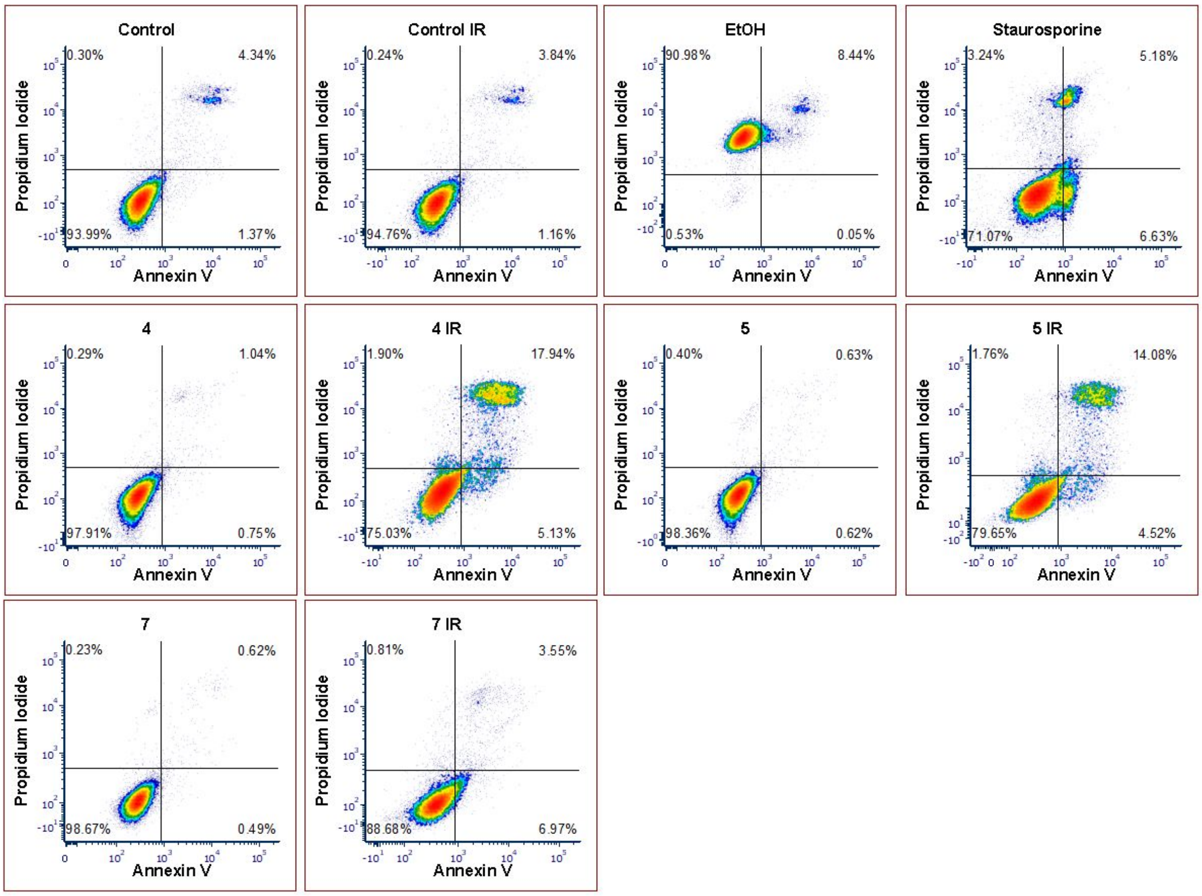

Figure S69. Investigation of cell death mechanisms in A549 cells following photodynamic treatment. A549 cells were exposed to compounds at their IC_{50} concentrations for a total of 2 h, comprising a 1-h dark incubation followed by 1 h of 420 nm blue light irradiation. The mode of cell death was subsequently assessed 18 h post-treatment via flow cytometry, employing propidium iodide to detect necrosis and Annexin V to detect apoptosis.

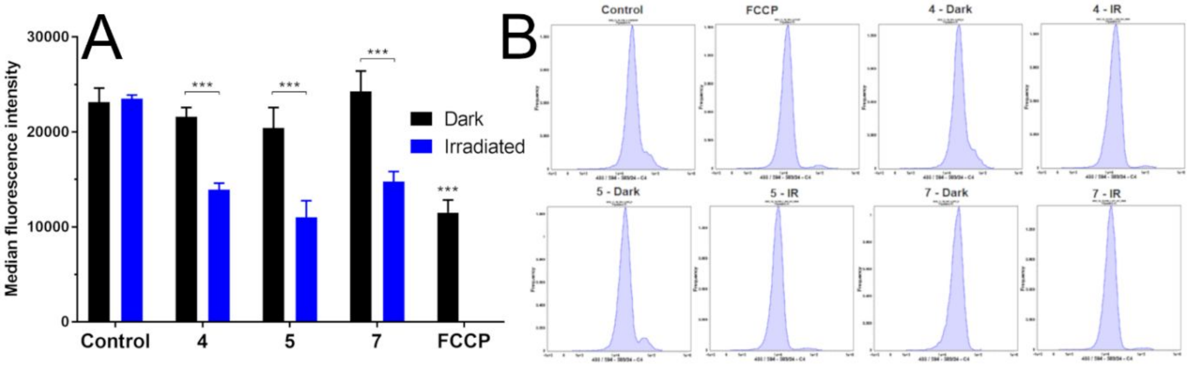

Figure S70. Photodynamic treatment induces mitochondrial membrane potential ($\Delta\psi_m$) loss in A549 cells. (A) Quantitative analysis of TMRE fluorescence intensity. (B) Representative flow cytometry histograms showing MMP distribution. Cells were treated with compounds at IC_{50} concentrations for 2 h (1 h dark incubation followed by 1 h of 420 nm light irradiation). MMP was assessed 18 h post-treatment using TMRE staining. FCCP was employed as a positive control for mitochondrial depolarization. Data represent the mean ±SD of three independent experiments performed in duplicate. ***p≤0.001 compared to treated, dark incubated cells.

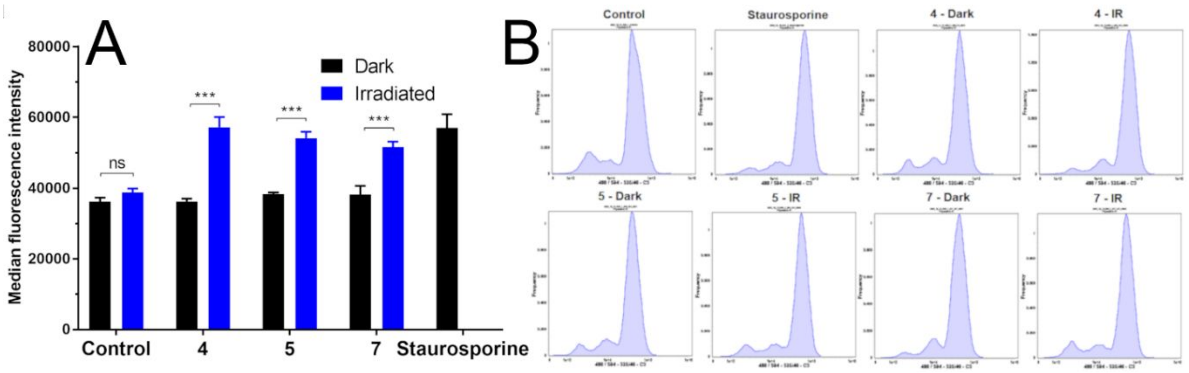

Figure S71. Caspase-3/7 activation in A549 cells following photodynamic treatment. (A) Quantitative analysis of fluorescence intensity using the CellEvent™ Caspase-3/7 Green Detection Reagent. (B) Representative flow cytometry histograms showing fluorescence intensity shifts indicative of caspase-3/7 activation. A549 cells were treated with the indicated compounds at their IC_{50} concentrations for 2 h (1 h dark incubation followed by 1 h irradiation at 420 nm). Staurosporine (1 µM, 4 h) served as a positive control. Data represent the mean ± SD of three independent experiments performed in duplicate. ***p ≤ 0.001 compared to treated, dark-incubated cells.

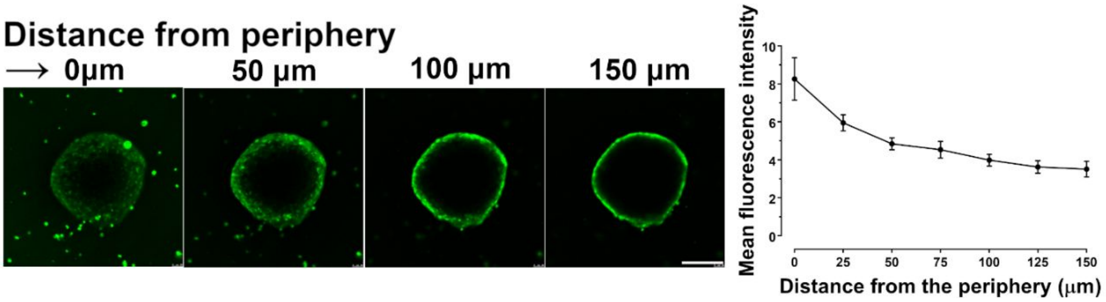

Figure S72. Penetration of compound 4 to HCT-116 spheroids. Z-stack images were acquired by using confocal microscopy. Scale bar represents 200 μm. The graph depicts mean fluorescence intensity from the periphery to the core of spheroids. Error bars represent the standard deviation from two independent experiments.

2. REFERENCES

(1) Spek, A. L. Structure Validation in Chemical Crystallography. *Acta Crystallogr. D Biol. Crystallogr.* **2009**, *65*, 148–155.

(2) Spek, A. L. A Multipurpose Crystallographic Tool. *Utrecht Univ. Utrecht Neth.* **2005**.

(3) Yang, X.-J.; Drepper, F.; Wu, B.; Sun, W.-H.; Haehnel, W.; Janiak, C. From Model Compounds to Protein Binding: Syntheses, Characterizations and Fluorescence Studies of $[Ru^{II}(Bipy)(Terpy)L]^{2+}$ Complexes (Bipy = 2,2′-Bipyridine; Terpy = 2,2′:6′,2″-Terpyridine; L = Imidazole, Pyrazole and Derivatives, Cytochrome c). *Dalton Trans* **2005**, *2*, 256–267.

(4) Janiak, C. A Critical Account on π–π Stacking in Metal Complexes with Aromatic Nitrogen-Containing Ligands. *J. Chem. Soc. Dalton Trans.* **2000**, *21*, 3885–3896.

(5) Nishio, M. The CH/π Hydrogen Bond in Chemistry. Conformation, Supramolecules, Optical Resolution and Interactions Involving Carbohydrates. *Phys. Chem. Chem. Phys.* **2011**, *13*, 13873–13900.

(6) Nishio, M.; Umezawa, Y.; Honda, K.; Tsuboyama, S.; Suezawa, H. CH/π Hydrogen Bonds in Organic and Organometallic Chemistry. *CrystEngComm* **2009**, *11*, 1757.

(7) Nishio, M. CH/π Hydrogen Bonds in Crystals. *CrystEngComm* **2004**, *6*, 130.

(8) Janiak, C.; Temizdemir, S.; Dechert, S.; Deck, W.; Girgsdies, F.; Heinze, J.; Kolm, M. J.; Scharmann, T. G.; Zipffel, O. M. Binary [Hydrotris(indazol-1-yl)borato]metal Complexes, M(Tp4Bo)2[1] with M = Fe, Co, Ni, Cu, and Zn: Electronic Properties and Solvent-Dependent Framework Structures through C−H···π Interactions. *Eur. J. Inorg. Chem.* **2000**, *2000*, 1229–1241.

(9) Umezawa, Y.; Tsuboyama, S.; Honda, K.; Uzawa, J.; Nishio, M. CH/π Interaction in the Crystal Structure of Organic Compounds. A Database Study. *Bull. Chem. Soc. Jpn.* **1998**, *71*, 1207–1213.

(10) *The CH/π Interaction: Evidence, Nature, and Consequences | Wiley*. Wiley.com. https://www.wiley.com/en-us/The+CH+%26pi%3B+Interaction%3A+Evidence%2C+Nature%2C+and+Consequences-p-9780471252900 (accessed 2023-07-19).

(11) Nishio, M. The CH/π Hydrogen Bond in Chemistry. Conformation, Supramolecules, Optical Resolution and Interactions Involving Carbohydrates. *Phys. Chem. Chem. Phys.* **2011**, *13*, 13873.
